# Chromosome‐level wild *Hevea brasiliensis* genome provides new tools for genomic‐assisted breeding and valuable loci to elevate rubber yield

**DOI:** 10.1111/pbi.14018

**Published:** 2023-02-22

**Authors:** Han Cheng, Xiaoming Song, Yanshi Hu, Tingkai Wu, Qihang Yang, Zewei An, Shuyan Feng, Zhi Deng, Wenguan Wu, Xia Zeng, Min Tu, Xiyin Wang, Huasun Huang

**Affiliations:** ^1^ Rubber Research Institute Chinese Academy of Tropical Agricultural Science Haikou Hainan China; ^2^ Key Laboratory of Biology and Genetic Resources of Rubber Tree Ministry of Agriculture and Rural Affairs Haikou China; ^3^ School of Life Sciences/Center for Genomics and Bio‐computing North China University of Science and Technology Tangshan Hebei China

**Keywords:** *Hevea brasiliensis*, genome, domestication, selective sweep, genome‐wide association study, nonsynonymous SNP

## Abstract

The rubber tree (*Hevea brasiliensis*) is grown in tropical regions and is the major source of natural rubber. Using traditional breeding approaches, the latex yield has increased by sixfold in the last century. However, the underlying genetic basis of rubber yield improvement is largely unknown. Here, we present a high‐quality, chromosome‐level genome sequence of the wild rubber tree, the first report on selection signatures and a genome‐wide association study (GWAS) of its yield traits. Population genomic analysis revealed a moderate population divergence between the Wickham clones and wild accessions. Interestingly, it is suggestive that *H. brasiliensis* and six relatives of the *Hevea* genus might belong to the same species. The selective sweep analysis found 361 obvious signatures in the domesticated clones associated with 245 genes. In a 15‐year field trial, GWAS identified 155 marker–trait associations with latex yield, in which 326 candidate genes were found. Notably, six genes related to sugar transport and metabolism, and four genes related to ethylene biosynthesis and signalling are associated with latex yield. The homozygote frequencies of the causal nonsynonymous SNPs have been greatly increased under selection, which may have contributed to the fast latex yield improvement during the short domestication history. Our study provides insights into the genetic basis of the latex yield trait and has implications for genomic‐assisted breeding by offering valuable resources in this new domesticated crop.

## Introduction

The rubber tree (*Hevea brasiliensis* Muell. Arg, 2 *n* = 36) is the most important source of natural rubber (NR), a widely used polyisoprene material. More than 90% of NR in the world is produced by rubber trees (Priyadarshan and Goncalves, 2003). The annual production is estimated to be ~14 million tons (http://www.fao.org/faostat/en/#home). Rubber trees are widely planted in Southeast Asia, West Africa and South America (approximately 13.3 million hectares). Rubber trees have become important sources of revenue for developing countries in these regions. Traditionally, the *Hevea* genus is believed to include 10 species (Clément‐Demange *et al*., 2007), exclusively located in the Amazon basin. Though the natives of the West Indies used NR for thousands of years, natural rubber industrial applications were discovered less than two centuries ago (Priyadarshan, 2017). Henry Wickham collected 70 000 seeds from Brazil in 1876 and sent the germinated seedlings to Asia (Cheng *et al*., 2020). Following this event, rubber trees were hastily disseminated and domesticated. Due to high heterozygosity, rubber tree breeding efforts started in 1918 only after the vegetative propagation procedure was successfully developed, by which the agronomical traits could maintained (Priyadarshan, 2017). Rubber trees have been domesticated for approximately one century, and only four breeding generations have been undertaken thus far (Cheng *et al*., 2020). Despite that, the yield of rubber trees has increased from 496 kg ha^−1^ in unselected trees to 3000 kg ha^−1^ in RRIM 3001, a recently released clone (Mazlan *et al*., 2021; Priyadarshan, 2017). Benefiting from clone improvement, Asia now produces approximately 92% of the NR in the world (http://www.anrpc.org/). The original Wickham stock was collected in only one site (Boïm, on Brazil's Western banks of the Tapajos river). It was observed that, following one century of directional selection, a low level of genetic diversity might hinder yield improvement in the Wickham genotype. To overcome this limitation, several rounds of wild germplasm collections have been conducted. However, utilization is, thus far, restricted due to the significantly low average yield of wild germplasms (Clément‐Demange *et al*., 2007).

**Figure 1:**
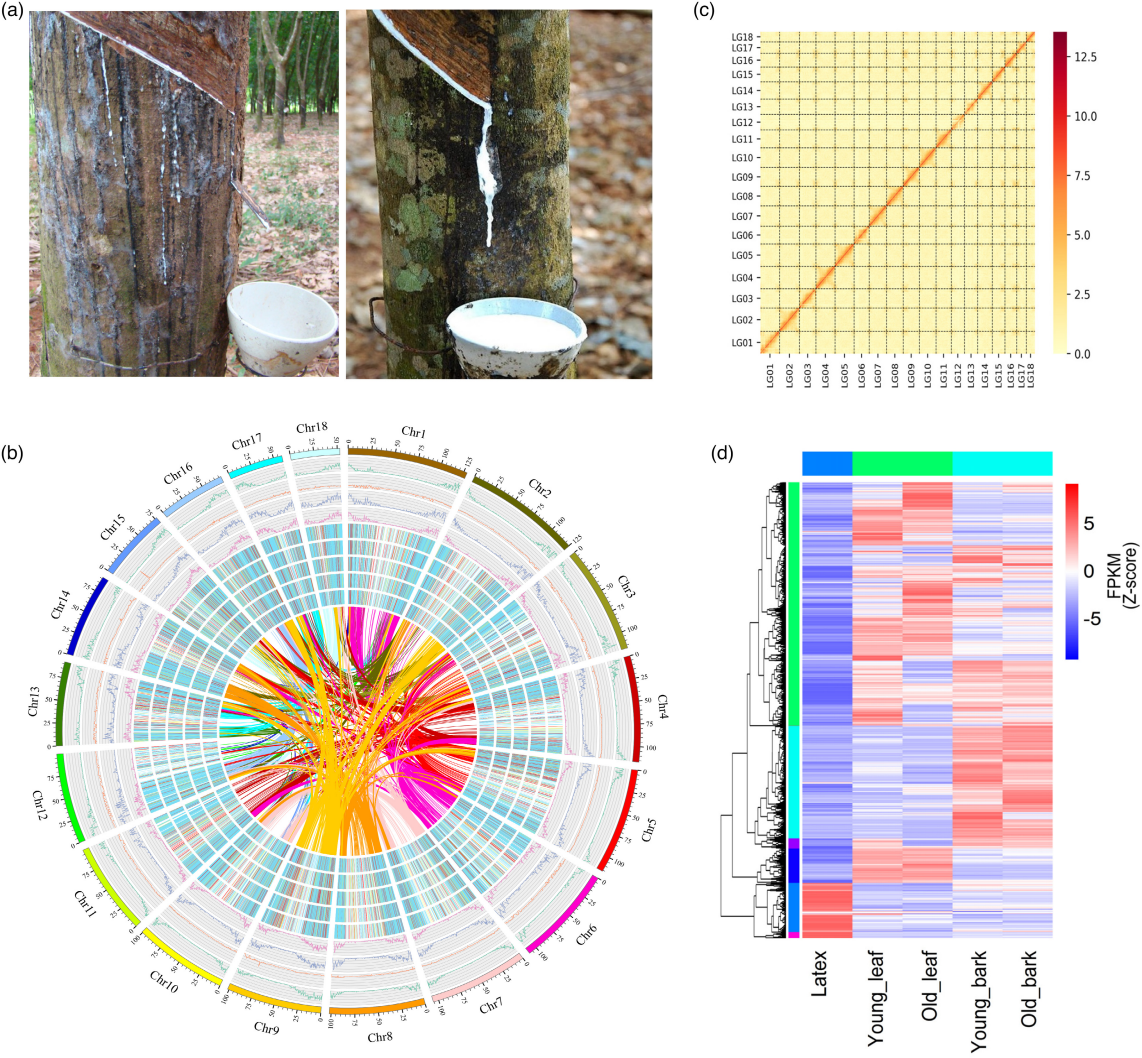
Assembly of rubber tree genome. (a) Rubber tree yield collected by tapping. Left, low‐yield wild accession MT/VB/25A 57/8; Right, high‐yield Wickham clone Reyan7‐33‐97. (b) Circos plot of repeat sequences and expression patterns in the rubber genome. The curves connect the colinear blocks between different chromosomes. The heatmap illustrates the gene expression levels (Log_2_FPKM) in young leaves, young bark, old leaves, old bark and latex of rubber (from inside to outside). The red and green colours indicate high and low expression levels, respectively. The density of line charts represents gene density, transposable element (*TE*), short tandem repeat (S*TR*) and simple sequence repeat (*SSR*) across 18 chromosomes in the rubber tree (from inside to outside). The colour density indicates a high density of interactions. Distinct chromosomes are separated by dotted lines. (c) Genome‐wide all‐by‐all highest‐throughput chromosome conformation capture (*Hi‐C*) interaction heatmap for the 18 linkage groups in *Hevea brasiliensis*. Heat map showing the density of the Hi‐C interactions between chromosomes. (d) Heatmap of the *DEGs* (differentially expressed genes) expression levels in leaf, bark tissues and latex. In total, 4192 DEGs were identified to be differentially expressed.

Unlike grain crops, which could be harvested several times annually under controlled conditions, rubber trees can be harvested only after 7–8 years as the yield depends on latex collected by tapping the bark (Figure 1a). Thus, the time to breed new genotype with traditional crossing extends to over 30 years (Supriya and Priyadarshan, 2019). Simple sequence repeats (SSR), amplified fragments length polymorphism (AFLP) and other types of molecular markers were developed to accelerate the rubber tree breeding process (Hou *et al*., 2017; Lespinasse *et al*., 2000; Mantello *et al*., 2012; Pootakham *et al*., 2020; Souza *et al*., 2013, 2019). It was observed that the environmental conditions affect flow of latex in the real‐time, thus affecting the yield via several complex interactions (and thus not only by loci that can be pinpointed with individual markers). Recently, genotyping by sequencing (GBS) and whole‐genome resequencing (WGR) strategies were used to construct genetic maps and develop numerous markers that can help in the identification of markers tightly linked with agronomic traits at the genome‐wide scale (An *et al*., 2019; Pootakham *et al*., 2015; Wu *et al*., 2022; Xia *et al*., 2018). However, a high‐quality reference genome sequence is required for precise mapping. To date, several rubber tree genome sequence assemblies have been generated using the next‐generation sequencing (NGS) approaches (Lau *et al*., 2016; Pootakham *et al*., 2017; Rahman *et al*., 2013; Tang *et al*., 2016), providing insights into the complexity of genome evolution. Recently, a chromosome‐level genome sequence of GT1 clone has been assembled (Liu *et al*., 2020) using single‐molecule real‐time sequencing (SMRT) and the Hi‐C technique, contributing to understanding the evolutionary processes of the Spurge family. However, all the sequenced accessions are Wickham clones, descendants of nine trees in Kuala Kangsar (Malaysia) after being dispatched from the Kew Garden (Priyadarshan, 2017). The Wickham clones are very closely genetically related, and at the same time, the genome of a wild accession has never been sequenced. Herein, we present a high‐quality chromosome‐level genome assembly of a wild rubber tree accession, MT/VB/25A 57/8, that provides an improved understanding of the genome architecture and adaptation process of the domesticated rubber trees and Wickham clones. One hundred and seven Wickham clones, 34 IRRDB1981′ wild accessions and six *Hevea* genus relatives were systematically compared and analysed following the genome resequencing process. These results provided further insights into the population structure of the *Hevea* genus, selection signatures, the process of genome evolution and the loci contributing to latex yield improvement.

## Results

### Chromosome‐scale genome assembly of wild rubber tree

A wild rubber tree (accession no: MT/VB/25A 57/8; Figure 1a,b) carrying a low heterozygosity rate and characterized by low rubber yield (Figures S1, S2, Table S1) was used for genome assembly. A total of 300 × single‐molecule real‐time (SMRT) long reads and 60 × paired‐end reads were generated by the Oxford Nanopore platform (PromethION) and Illumina HiSeq X Ten sequencing platform, respectively (Table S2, Figure S3). The contigs were assembled with an N50 length of 3.51 Mb, covering 1.72 Gb in total and accounting for 94.5% of the estimated rubber tree genome (Table S5, Figure S4). Following this, approximately ~264 × BioNano Irys optical map data were used to assemble the scaffolds further and generated an improved assembly containing 957 scaffolds with an N50 of 48.0 Mb. The longest scaffold covered approximately 97.1 Mb (Table S3). Finally, ~135 × chromosome conformation capture (Hi‐C) data (Table S4) were employed for the anchoring ordering and orientation of these scaffolds, yielding 18 linkage groups, harbouring 93.90% of the assembled sequences with an N50 of 102.0 Mb (Figure 1c, Tables S5, S6, Figure S5). This assembly has 23‐fold higher contig N50 length and a 10‐fold smaller contig number when compared to the recently published GT1 genome (Table S5) (Liu *et al*., 2020). Core eukaryotic gene (CEGs) and Benchmarking Universal Single‐Copy Ortholog (BUSCO) assessment showed that the wild rubber tree genome assembly covered 89.92% complete CEGs and 97.75% complete BUSCOS. (Tables S7–S12, Figures S6, S7).

1.45 Gb of repetitive sequences were identified (76.72%). Long terminal repeats (*LTRs*) accounted for 71.05% of the whole genome, including 41.58% Gypsy and 6.53% *Copia* retro‐elements (Figure 1b, Table S13). The predicted protein‐coding gene number was 35 318 (Tables S14, S15, Figures S10–S14) based on an Evidence Modeler (EVM) strategy prediction (Haas *et al*., 2008). The gene expression in leaf, bark tissues and latex was studied using RNA‐Seq, revealing 4192 differentially expressed genes. A distinct expression profile compared to the leaf and bark tissues was observed in latex (Figure 1d). In terms of noncoding RNAs (ncRNAs), 449 rRNAs, 4955 small RNAs, 923 tRNA and 30 regulatory elements were identified (Table S16).

### Population structure and genetic diversity

To investigate the population structure and diversity, 147 rubber tree accessions, including 107 Wickham clones, 34 IRRDB1981’ wild accessions and six *Hevea* genus relatives (Table S21) were re‐sequenced at a 19.98‐fold average depth coverage. After rigorous filtering, 6 222 360 high‐quality SNPs were obtained (Figures S23–S25, Tables S22–S25).

**Figure 2:**
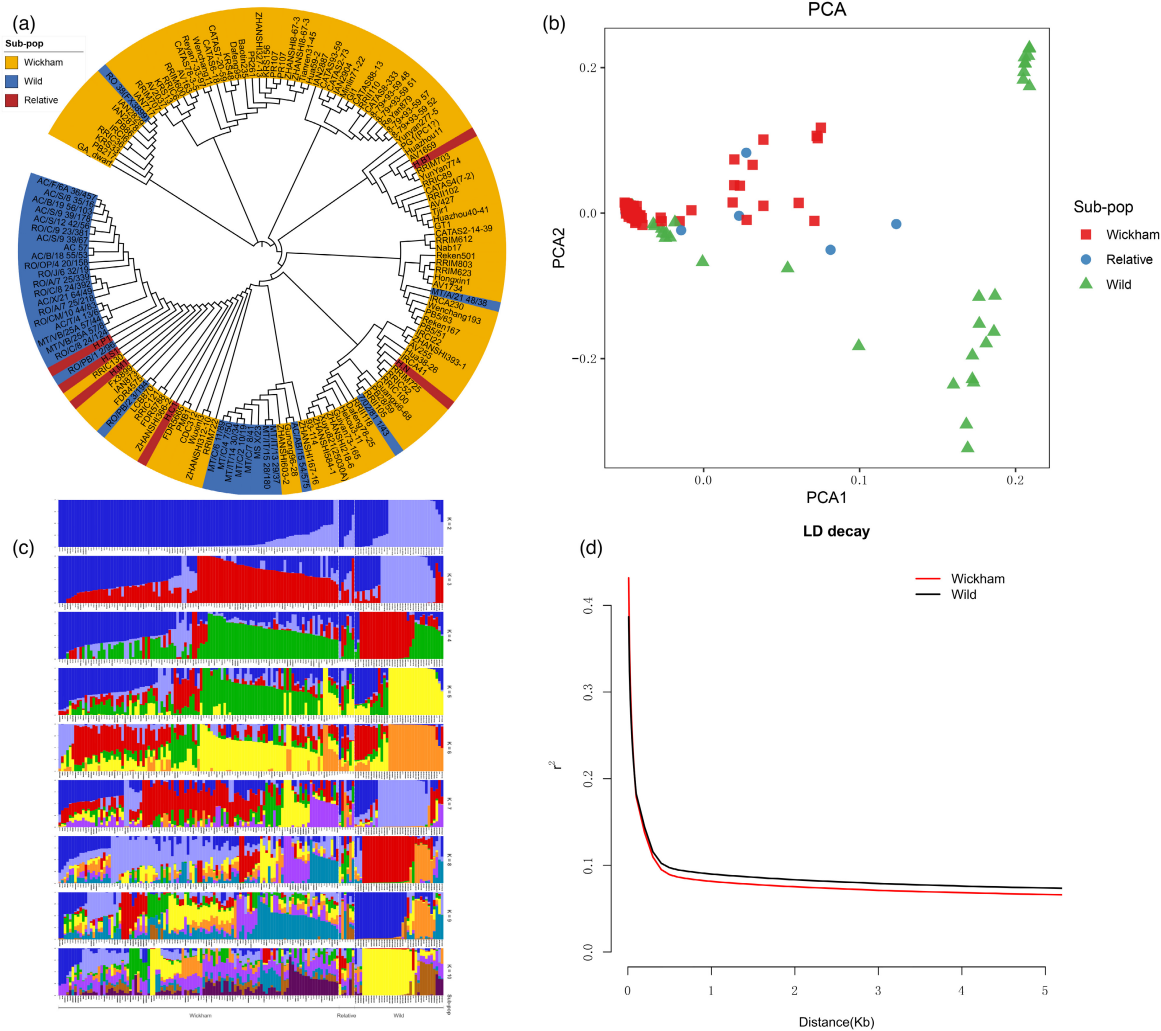
Population structure and genetic diversity in rubber trees. (a) Neighbour‐joining (NJ) phylogenetic tree of the 147 rubber tree accessions. Brown, Wickham clones; blue, IRRDB1981’ wild accessions; red, *Hevea* wild relative species. (b) Principal component analysis (*PCA*) was conducted for 147 rubber tree accessions. Triangle, Wickham clones; green square, IRRDB1981’ wild germplasm; red circle, *Hevea* wild relative species. (c) Population structure of 147 rubber tree accessions inferred by Admixture. The length of each coloured segment represents the proportion of each genome inferred from ancestral populations (*K* = 2–10). (d) Linkage disequilibrium (LD) decay in the rubber tree‐Wickham clones and IRRDB1981’ wild accessions. Red‐coloured line, LD decay in Wickham clones; black‐coloured line, LD decay in IRRDB1981’ wild accessions.

The rubber tree was recently domesticated, and the most planted clones are derived from the Wickham germplasm background. The phylogenetic relationships were explored by examining genome‐wide genetic variations, following the NJ method based on the Kimura‐2‐parameter model with 1000 bootstrap replications. Surprisingly, the phylogenetic tree constructed indicated that, although the Wickham clones, wild accessions and the wide relative species were roughly separated, no distinct cluster could be defined for the three germplasm groups. These results are in agreement with previously reported findings (de Souza *et al*., 2018). Most of the Mato Grosso (MT) accessions were closer to the Wickham clones in the phylogenetic tree (Figure 2a). In addition, the six *Hevea* genus wild relative species were also mixed within the wild accessions and the Wickham clones (Figure 2a), showing the nondistinguishing species boundaries. This could be explained by the short domestication history of rubber trees and the high gene flow rate between different germplasm types (Clément‐Demange *et al*., 2007). Furthermore, the population's genetic affinities were assessed by performing a principal component analysis (PCA). As expected, the results did not reveal a clear genetic structure among different germplasm types, although the Wickham clones and IRRDB1981’ wild accessions exhibited discrete clustering patterns (Figure 2b, Figure S26). These findings were further supported by the ADMIXTURE analysis. The optimum number of genetic clusters (*K*) was 5 (with the lowest cross‐validation error, Figure S27). However, Wickham clones, wild accessions and relative species could be found in each cluster (Figure 2c). We assumed *K* values of 2 and 3 to evaluate the population structure. No distinct segregation pattern was observed among the IRRDB1981’ wild accessions, Wickham clones and relatives (Figure 2c). This further validated the presence of a strong gene flow within the *Hevea* genus and suggested that the six *Hevea* genus relative species, assigned by classical taxonomy, may actually belong to the same species.

The population linkage disequilibrium (LD, indicated as *r* ^2^) decay rate and nucleotide diversity (π) were determined in 34 wild accessions and 34 randomly selected Wickham clones. A short LD pairwise distance (dropping to half of its maximum value) and a low baseline were observed (Figure 2d). We inferred that the fast LD decay should be attributed to the high heterozygosity in the rubber tree and the high SNP density identified in the present study. The π values in the Wickham clones (8.19E‐04) and wild accessions (1.08E‐03) were also comparable (Figures S28–S30). The population fixation index (*F*_ST_) was inferred to be 0.1918 (Figure S31), indicating a moderate population divergence for this newly domesticated crop.

### Selection signals resulting from latex yield improvement in Wickham clones

**Figure 3:**
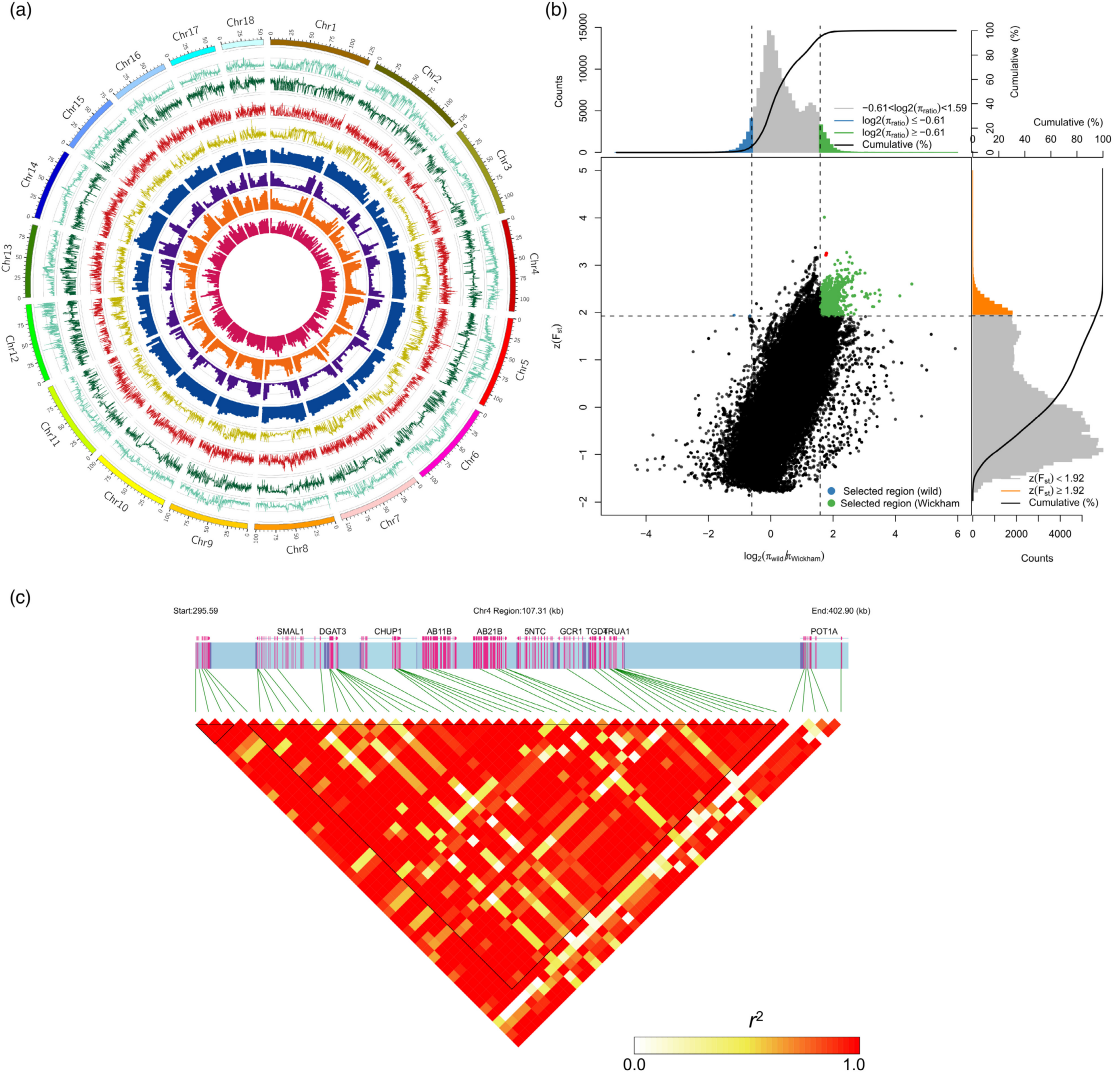
Genomic distribution of characteristics and signatures of selection in the rubber tree genomes during the breeding and improvement process. (a) Genomic distribution of genetic diversity. From inside to outside: Density of SNPs, InDels, SVs and CNVs on each chromosome; *F*_ST_ (*fixation index*) between Wickham and wild clones, nucleotide diversity (π), neutral evolutionary test (Tajima's D) and linkage disequilibrium within 100‐kb windows along the chromosomes. (b) Distribution of population differentiation (*F*_ST_) and π ratios (log10(_π_^wild^/_π_^Wickham^)) determined for IRRDB1981’ wild accessions and Wickham clones using 100‐Kb sliding window and 10‐Kb steps. Dots in the upper right (green) side are genomic regions under positive selection (high population differentiation between the two populations and low nucleotide diversity within the Wickham clones). Threshold, >95%. (c) Linkage disequilibrium of the nonsynonymous SNPs in the selective sweep region on Chr4 (290001–410000) corresponding to the red dots on B.

The latex yield in the rubber tree clones has increased almost sixfold since the beginning of their cultivation in Asia (Othman, 2018). To reveal the genetic patterns that arose during the clone improvement process, we scanned the genome to identify regions with high allele frequency divergence and maximum differences in the nucleotide diversity using 100‐Kb sliding windows with 10‐Kb steps (Figure 3a). In the Wickham clones, regions under positive selection displayed high population differentiation (*F*_ST_) and low nucleotide diversity (π). The top 5% threshold was set for *F*_ST_ and π^wild^/π^Wickham^ to screen the potential regions that had undergone a positive selection. A total of 361 loci were found in the regions that displayed positive selection in the Wickham clones (Z(*F*_ST_) ≥ 1.92; log2 (π^wild^/π^Wickham^) ≥1.59) (Figure 3b, Table S26). In total, 245 genes (approximately 0.70% of the annotated genes) were identified in these selective regions (Table S27), in which 162 genes contained 580 nonsynonymous SNPs (nsSNPs) causing amino acid changes in their peptide sequences (Table S28). These nsSNPs in genes might correspond to potential gain or loss of function protein products and may contribute to fast latex yield improvement during a short domestication history in rubber trees.

The genomic regions with high *F*_ST_ and π ratio (wild/Wickham) were carefully investigated, and a 120‐Kb fragment was identified on Chr4 (290001–410000) containing 51 nsSNPs (Figure 3c). The region's highest *F*_ST_ and π ratio (wild/Wickham) were inferred to be 3.1 and 2.27, respectively (red points in Figure 3b), showing a strong selection signal. A total of 11 genes are located in this region, among which 10 contain nsSNPs (Figure 3c, Table S29). The frequencies of 38 nsSNP were significantly increased in Wickham clones than in wild accessions, 10 were decreased, and three were unchanged. Interestingly, all 48 nsSNPs with frequency changes are homozygous (Table S29). The linkage disequilibrium plot defines large haplotype blocks in this region. Both the total SNPs (Figure S32) and 51 nsSNPs (Figure 3c) displayed high LD values (*r* ^2^), suggesting that homozygous SNPs in this block constitute a haplotype with underlying functions that have contributed to the rubber tree domestication.

### Genome‐wide association study to identify genes associated with the latex yield

Based on latex yield data collected from 2017 to 2021 when the trees were 10 years old (Table S30), we performed GWAS analysis using a Settlement of MLM Under a Progressively Exclusive Relationship (SUPER) model (Wang *et al*., 2014). A *P*‐value of 1.60 × 10^−7^ was set as the significance threshold after a Bonferroni correction (Figures S33–S34). The significant SNPs within a 10‐Kb region are considered as a locus, and the SNP with the lowest *P*‐value in a locus is defined as the SNP most closely linked to the causal genes. As a result, a total of 155 marker–trait associations (MTAs) were detected (*P*‐value <1.60E‐07; Table S31). To identify candidate genes associated with latex yield, we searched for genes within 10 kb of the most significant SNPs and covering at least 10% of a gene's length. Totally, 326 genes were obtained and represent plausible latex yield improvement candidates. Among these MTAs, 100 were located outside of the gene bodies (upstream‐, downstream gene and intergenic region), while 55 were within the gene sequences (UTR, intron, synonymous and nonsynonymous), including 20 nsSNPs (Table S31). The associated genes identified by GWAS were compared with those that had undergone a selective sweep, with a total of nine genes shared in both sets (Table S32).

**Figure 4:**
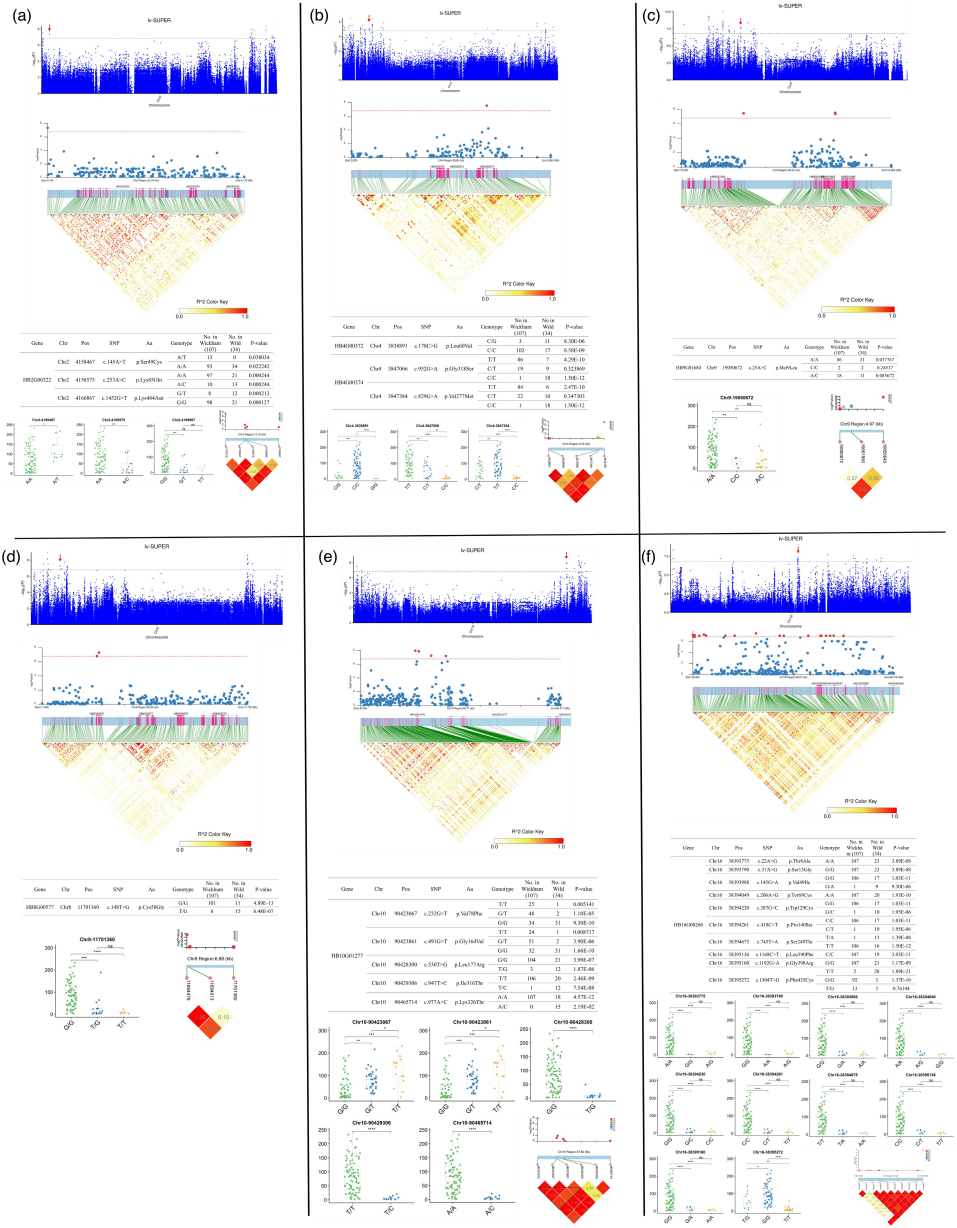
GWAS identification of candidate genes related to sugar transport and metabolism associated with latex yield. (a) *HB2G00322*, encoding a glucose‐6‐phosphate isomerase. (b) *HB4G00372* and *HB4G00374*, encoding the sugar transport protein 8 and the sugar transport protein 13, respectively. (c) *HB9G01684*, encoding the bidirectional sugar transporter SWEET17. (d) *HB8G00577*, encoding a fructose‐1,6‐bisphosphatase. (e) *HB10G01277*, encoding an ATP‐dependent 6‐phosphofructokinase 5. (f) *HB16G00266*, encoding a sugar carrier protein C. From top to bottom in each panel: (1) Manhattan plots for latex yield on each chromosome using the SUPER model; (2) Linkage disequilibrium block plots around the statistically significant signals of marker–trait associations; (3) Lists of SNPs resulting in amino acid changes in the coding sequence of each gene. The *P*‐values indicate the significance of Fisher's exact tests for the genotype frequencies in the Wickham clones; (4) Box plots for latex yield in the accessions with the indicated genotypes. The box plots represent the interquartile range. The middle line represents the median, and the box limits indicate the upper and lower quartiles. The circle dots show the latex yield of each accession, and the significance of difference was calculated with a two‐tailed *t*‐test (ns, no significance; *, **, ***, ****, significance with *P* < 0.05, 0.01, 0.001 and 0.0001 respectively); (5) Representation of pairwise *r* ^2^ values between the significant SNPs of marker–trait associations and the nonsynonymous SNPs of each gene, with the colour intensity of each box and *r* ^2^ values according to the legend. Circles represent nonsynonymous SNPs and diamonds represent significant SNPs.

We chose loci for further investigation, which are associated with sugar transport and metabolism or ethylene biosynthesis and signalling genes. Sugars are the carbon source for natural rubber (polyisoprene) biosynthesis and are transported from the source tissues (leaf) to the sinks (laticifer) and utilized through glycolysis (Dusotoit‐Coucaud *et al*., 2009; Liu *et al*., 2015; Long *et al*., 2015; Sui *et al*., 2017). In total, six MTAs correlated with sugar transport and utilization (three with sugar transport and three with glycolysis). The genes, *HB4G00372*, *HB4G00374*, *HB9G01684* and *HB16G00266*, encoding sugar carriers and transporters, were found in proximity to the significant SNPs for yield (Figure 4b,c,f). The SNPs *Chr4‐3848180* and *Chr16‐38395272* were located within the genes *HB4G00374* and *HB16G00266*, causing synonymous and missense variants, respectively (Figure 4b,f). At the same time, *HB9G01684* is ~3.4‐Kb upstream of the significant SNP *Chr9‐19 055 645* (Figure 4c). For the loci associated with sugar metabolism, the genes in proximity were *HB2G00322* (~3.4‐kb downstream to *Chr2‐4153720*, encoding glucose‐6‐phosphate isomerase), *HB8G00577* (~6.4‐kb downstream to *Chr8‐11 694 479*, encoding fructose‐1,6‐bisphosphatase) and *HB10G01277* (~14.5‐kb downstream to *Chr10‐90 407 872*, encoding ATP‐dependent 6‐phosphofructokinase 5) (Figure 4a,d,e, Table S33). We further investigated the missense variants located within these genes, and the SNPs with significant frequency differences between the Wickham clones and the wild accessions subgroups are listed in Figure 4. The LD values between the significant SNPs and missense SNPs of these genes were assessed, and all displayed high *r* ^2^ (>0.6), indicating the close linkage of the SNPs to these alleles (Figure 4a–f). The accessions were separated by genotypes, and the latex yield, compared with each SNP, showed significant differences among all these comparisons in the five continuous years of tapping trials (Figure 4a–f). The *HB16G00266* gene alleles were the most striking ones, as almost all the Wickham clones shared the same genotypes in nine out of 10 nsSNPs, and these alleles displayed complete LD (*r* ^2^ = 1) with the significant latex yield associated SNP (Figure 4f). The accessions harbouring these haplotypes showed significantly higher latex yield than the other haplotypes identified (Figure 4f).

**Figure 5:**
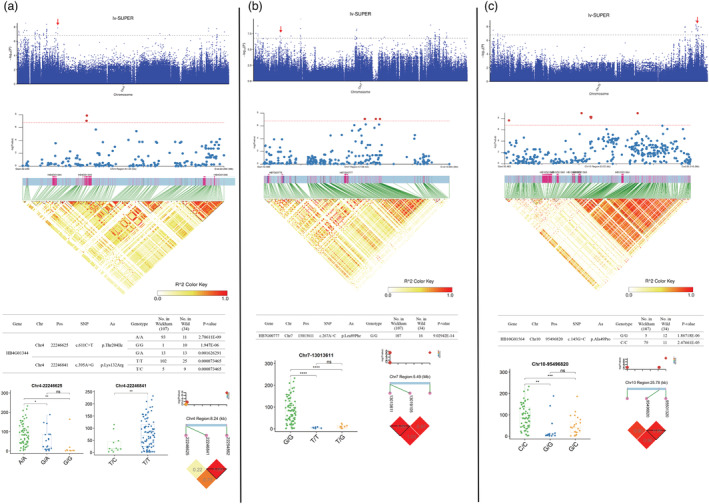
GWAS identification of candidate genes related to ethylene biosynthesis and signalling associated with latex yield. (a) *HB4G01344*, encoding an AP2/ERF and B3 domain‐containing transcription factor. (b) *HB7G00777*, encoding a 1‐aminocyclopropane‐1‐carboxylate oxidase. (c) *HB10G01563* and *HB10G01564*, encoding an ethylene‐responsive transcription factor, respectively. From top to bottom in each panel: (1) Manhattan plots for latex yield on each chromosome using the SUPER model; (2) Linkage disequilibrium block plots around the statistically significant signals of marker‐trait associations; (3) Lists of SNPs resulting in amino acid changes in the coding sequence of each gene. The *P*‐values indicate the significance of Fisher's exact tests for the genotype frequency in the Wickham clones; (4) Box plots for latex yield in the accessions with the indicated genotypes. The box plots represent the interquartile range. The middle line represents the median, and the box limits indicate the upper and lower quartiles. The circle dots show the latex yield of each accession, and the significance of difference was calculated with a two‐tailed *t*‐test (ns, no significance; *, **, ***, ****, significance with *P* < 0.05, 0.01, 0.001 and 0.0001 respectively); (5) Representation of pairwise *r* ^2^ values between the significant SNPs of marker–trait associations and the nonsynonymous SNPs of each gene, with the colour intensity of each box and *r* ^2^ values according to the legend. Circles represent nonsynonymous SNPs and diamonds represent significant SNPs.

Ethylene plays an essential role in stimulating latex yield in rubber trees (Lestari *et al*., 2018; Liu, 2016; Montoro *et al*., 2018; Putranto *et al*., 2015; Zhang *et al*., 2021; Zhu and Zhang, 2009). Three MTAs were associated with genes related to ethylene biosynthesis and signalling. The *HB7G00777* gene, encoding the final enzyme of ethylene biosynthesis, 1‐aminocyclopropane‐1‐carboxylate oxidase (ACO), was found in proximity with *Chr7‐13019105*. An SNP (*Chr7‐13019105*) causing a missense mutation (Leu^89^ to Phe) at 267 nt of the cDNA sequence was found. Compared with wild accessions containing T/T (7), T/G (11) and GG (16) genotypes, all the Wickham clones harboured a G/G (107) genotype. Moreover, the latex yield in the accessions carrying the G/G genotype was significantly higher than in the accessions carrying a T/T and T/G genotypes (Figure 5b). *HB4G01344* and *HB10G01564*, encoding ethylene‐responsive transcription factors (ERF), were found in proximity with to two other strong association signals (with ‐log_10_ *P* value of 7.86 and 8.93, and distance of 7.6 kb and 4.0 kb, respectively). In *HB4G01344*, two missense SNPs were found, resulting in the change of Thr^204^ to Ile and Lys^132^ to Arg, respectively. The Wickham clones exhibited significantly higher frequencies of the A/A (93/107) and T/T (102/107) genotypes at these two alleles. Moreover, the latex yield in the accessions carrying the A/A or T/T genotypes was significantly higher than the accessions carrying other genotypes (Figure 5a). In *HB10G01564*, one missense SNP was identified at *Chr10‐95496820*, changing Ala^49^ to Pro. The homozygous C/C frequency was significantly higher in the Wickham clones than in wild accessions. The latex yield in the accessions carrying the C/C allele was significantly higher than the accession harbouring other genotypes (Figure 5c). The LD values (*r* ^2^) between the significant SNPs and the missense alleles were evaluated and a very close linkage was found (Figure 5a–c).

### NsSNPs homozygosity was increased under selection

**Figure 6:**
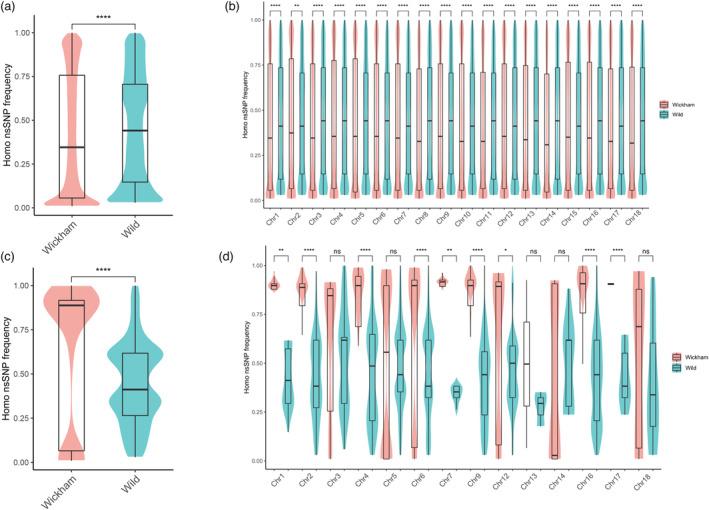
Homozygosity of the nonsynonymous SNPs was increased under selection. (a–c) Box plots of the frequencies of homozygous no‐synonymous SNPs in the whole genome and the selective regions. (b–d) Box plots of the frequencies of homozygous nonsynonymous SNPs in all chromosomes and in selective regions of each chromosome. Wickham and Wild denote the frequencies in the Wickham (red) and wild (blue) accessions. The significance of differences was calculated with a two‐tailed *t*‐test (ns, no significance; *, **, ***, ****, significance with *P* < 0.05, 0.01, 0.001 and 0.0001 respectively).

Interestingly, we observed that the homozygous genotype frequencies were increased in nsSNPs associated with selection and GWAS signals (Table S29, Figures 4, 6). These frequencies were then calculated in the Wickham and wild accessions. 126 371 nsSNPs were identified in total, including 383 285 homozygous genotypes among these alleles (346 285 in the Wickham clones and 346 670 in the wild accessions). The median frequency values of the homozygous genotypes were comparatively lower in the Wickham clones than in the wild accessions in both the genome and chromosome scales (Figure 6a,b). However, the upper quartiles of the frequency were higher in the Wickham clones in the whole‐genome scale and most of the chromosomes (except Chr14 and Chr17). This indicated that the major homozygous genotype frequencies had been increased in the nsSNP alleles during the domestication.

The nsSNPs homozygosity in the selective regions was also inspected. Surprisingly, the median and upper quartile values of the frequencies of all homozygous nsSNPs under selection were greatly increased in the Wickham clones (Figure 6c). When the homozygosity frequencies were calculated in individual chromosomes, it was found that both the median and the upper quartile values were greatly increased in Wickham clones and significantly higher than in wild accessions in most chromosomes (Figure 6d, except Chr3, 5, 13, 14, 18). As these nsSNPs are under positive selection (Figure 3b), their increased homozygosity would alter the encoded proteins' activity that was gained by amino acid substitutions. It also suggests the positive roles of these nsSNPs for latex yield improvement.

### Two variant ERFs have strong transactivation properties

**Figure 7:**
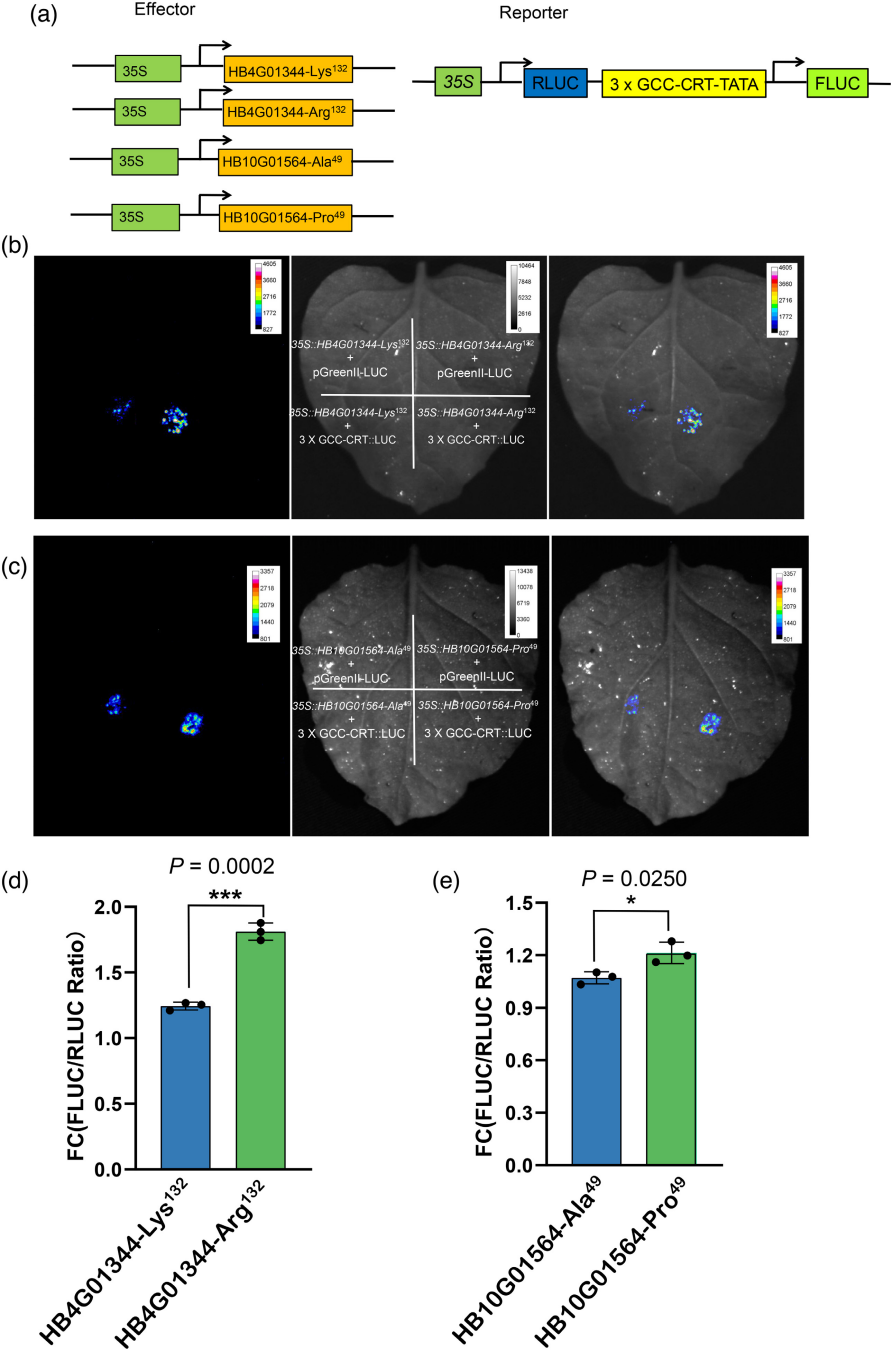
Dual‐luciferase transient expression assay in *Nicotiana benthamiana* leaves showing strong transactivation property in two ERFs genes containing homologous nsSNPs. (a) Diagrams of a reporter in pGreenII 0800‐LUC vector and effectors of *Hb4G01344* and *Hb10G01564* genes' variants. The superscripts after Hb4G01344 and Hb10G01564 refer to positions and amino acid changes caused by the nonsynonymous SNPs. RLUC, renilla luciferase; FLUC, firefly luciferase. (b, c) Representative FLUC signals detected by transient expression assay for *Hb4G01344* and *Hb10G01564*. For the control and experiment, the pGreenII 0800‐LUC empty vector (pGreenII::LUC) and reporter vector (3 × GCC‐CRT::LUC) were co‐infiltrated with effector vectors, respectively, as indicated on the leaves of the central panel. Left of each panel, signal captured at 550–570 nm; Central, brightfield; Right, merged. (d, e) The comparison of LUC activity. The transcriptional activity based on the ratio of FLUC and RLUC was measured with a dual‐luciferase reporter gene assay kit. Each value represents the mean ± SE of three biological replicates. Significances were calculated by Student's *t‐*test (*, ***, significance with *P* < 0.05 and 0.001 respectively).

To test the hypothesis that nsSNP would alter a protein's activity, two identified ERF genes (*Hb4G01344* and *Hb10G01564*) were assessed by transactivation activity tests using dual‐luciferase assays in *Nicotiana benthamiana* leaves. Each gene contains nonsynonymous alleles resulting in two variants with amino acid changes of Lys^132^Arg (*Hb4G01344*) and Ala^49^Pro (*Hb10G01564*) respectively. A synthetic AP2/ERF responsive promoter (three tandem repeats of GCC and CRT boxes, with minimal TATA region of the CaMV 35S promoter) was used to drive firefly luciferase (FLUC) genes in the pGreenII 0800‐LUC plasmid. The reporter vector was co‐infiltrated into *N. benthamiana* leaves with effector vectors harbouring *Hb4G01344* and *Hb10G01564* gene variants (Figure 7a). As shown in Figure 7b,d, when *Hb4G01344* was co‐infiltrated, the LUC signal was significantly higher in the variants carrying Arg^132^ (homozygous allele) than their counterparts carrying Lys^132^. For *Hb10G01564*, the homozygous allele variant carrying Pro^49^ also exhibited significantly strong transactivation properties when compared to the Ala^49^ allele variant (Figure 7c,e). These findings clearly demonstrated that the nsSNPs resulted in protein activity changes in *Hb4G01344* and *Hb10G01564* and that the homozygous genotype exhibit strong transactivation properties.

## Discussion

The rubber tree is a newly domesticated tropical crop with a 147‐year‐old cultivation history and has undergone artificial breeding and selection for approximately a century. Most of the recommended clones grown in Southeast Asian countries are the Wickham germplasm derived from nine founders. Thus, the cultivated rubber tree genetic background is relatively narrow, having undergone a significant genetic bottleneck. Though there were reports exploring Wickham population structure using SSR markers or by GBS (de Souza *et al*., 2018; Vu *et al*., 2020), a more accurate determination by deep resequencing is currently missing. All the published rubber tree genomes belong to Wickham clones (Lau *et al*., 2016; Liu *et al*., 2020; Pootakham *et al*., 2017; Rahman *et al*., 2013; Tang *et al*., 2016), and a high‐quality genome assembly from wild rubber tree would provide additional insights, as the Wickham clones went through a genetic bottleneck event during the domestication process. Statistically, the newly compiled wild genome sequence is significantly improved than those previously reported (Table S5) and provides valuable genetic resources to further advance rubber tree research and improvement.

It is intriguing whether the Wickham clones have developed a distinct subpopulation after being transferred into Southeast Asia. Phylogenetic and principal component analysis failed to reveal a distinct subgroup boundary between the Wickham clones and wild accessions. This can be potentially attributed to the short domestication history of rubber trees due to strong gene flows between the populations. A previous investigation into the rubber tree population structure, albeit with a small plant sample and low sequencing depth, suggested the wild and Wickham clones were mixed (Liu *et al*., 2020). By implementing GBS approach to characterize the genetic relationship among the rubber tree population consisting of 626 accessions, it was found that the population could be largely clustered into two groups, and the wild accessions obtained from Mato Grosso were grouped with the Wickham clones (de Souza *et al*., 2018). These studies indicated that rubber trees had not been differentiated into distinct subgroups, which usually occurs after thousands of years of evolution.

Traditionally, it was believed that the *Hevea* genus comprises 11 species in by classic taxonomy (Priyadarshan, 2016). However, a natural hybridization between *Hevea brasiliensis* and *Hevea nitida* (or other relative species) was observed, and many hybrids were produced. This phenomenon raises a debate about whether the *Hevea* genus is a superspecies or whether these differentially taxonomically assigned species actually belong to the same species. Here, we provide molecular evidence to show that the six ‘relative species’ are highly likely the same species as *Hevea brasiliensis*.

The latex yield has increased five‐ to sixfold during the first decades of rubber breeding, but this growth trend has slowed down dramatically in recent decades (Ong *et al*., 1983; Subramaniam, 1975). Breeders argued that poor genetic resources and inbreeding depression in Wickham clones restricted the improvement of yield potential in *Hevea* (Clément‐Demange *et al*., 2007; Othman, 2018; Priyadarshan *et al*., 2009). However, a genome‐scale genetic evaluation of the Wickham clones has not been conducted thus far, which would provide solid molecular evidence to support or against these assumptions. Population structure analysis demonstrated that the diversity between the Wickham clones is very limited. Here, we report for the first time on the selective sweep signatures present in Wickham clones. A total of 361 loci and 245 genes were identified, which may form the genetic basis for yield improvement. Among these genes, 162 contain nsSNPs, demonstrating that selective sweeps have profoundly impacted on the Wickham clones. A notable strong selective signal was found at *Chr4:290001–410000*. This 120‐Kb region contains 10 genes with a high nsSNP frequency, suggesting this region might played essential roles during domestication. The genes in this region are worthy of further investigation.

A GWAS was carried out with 5‐year continuous tapping data in a 15‐year field trial. One hundred fifty‐five MTAs were identified in total, among which 20 nsSNPs were located in the coding regions of the associated genes. Most notably, six MTAs were related to sugar transport and metabolism, and four were related to ethylene biosynthesis and signalling. Sugar provides the carbon source for natural rubber biosynthesis, and high sucrose content in the latex is an index for high‐yield potential in rubber trees (Tupý, 2018). Sugar is synthesized in the leaves and needs to be transported into laticifers for rubber biosynthesis. The identification of MTAs related to sugar transport and metabolism further suggests an involvement of these genes and pathways in the latex yield trait and yield potential. Ethylene is widely applied in rubber tree plantations and is the most effective stimulant for increasing latex yield (Zhu and Zhang, 2009). However, ethylene signalling is a complex network with hundreds of transcriptional factors involved (Duan *et al*., 2013; Schaller and Kieber, 2002). one hundred fifteen ERFs were identified in the rubber tree genome, most of which have not yet been characterized (Duan *et al*., 2013; Piyatrakul *et al*., 2014). Several reports suggested that ERF were involved in latex production regulation by prolonging latex flow, regulating rubber biosynthesis and facilitating laticifer differentiation (Guo *et al*., 2014; Lestari *et al*., 2018; Putranto *et al*., 2015). The identification of two additional ERF genes associated with yield may provide hints for dissecting the signalling network regulating latex production in rubber trees.

The zygosity of the nsSNPs could also have biological significance during domestication. In rubber trees, the domestication process was mainly centred around the selection for high latex yield. As each nsSNP could lead to possible protein activity differences (Figure 6), the homozygote of the activity‐changing nsSNP would further enhance the altered activities. As the rubber tree is highly heterozygous, a fast approach to improve the latex yield during the early domestication stage is to select nsSNP with high activity and fix them in a homozygous state. It also suggests that these 162 genes with 580 nsSNPs are very important for enhancing latex yield in rubber trees. Two ERFs were used as examples whose nsSNP variants exhibited transcriptional activity enhancement (Figure 7). However, more tests on the candidate genes would provide more evidence explaining the rubber domestication process and latex yield increase.

The presented rubber tree genome assembly and population study provide valuable datasets and information on development of efficient germplasm resources and new molecular breeding strategies. The insights from the population and GWAS analysis results presented here can further improve conventional breeding approaches and help introduce new breeding methods to further elevate the NR yield.

## Conclusions

This study presents a high‐quality, chromosome‐level genome sequence of wild rubber trees (accession no: MT/VB/25A 57/8), elucidates the genome evolution and population structure and provides a genetic variation map for 147 wild and cultivated clones. We identified 361 selection signatures and 155 loci associated with latex yield and revealed the contribution of the homozygosity of causal nonsynonymous SNPs on the latex yield trait improvement. Notably, six genes related to sugar transport and metabolism, and four genes related to ethylene biosynthesis and signalling were associated with latex yield. These findings provide insights into the genetic basis of the latex yield trait and offer valuable resources for improving the rubber tree and other newly domesticated crops.

## Materials and methods

### Plant materials

The rubber tree wild germplasm (IRRDB accession no.: MT/VB/25A 57/8) was used for *de novo* genome sequencing. The accession was collected from the Vila Bela district, Moto Crosso States, Brazil. The accession is characterized by low heterozygosity. One hundred and seven Wickham clones, 34 IRRDB 1981’ wild germplasm accessions and six species of the *Hevea* genus were used for a population study. The wild germplasms were primarily collected from Acre (AC), Moto Crosso (MT) and Rondonia (RO) in Brazil (IRRDB 1981 expedition). We selected 11 RO, 10 MT, 11 AC and two other accessions to study the germplasm evolution. These accessions represent the genetic diversity in the regions of *Hevea* origin and the major rubber tree plantations. The rubber trees are planted in the National Rubber Tree Germplasm Repository (Danzhou, Hainan, 19°52′N, 109°50′E, South China, 2006–2007).

### Library construction and sequencing

Illumina sequencing, nanopore sequencing and optical genome map construction were performed at Nextomics, China. High molecular weight DNA was extracted, and a nanopore sequencing library was constructed per the protocol instructions. Following this, the sequencing was conducted on the PromethION platform (Oxford Nanopore Technologies, Oxford Science Park, OX4 4DQ, UK). Illumina Sequel sequencing was performed on the NovaSeq 6000 platform. The Bionano optical genome map was constructed by capturing the images using methods outlined by the NanoChannel Array technology (Irys system; BioNano Genomics, San Diego, California, USA). The Hi‐C library was prepared following a previously reported protocol (Belton *et al*., 2012).

### Genome assemble and annotation

The *de novo* genome assembly and annotation were carried out according to the procedures in Notes S1 and S2.

### Genome comparison, collinearity analysis and gene family phylogenetic analysis

Whole‐genome comparison, homologous collinearity, gene family clustering, contraction and phylogenetic analysis were performed according to the procedures in Notes S3 and S4.

### Population resequencing and phylogenetic analysis

The rubber tree natural population (147 accessions) resequencing was conducted at Biomarker, China. The sequencing process was performed on a NovaSeq 6000 platform according to the standard protocol outlined by Illumina. The raw reads were filtered to eliminate the reads with adapters (Phred score < 10, or N > 10%). The clean reads were then mapped to the compiled reference genome using BWA (Li and Durbin, 2009), and the statistics were assessed. The SNPs were called using an accelerated GATK HaplotypeCaller program (McKenna *et al*., 2010). The SNP calling was accelerated using a Sentieon‐genomic pipeline (version: sentieon‐genomics‐202112) (Freed *et al*., 2017). For single‐sample SNP and genotype calling, several filtering steps were performed to remove: (1) InDels with quality scores <30, (2) SNPs with more than two alleles, (3) SNPs at or within 5 bp from InDels, (4) SNPs with genotyping quality scores (GQ) <10 and (5) SNPs with extremely low (<one‐third average depth) or extremely high (>threefold average depth) coverage. The obtained SNPs were finally annotated with SnpEff v4.3t (Cingolani *et al*., 2012).

For the population study, the VCF files were filtered to remove InDels, nonbiallelic SNPs and SNPs with a missing rate >90%. Population structure was analysed using the maximum‐likelihood approach implemented in ADMIXTURE v1.3.0 (Alexande*r* et al., 2009). Individual‐based clustering analysis was conducted and cross‐validated to explore convergence. The optimum number of clusters (*K*: 2–10; optimum *K* value was indicated by the minimum cross‐validation error) was determined. The subgroup value (*k*) was preset for the SNPs selected using PLINK2 v2.00a3LM to construct the neighbour‐joining tree (Chang *et al*., 2015). The phylogenetic tree was constructed using the Kimura 2‐parameter model using the MEGA5 software in the presence of 1000 bootstrap replicates (Tamura *et al*., 2013). The interactive tree of life (iTOL, https://itol.embl.de) tool was used to display the neighbour‐joining tree. PCA for all SNPs was conducted using EIGENSOFT (Price *et al*., 2006), using the default parameters.

### Genome‐wide genetic diversity analysis and selection loci identification

For each subgroup dataset, *F*_ST_ and nucleotide diversity (θ_π_) were calculated in 100‐Kb sliding windows with 10‐Kb steps using VCFtools v0.1.16 (Danecek *et al*., 2011). *F*_ST_ was used to evaluate the tolerance of genomic differentiation on candidates between two subgroups. θ_π_ was used to estimate the genomic diversity of the genotypes in each subgroup. Subsequently, the θ_π_ ratios were calculated for the two subgroups for each sliding window. The *F*_ST_ and the θ_π_ ratio were used to detect the putative selection targets by designating the top 5% of the log‐odds ratios for both θ_π_ and *F*_ST_. We analysed the cultivated clones and wild accessions and identified the loci that underwent selective sweeps during domestication.

### Genome‐wide association study

The latex yield traits were measured by tapping 10‐year‐old trees. Five biological replications were conducted for each accession. A scion of 147 accessions was grafted onto the common rootstock and planted in a 3 × 7 m spacing in 2007. The latex yield was collected by tapping the bark when the trees were 10 years old. The tapping was conducted as 3d 1/2S from May to December avoiding rain and other adverse weather conditions. Data were collected from 120 tapings from 2017 to 2021. The yield data were pretreated with the BLUP model and used for GWAS analysis with different models (GLM, MLM, MLMLM, CMLM, ECMLM, SUPER, FarmCPU, Blink, FaST, EMMA, EMMAx *etc*., Figures S35–S36). The results were visualized with the ggplot2 package in the R environment, and the thresholds for significance were set to 1.60e‐07.

### Gene functional annotation

The genomic regions that underwent a putative selective sweep were investigated to identify genes under selection pressure. The candidate genes were annotated in functional categories based on GO and KEGG databases. The enriched GO terms of the genes that underwent positive or negative selections were assessed using the BiNGO 3.03 (a plugin tool in Cytoscape 3.2.1) (Maere *et al*., 2005) with GO_full categories. Overrepresented GO categories were enriched following a hypergeometric test. The significance threshold was set as 0.05 after a *Benjamini* and *Hocheberg* FDR correction (Benjamini and Hochberg, 1995).

### Dual‐luciferase reporter assay in *Nicotiana benthamiana* leaves

For transcriptional activity analysis of gene variants, the whole coding fragments of effector genes and the promoters of the reporter constructs were synthesized at Tsingke Biotechnology Co. (Beijing, China) and subcloned into pCAMBIA2301 and pGreenII 0800‐LUC vectors, respectively. The reporter vector contains a firefly luciferase (FLUC) driven by three copies of the GCC and CRT binding elements (3 × GCC‐CRT) and a minimal TATA region of the CaMV 35S promoter. A renilla luciferase (RLUC) driven by the full CaMV 35S promoter in the pGreenII 0800‐LUC vector was used to normalize transient transformation efficiency. Each plasmid was transformed into the *Agrobacterium* strain GV3101 (pSoup‐P19). Co‐infiltration was performed by mixing equal volumes of *Agrobacterium* strains harbouring the effector and reporter vectors. FLUC and RLUC activities were measured after 48 h of infiltration using the dual‐luciferase assay kit (Promega, Madison, WI, USA) according to the manufacturer's instructions. The signal was recorded with Multimode Plate Reader (PerkinElmer, Waltham, MA, USA). The transcriptional activity was calculated by the FLUC and RLUC ratios. Biological triplicates were performed for each assay.

## Conflicts of interest

The authors declare that they have no competing interests.

## Author contributions

HC designed the project, conducted the population and the GWAS study, and coordinated the genome sequencing and evolution study. YH conducted the field trial and collected latex yield data. YH, MT, XZ and HH maintained the germplasm accessions. XS, QY, SF and XW performed the study of genome collinearity and gene family expansion. ZD, ZA and WW collected the samples. TW performed the biochemical analysis. HC wrote the manuscript. XW and XS read the manuscript.

## Supporting information


**Table S1.** The heterozygosity of the resequenced germplasms.
**Table S2.** Nanopore sequencing libraries and data statistics.
**Table S3.** The statistics of assembled contigs and scaffolds.
**Table S4.** Statistics of Hi‐C Paired‐end Reads.
**Table S5.** Characteristics of the wild rubber tree genome, and compared with the previous published rubber tree genomes.
**Table S6.** Statistics of 18 pseudomolecules.
**Table S7.** Statistics of CEGMA assessment.
**Table S8.** Statistics of BUSCO assessment.
**Table S9.** Statistics of NGS mapping results to the genome.
**Table S10.** Statistics of single nucleotide accuracy of the genome.
**Table S11.** Statistics of Nanopore reads mapping results to the genome.
**Table S12.** The alignment results of the Hevea genome to NT (Nucleotide Sequence) Database.
**Table S13.** Classification of TE in rubber tree genome.
**Table S14.** The protein coding gene prediction results.
**Table S15.** Comparison of the coding genes between other plants.
**Table 16.** Non‐coding RNA in rubber tree genome.
**Table S17**. Functional annotation of the rubber tree genes.
**Table S18.** Number of homologous gene pairs within and between genomes.
**Table S19.** Genes used for gene family analysis in each species.
**Table S20.** The 75 single copy gene families among 9 species.
**Table S21.** The origin of germplasms used for population study.
**Table S22.** Germplasms used in population study and statistics of population resequencing data.
**Table S23.** SNPs distribution on chromosomes.
**Table S24.** SNP number of effects by regions.
**Table S25.** Transitions and tranversions ratio.
**Table S26.** Number of loci under positive selection on chromosomes.
**Table S27.** The annotation of 245 genes under positive selection in cultivar clones.
**Table S28.** 580 positive selected SNP sites that caused missense mutation in amino acid in 162 genes.
**Table S29.** Genes with nsSNPs in Chr4:290001‐410000.
**Table S30.** BLUP value of latex yield data collected from 2017‐2021 when the trees are at 10 years old.
**Table S31.** Significant SNP signals for the latex yields (‐log10P >6.79).
**Table S32.** Genes associated with latex yield and under artificial selection.

**Note S1.** Reference genome sequencing and assembly.
**Note S2.** Reference genome annotation.
**Note S3.** Comparative genomic analysis.
**Note S4.** Gene family analysis.
**Note S5.** Re‐sequencing and phylogenetic analysis.
**Figure S1.** Heterozygosity estimation survey basing on kmer‐17 analysis. The kmer k‐mer occurrence plot of wild germplasm MT/VB/25A 57/8 indicated a very low heterozygosity rate about 0.462%.
**Figure S2.** Kmer‐17 distribution curve of 50× Sequel WGS data.
**Figure S3.** The ONT reads length distribution.
**Figure S4.** The accumulative length distribution of assembled contigs.
**Figure S5.** Hi‐C interaction heatmap of 18 clusters in *Hevea brasiliensis* genome.
**Figure S6.** CEGMA assessment results.
**Figure S7.** BUSCO assessment results.
**Figure S8.** Distribution between GC content and sequencing depth.
**Figure S9.** Distribution of divergence rate of each type of transposable element (TE) in the rubber tree genome assembly based on homology‐based prediction using RepeatModeler.
**Figure S10.** Species distribution of NR annotation from the genes of *Hevea brasiliensis*.
**Figure S11.** KEGG pathway classification of genes in *Hevea brasiliensis* genome.
**Figure S12.** KOG functional classification of genes in *Hevea brasiliensis* genome.
**Figure S13.** Gene ontology classification of genes in *Hevea brasiliensis* genome.
**Figure S14.** Venn diagram of rubber tree genes annotation between NR, GO, KOG, KEGG, Swissprot databases.
**Figure S15.** Cross‐species comparisons in the length distribution of genes, CDSs, exons, introns, and the numbers of exons and introns.
**Figure S16.** Synteny plot of the rubber tree genome.
**Figure S17.** Ks curve of Cassava (A), Rubber tree (B) and Poplar (C).
**Figure S18.** Synteny plot between the rubber tree and *M. esculenta* genome.
**Figure S19.** Genome collinearity and gene family analysis in rubber tree genome.
**Figure S21.** Evolutionary tree of gene family expansion and contraction.
**Figure S22.** Diagram showing the program for the rubber tree conventional breeding.
**Figure S23.** Statistics of polymorphisms loci identified from the population.
**Figure S24.** The distribution of SNP mutation types in the rubber tree accessions.
**Figure S25.** The annotation of SNP mutations.
**Figure S26.** The Percentage variance explained values of each component from PCA results.
**Figure S27.** (CV) plot for *K* = 2 to *K* = 10 in rubber tree accessions using Admixture.
**Figure S28.** Manhattan plot of genome‐wide nucleotide diversity (π) of Wickham accessions on each of the 18 chromosomes.
**Figure S29.** Manhattan plot of genome‐wide nucleotide diversity (π) of Wild accessions on each of the 18 chromosomes.
**Figure S30.** Manhattan plot of genome‐wide reduction of diversity (ROD) between wild and Wickham accessions on each of the 18 chromosomes. Dotted line, 1% threshold.
**Figure S31.** Manhattan plot of genome‐wide Fst between wild and Wickham accessions on each of the 18 chromosomes. Dotted line, 1% threshold.
**Figure S32.** Linkage disequilibrium of all SNPs in the selective region on Chr4 (290001–410000) corresponding the red dots on Figure 5B.
**Figure S33.** Q‐Q plot displaying the GWAS results for latex yield using a SUPER model.
**Figure S34.** Manhattan plot displaying the GWAS results for latex yield using a SUPER model. The threshold for significance is *P*‐value < 1.60e‐07 (‐log_10_ *P* > 6.7).
**Figure S35.** Q‐Q plot displaying the GWAS results for latex yield using: A, GLM; B, MLM; C, MLMLM; D, CMLM; E, ECMLM; F, Blink; G, FaST; H, EMMA; and I, EMMAx models respectively.

## Data Availability

The Nanopore long reads and Illumina short reads are uploaded to the China National Center for Bioinformation GSA (Genome Sequence Archive) database under BioProject PRJCA006928, and the submission ID is subPRO010171.
