# Supplementary material for: Chromosome‐level wild Hevea brasiliensis genome provides new tools for genomic‐assisted breeding and valuable loci to elevate rubber yield
Source: Plant Biotechnol J. 2023 Feb 22;21(5):1058–72. doi: 10.1111/pbi.14018 (PMC10106855; doi:10.1111/pbi.14018)
Supplement: Supplementary file 2 — Note S1. Reference genome sequencing and assembly. Note S2. Reference genome annotation. Note S3. Comparative genomic analysis. Note S4. Gene family analysis. Note S5. Re‐sequencing and phylogenetic analysis. Figure S1. Heterozygosity estimation survey basing on kmer‐17 analysis. The kmer k‐mer occurrence plot of wild germplasm MT/VB/25A 57/8 indicated a very low heterozygosity rate about 0.462%. Figure S2. Kmer‐17 distribution curve of 50× Sequel WGS data. Figure S3. The ONT reads length distribution. Figure S4. The accumulative length distribution of assembled contigs. Figure S5. Hi‐C interaction heatmap of 18 clusters in Hevea brasiliensis genome. Figure S6. CEGMA assessment results. Figure S7. BUSCO assessment results. Figure S8. Distribution between GC content and sequencing depth. Figure S9. Distribution of divergence rate of each type of transposable element (TE) in the rubber tree genome assembly based on homology‐based prediction using RepeatModeler. Figure S10. Species distribution of NR annotation from the genes of Hevea brasiliensis. Figure S11. KEGG pathway classification of genes in Hevea brasiliensis genome. Figure S12. KOG functional classification of genes in Hevea brasiliensis genome. Figure S13. Gene ontology classification of genes in Hevea brasiliensis genome. Figure S14. Venn diagram of rubber tree genes annotation between NR, GO, KOG, KEGG, Swissprot databases. Figure S15. Cross‐species comparisons in the length distribution of genes, CDSs, exons, introns, and the numbers of exons and introns. Figure S16. Synteny plot of the rubber tree genome. Figure S17. Ks curve of Cassava (A), Rubber tree (B) and Poplar (C). Figure S18. Synteny plot between the rubber tree and M. esculenta genome. Figure S19. Genome collinearity and gene family analysis in rubber tree genome. Figure S21. Evolutionary tree of gene family expansion and contraction. Figure S22. Diagram showing the program for the rubber tree conventional breeding. Figure S23. Statistics of po [file PBI-21-1058-s001.docx]

**A chromosome-level genome sequence of wild rubber tree reveals genetic basis and valuable loci to elevate latex yield**

**Running title:** Rubber tree genome and evolution

Han Cheng^#^*, Xiaoming Song^#^, Yanshi Hu^#^, Tingkai Wu, Qihang Yang, Zewei An, Shuyan Feng, Zhi Deng, Wenguan Wu, Xia Zeng, Min Tu, Xiyin Wang*, Huasun Huang*

1, Rubber Research Institute, Chinese Academy of Tropical Agricultural Science, Haikou, Hainan, PR China.

2, School of Life Sciences/Center for Genomics and Bio-computing, North China University of Science and Technology, Tangshan, Hebei 063210, China;

3, Key Laboratory of Biology and Genetic Resources of Rubber Tree, Ministry of Agriculture, PR China

# Contribute equally to this work

*To whom correspondence should be addressed:

Prof. Cheng Han, Tel: (86)898 -6697-8171

Fax: (86)898-6696-1226

Email: forcheng@gmail.com

Prof. Wang Xiyin, [wangxiyin@vip.sina.com](mailto:wangxiyin@vip.sina.com)

**Supplementary Notes**

[Note 1. Reference genome sequencing and assembly 3](#_Toc74148745)

[S1.1 Plant material 3](#_Toc74148746)

[S1.2 Nanopore sequencing and assembly 3](#_Toc74148747)

[S1.3 Illumina sequencing 4](#_Toc74148748)

[S1.4 HiC library preparation and sequencing and chromosome assembly 5](#_Toc74148749)

[S1.5 Bionano sequencing and scaffolding 6](#_Toc74148750)

[S1.6 Genome evaluation 6](#_Toc74148751)

[Note 2. Reference genome annotation 8](#_Toc74148752)

[S2.1 Genome structure annotation 8](#_Toc74148753)

[S2.2 Functional gene annotation 11](#_Toc74148754)

[S2.3 Annotation evaluation 11](#_Toc74148755)

[Note 3. Comparative genomic analysis 12](#_Toc74148756)

[S3.3 Construction of collinear genes map of rubber and other representative species 13](#_Toc74148757)

[S3.4 The divergence time of rubber and cassava species 13](#_Toc74148758)

[Note 4. Gene family analysis 14](#_Toc74148759)

[S4.1 phylogenetic relationship analysis based on homologous genes 14](#_Toc74148760)

[S4.2 Cluster analysis of gene families 15](#_Toc74148761)

[S4.3 Estimation of species divergence time 15](#_Toc74148762)

[Note 5. Re-sequencing and phylogenetic analysis 16](#_Toc74148763)

[S5.1 Plant material and Illumina sequencing 16](#_Toc74148764)

[S5.2 Phylogenetic analysis 17](#_Toc74148765)

[S5.3 Selective signal analysis 18](#_Toc74148766)

[Supplemental Figures 20](#_Toc74148767)

[Reference 45](#_Toc74148768)

Note 1. Reference genome sequencing and assembly

S1.1 Plant material

We sequenced a wild rubber tree germplasm accession, MT/VB/25A 57/8, which is collected by IRRDB1981 expedition (Othman *et al.*, 2004). This accession was originally collected from Mato Crosso state, Brasil, then deposited in IRRDB wild germplasm repository in Malaysia. After that, this accession was dispatched to the member countries including China, Vietnam, India etc. After a field evaluation, this accession displayed low latex yield when compared with the control clone, RRIM600. This accession also displayed relatively low heterozygosity during the genome survey.

S1.2 Nanopore sequencing and assembly

High molecular weight DNA was extract from new sprout leaves at bronze developmental stage. DNA were extracted with QIAGEN® Genomic Kit and the fragments larger than 20 Kb were selected by BluePippin electrophoresis (Saga Sciences). The recovered DNA fragment was repaired and ligated with SQK- LSK109 ligation kit to construct the Nanopore sequencing library. After purifying, the libraries were loaded onto a flow cell and sequenced on the PromethION platform (Oxford Nanopore Technologies).

The Oxford Nanopore sequencing data were first filtered to remove contaminations, adapters and short sequences. After that, the filtered sequences were correct by the NextCorrect module of Nextdenovo (<https://github.com/Nextomics/NextDenovo>) using the parameters: read_cuoff = 3k，seed_cutoff = 25k，blocksize = 2g. Totally 84.6 Gb consensus nanopore sequences (CNS) were obtained. Then, the smardenovo program was used to assemble the CNS into contigs using the parameters : wtpre -J 3000，wtzmo -k 21 -z 10 -Z 16 -U -1 -m 0.1 -A 1000，wtclp -d 3 -k 300 -m 0.1 -FT，wtlay -w 300 -s 200 -m 0.1 -r 0.95 -c 1 . A Preliminary genome assembly was obtained which have a genome size about 1.70Gb with contig N50 about 3.45Mb. To further improve the accuracy of the assembly, three rounds of consensus correction using Nextpolish program with Nanopore reads and four rounds of consensus correction using BWA v0.7.12 (Li and Durbin, 2009) and Pilon (RRID:SCR 014731) (Walker *et al.*, 2014) with Illumina reads were performed. A polish genome was finally assembled, which have a genome size of 1.72 Gb with Contig N50 3.51 Mb.

S1.3 Illumina sequencing

For Illumina sequencing, high quality genomic DNA for WGS sequencing was extracted from bronze stage leaf tissues using CTAB (Cetyltrimethyl ammonium bromide) method. The 400 bp PE (paired-end) libraries were prepared using according to Illumina protocol. Illumina Sequel sequencing was performed on NovaSeq 6000 platform at Nexgenomic (Wuhan, China). The adapter sequences, leading and trailing bases were filtered using Trimmomatic software (Bolger *et al.*, 2014).

For transcriptome study, RNA was extracted from young leaf, old leaf, young bark, old bark and latex of the rubber tree accession MT/VB/25A 57/8. Illumina cDNA libraries were prepared using reagents from Illumina Truseq RNA Library Preparation kit (Illumina, San Diego, CA USA) according to manufacturer’s instruction. Fragmented RNA served as first-strand for cDNA synthesis with N6 random hexamer primers. The PE adaptor was ligated and the fragments were then selected on an agarose gel and amplified. PE sequencing was performed on an Illumina HiSeq 2500 system (Illumina, San Diego, CA USA).

S1.4 HiC library preparation and sequencing and chromosome assembly

The Hi-C library was prepared followed by a procedure (Belton *et al.*, 2012) with an improved modification. In brief, freshly harvested leaves were cut into 2 cm pieces and vacuum infiltrated in nuclei isolation buffer supplemented with 2% formaldehyde. Crosslinking was stopped by adding glycine and additional vacuum infiltration. Fixed tissue was frozen in liquid nitrogen and grounded to powder before re-suspending in nuclei isolation buffer to obtain a suspension of nuclei. The purified nuclei were digested with 100 units of HindIII and marked by incubating with biotin-14-dCTP. Biotin-14-dCTP from non-ligated DNA ends was removed owing to the exonuclease activity of T4 DNA polymerase. The ligated DNA was sheared into 300−600 bp fragments, and then was blunt-end repaired and A-tailed, followed by purification through biotin-streptavidin-mediated pull down. Finally, the Hi-C libraries were quantified and sequenced using the Illumina Hiseq platform (Illumina, San Diego, CA, USA).

In total, 370 million paired-end reads were generated from the libraries. Then, quality controlling of Hi-C raw data was performed using Hi-C-Pro (v2.8.0) as former research (Burton *et al.*, 2013). Firstly, low-quality sequences (quality scores<20), adaptor sequences and sequences shorter than 30 bp were filtered out using fastp v0.12.6 (RRID:SCR_016962) (Chen *et al.*, 2018), and then the clean paired-end reads were mapped to the draft assembled sequences using bowtie2 v2.3.2 to get the unique mapped paired-end reads (Langmead and Salzberg, 2012).

As a result, 107 million uniquely mapped pair-end reads were generated, of which 76.28% were valid interaction pairs. Combined with the valid Hi-C data, subsequently used the LACHESIS (ligating adjacent chromatin enables scaffolding *in situ*) *de novo* assembly (Burton *et al.*, 2013) pipeline to produce chromosome-level scaffolds.

S1.5 Bionano sequencing and scaffolding

For Bionano optical genome map construction, high molecular weight genomic DNA was extracted with Bionano Prep™ Plant Tissue DNA Isolation Kit (Bionano Genomics, CA, USA), then digested with the single-straned nicking endonuclease DLE-1 and labelled according to BioNano’s standard protocol. The nick-labelled long DNA molecules were then linearized and imaged using the methods based on the NanoChannel Array technology (Irys system, BioNano Genomics). The Bionano raw data were filtered to remove the molecules: length < 150 Kb and MinSites (/100 Kb) < 9. After filtering, 480.80 Gb clean data were obtained with an average label /100 Kb of 19.24 and N50 of 241.5 Kb. Then the Bionano clean data were mapped with assembled genome to obtain Molecule Quality Report (MQR) with a map rate 65.5%. Using the assembled genome as a reference, the Bionano clean data were de novo assembled. This produced a Bionano genome map with total length 1969.90 Mb and N50 46.38 Mb. Then the Nanopore genome was anchored onto Bionano optical map to construct super scaffolds. A final genome was obtained, with a genome size of 1,884.36 Mb, Scaffold N50 47.97 Mb. The total contigs in the final genome is 1723.31 Mb with Contig N50 of 3.23 Mb.

S1.6 Genome evaluation

To determine the accuracy of the final genome version assembly, the Illumina NGS reads described above (50 X coverage) were compared with the reference assembly using bwa 0.7.12-r1039 (Li and Durbin, 2009). Then the SAMtools v1.6 (Li *et al.*, 2009) and Bcftools v1.8.0 were used to count the alignment files and calculate the homozygous and heterozygous mutation sites (Danecek *et al.*, 2021). The homozygous mutation sites were recorded as the genomic error site for the statistics of the single-base error rate of the genome. As a result, the mapping rate of NGS data is 99.82%. SNP and Indel were inspected according to the alignment results. Totally 38,012 homozygous SNPs were found, accounting for 0.002210% of the genome (Depth >=5 X). There were 16,790 homozygous Indels, accounting for 0.000976% of the genome (Depth >= 5X). Therefore, the single-base accuracy of the genome is 99.996814% (Depth >= 5X).

To evaluate the completeness genome of the assembly, we used Benchmarking Universal Single-Copy Orthologs (BUSCO) v3.0.1 (Simão *et al.*, 2015) to search the annotated genes against embryophyte_odb10 in the assembly. As a result, 97.75% complete BUSCOs (C) were found (Supplemental Table S7) indicating the high integrity of the assembled genome. Core Eukaryotic Genes Mapping Approach (CEGMA) core genes dataset (Parra *et al.*, 2007) was used to evaluate the genome accuracy and integrity by predicting core genes in the assembled genome. Totally 244 core genes were found (98.39% completeness), in which 233 genes were complete genes (an alignment length >= 70% of the protein length).

To evaluate the contamination in the assembled genome, GC depth analysis was conducted. First, minimap2 was used to compare the assembled genome with the Nanopore reads to obtain the aligned bam file, and then use SAMtools to calculate the sequencing depth of each site in the assembled genome based on the alignment results. Totally 99.70% of the Nanopore reads were mapped onto the assembled genome. Then GC content and average sequencing depth were calculated using a 10Kb sliding window of each segment of each contigs/scaffolds sequence. Finally, a distribution map of average GC content and average sequencing depth was constructed. GC content was distributed between 30-40% while the sequencing depth was concentrated between 70-90 X. Due to the specificity of the GC content between species, single the GC depth concentration area means that there is no obvious contamination in the assembled genome.

To further count the possible contamination sources in the assembled genome, an alignment NT (Nucleotide Sequence Database) was conducted. First, the genome sequences were spited into 50 Kb bins to form a segmented sequence file. Then the segmented file was aligned against NT database, and bin that have maximum matches were counted as the results of this sequence. Finally, the assembled genome was aligned against adapters database to find any possible adapter sequence in the genome. The results turned out the about 86.46% sequences were mapped with Viridiplantae and about 0.12% of the total genome was mapped with mitochondrion or chloroplast. No other contamination source was found, indicating the assembled genome contains no obvious contamination.

Note 2. Reference genome annotation

S2.1 Genome structure annotation

**S2.1.1 Repetitive sequences prediction**

The tandem repeats were first annotated using the software GMATA (Wang and Wang, 2016) and Tandem Repeats Finder (TRF) (Benson, 1999) where GMATA identifies the simple repeat sequences (SSRs) and TRF recognizes all tandem repeat elements in the whole genome. Totally 75,621 SSR sequences were identified, with a total length of 1,062,994 bp (Table S12). For the TR, the total number is 75,416 and the length is 5,040,329 bp, occupying 0.27% of the total genome length (Table S12). Transposable elements (TE) in the rubber tree genome were then identified by the combination of *ab inito* and homology-based methods. In short, an *ab inito* repeat library for rubber tree was first predicted using MITE-hunter (Han and Wessler, 2010) and RepeatModeler (Flynn *et al.*, 2020) with default parameters, in which LTR_FINDER, ltr_harverst and LTR_retriver were also be included for plant genome. The obtained library was then aligned to TEclass Repbase (http://www.girinst.org/repbase) to classify the types of each repeat family. To further identify the repeats at genome wide, RepeatMasker (Tarailo-Graovac and Chen, 2009) was used to search for known and novel TEs against the *de novo* repeat library and Repbase TE library. Overlapping TEs belonging to the same repeat class were collated and combined.

For the transposable element (TE) identification, the MITE-hunter software was used to search for the miniature inverted-repeat transposable elements (MITE) in the TR softmasked genome with the parameters: -n 20 -P 0.2 -c 3. Then the LTR_FINDER (Xu Z et al. 2007) and ltr_harverst (D. Ellinghaus et al. 2008) were used to search the repetitive sequences and to construct an LTR repetitive sequence library with LTR_retriver (Ou and Jiang, 2018) program. The LTR and MITE libraries were combined to form the TE library file (TE.lib) and the genome sequence was hard masked with this TE library to replace the repetitive sequences with N. After that, an *ab inito* search for the repetitive sequences was conducted with the Repeat Modeler 1.0.11 (Flynn *et al.*, 2020) software and an *ab inito* library (RepMod.lib) was obtained. The obtained library was then aligned to TEclass Repbase (http://www.girinst.org/repbase) to classify the types of each repeat family. The unknown repetitive sequence in the RepMod.lib were further classified using TEclass (Abrusán *et al.*, 2009) program. Finally, the TE.lib, RepMod.lib and Repbase (Jurka *et al.*, 2005) libraries were integrated into one library and using this library to search the genome sequences with RepeatMasker software v1.331 (Tarailo-Graovac and Chen, 2009) using parameters: nolow -no_is -gff -norna -engine abblast -lib lib. As a result, the total length of the TE sequences in the rubber tree genome is 1,423,596,238 bp, occupying 75.55% of total genome length. The LTR is the most abundant TE in the rubber tree genome, with a total percentage about 71.05% of the total genome length. The TE, TR and other repetitive sequences in the rubber tree genome are 1,445,635,952 bp in length, with a percentage about 76.72% of total genome (Table S12).

**S2.1.2 Gene prediction**

The structural genes were predicted by three strategies: ab initio prediction, homology search and reference guided transcriptome assembly. For ab initio prediction, AUGUSTUS v3.3.1 (Stanke *et al.*, 2004) was used with the parameters: --gff3=on --hintsfile=hints.gff --extrinsicCfgFile=extrinsic.cfg --allow_hinted_splicesites=gcag,atac --min_intron_len=30 --softmasking=1. The RNA-seq data was used as training dataset with GeneMark-ET software (Lomsadze *et al.*, 2014) using the parameters: --max_intron max_intron --soft_mask soft_length --pbs --sequence=genome --ET=introns.gff. Totally 253,852 gene models were obtained. The top reliable 2,000 gene models were further used as training dataset for AUGUSTUS to predict the structural genes. As a result, 39,251 genes were predicted by AUGUSTUS (Table S13).

For homology search, GeMoMa v1.6.1 (Keilwagen *et al.*, 2019) was used to predict the rubber tree genes with coding genes from relative species: *Jatropha curcas, Ricinus communis, Manihot esculenta and Populus trichocarpa*. Totally 44,063 genes were predicted by GeMoMa (Table S13).

For reference guided transcriptome assembly, the clean NGS transcriptomic data were mapped with genome sequences using STAR v2.7.3a software(Dobin *et al.*, 2013), then the mapping location file was obtained using Stringtie v1.3.4d (Pertea *et al.*, 2016). The corresponding sequences at the mapping locations were extracted. Finally, the PASA (Program to Assemble Spliced Alignments) v2.3.3 (Haas *et al.*, 2003) was used to construct gene models basing on the alternative splicing information. GMAP (Wu and Watanabe, 2005) was further used to mapping the gene models with genome sequence. Finally, the GeneMarkST v5.1 (Tang *et al.*, 2015) was used to find the suitable ORF (open reading frame) in the genome. The final genes predicted with PASA was 30,262 (Table S13).

The EVidenceModeler (aka EVM) v1.1.1 software (Haas *et al.*, 2008) was used to integrate the prediction results from the above strategies. A non-redundant exon dataset was obtained and used to define the most reliable rubber tree genes. The combined gene number was 35,318 (Table S13).

The TransposonPSI v08222010 software (http://transposonpsi.sourceforge.net/) was used to recognize the pseudogenes that possibly integrated in the genome. All the genes that mapped with the setting database were removed. The genes with a length that was not multiple of three, or with a stop codon in the middle of the sequence were also removed. Untranslated regions (UTRs) and alternative splicing regions were determined using PASA based on RNA-seq assemblies. The longest transcripts were retained for each locus, and regions outside of the ORFs were designated UTRs. Finally, 35,318 genes were predicted from the rubber tree genome sequences, with an average gene length 4,286.33 bp (Table S13). This final dataset was used for the next analysis.

**2.1.3 Non-coding RNA prediction**

For non-coding RNA prediction, three methods were used. First, cmscan program in the Infernal v1.1.2 software (Nawrocki and Eddy, 2013) was used to align the genome sequences with Rfam database (Griffiths-Jones *et al.*, 2005) to detect MicroRNA, rRNA, small nuclear RNA (snRNA), and small nucleolar RNA (snoRNA). Second, tRNAscan-SE v2.0 (Chan and Lowe, 2019) was adopted to search tRNA in the rubber tree genome with eukaryote parameters. Third, rRNAs and their subunit were predicted with the models constructed by RNAmmer v1.2 (Lagesen *et al.*, 2007). The results of these three methods were integrated, and finally 449 rRNAs, 923 tRNAs and 4,955 small RNAs were found (Table S15).

S2.2 Functional gene annotation

The predicted genes were further annotated by aligning the corresponding translated proteins sequence agains NR (Non-Reduntant Protein Database), KEGG (Kyoto Encyclopedia of Gene and Genomes) (Moriya *et al.*, 2007), KOG (Eukaryotic Orthologous Groups of protein) (Galperin *et al.*, 2015), GO (Gene Ontology) (Ashburner *et al.*, 2000) and Swissport databases (Stanke and Waack, 2003) with blastp program: -evalue 1e-5，-max_target_seqs 1. The annotation results were as shown in Table S16. After a combining of all the annotation results, overall, 38,866 genes were successfully annotated, occupying 98.79% of the predicted genes (Table S16).

S2.3 Annotation evaluation

Benchmarking Universal Single-Copy Orthologs (BUSCO) was used for genome annotation evaluation. The annotated protein sequences were aligned with corresponding BUSCO databases with HMMER3 software, and the BUSCOs were counted according to the integrity of the aligning results. As a result, 1,336 complete BUSCOs were found and accounted for about 97.16% of total BUSCO groups, indicating that most of the conserved genes were properly assembled and annotated (Table S7).

Note 3. Comparative genomic analysis

**S3.1 Whole genome sequence collection**

The genome sequences of grape (*Vitis vinifera*), poplar (*Populus* *trichocarpa*) and cassava (*Manihot* *esculenta*) were downloaded from plant comparative genomics platform phytozome database (https://phytozome.jgi.doe.gov/pz/portal.html). The genome sequence of rubber (*Hevea* *brasiliensis*) was downloaded from the NCBI (<https://www.ncbi.nlm.nih.gov/>) (Tang *et al.*, 2016).

**S3.2 Homology genomic structure analysis between rubber tree and other species**

Based on the homology comparison results of the genome sequence and the physical or relative position of each gene on the chromosome, a lattice diagram of structural homology within and between the genome of each species is constructed. The structural characteristics of the genome were displayed by the homology dot plot, and speculate on the genome-wide duplication event of the species at the genome level. This process is mainly based on the Perl script, using internal programs written to read BLAST formatted output files, and generate structural homology bitmaps. Combining the homology analysis of the genome structure, distinguish the repeated genes in the genome and between the genomes generated by different duplication events, and integrate the results to generate a joint comparison map of the homologous gene pairs of the two species.

##### S3.3 Construction of collinear genes map of rubber and other representative species

Gene collinearity is the key to understanding genome evolution and can be used for evolutionary analysis of complex genomes. We analyzed the collinearity within and between the grape, rubber and cassava genomes and obtained corresponding homology information.

The number of collinearity fragments can reflect the loss of genomic homologous fragments and genetic relationship. We have detailed statistics of homologous collinearity fragments and gene pairs within and between the grape, rubber, and cassava genomes (Table S17). In the homology relationship between rubber and cassava, when the length of the homologous collinearity fragment is greater than 4, there are 1,827 homologous fragments and 56,191 homologous gene pairs; when the length of the homologous collinearity fragment is greater than 50, there are still 143 homologous fragments and 36,170 homologous gene pairs. These homologous fragments were further divided into sub-genomes to show the evolutionary relationship between rubber and cassava.

S3.4 The divergence time of rubber and cassava species

In the genomes of rubber and cassava, the duplicated genes produced by the whole genome duplication event of their common ancestors are retained. These repeated genes provide a data basis for inferring the main evolutionary events that occurred during the evolution of rubber and cassava. Using repeated genes within and between genomes of each species, we plotted the distribution curve of Ks values (Figure S17). Cassava has experienced two duplication events, and its Ks peaks were 0.25 and 1.5, respectively (Figure S17A). Rubber also experienced two duplication events, and its Ks peaks were 0.3 and 1.5, respectively (Figure S17B). Poplar has also experienced two duplication events, and its Ks peaks are 0.25 and 1.5, respectively (Figure S17C). Cassava, rubber, and poplar have experienced an older duplication together, while the recent duplication of rubber and cassava cannot be accurately judged because the Ks value is too close. Timetree is used to estimate the divergence time of rubber and cassava (<http://www.timetree.org/>) (Kumar *et al.*, 2017). The results show that the divergence time is between 12.89 million years and 20.07 million years.

Note 4. Gene family analysis

S4.1 phylogenetic relationship analysis based on homologous genes

Using the poplar genome as a reference, 1,051 common homologous gene pairs were identified from the collinearity list of poplar and cassava, poplar and rubber. The phylogenetic tree of 1,051 homologous genes was constructed in batches by the NJ method of MEGACC (<https://www.megasoftware.net>) (Tamura *et al.*, 2013), and then all the phylogenetic trees obtained were statistically analyzed.

Among them, there are 1,024 phylogenetic trees with type of the Figure S20A, accounting for 68.17% of the total phylogenetic trees. There are 17 phylogenetic trees with type of Figure S20B, accounting for 1.13% of the total phylogenetic trees. There are 45 phylogenetic trees with type of Figure S20C, accounting for 2.29% of the total phylogenetic trees. There are 100 phylogenetic trees with type of Figure S20D, accounting for 6.66% of the total phylogenetic trees.

Our research focuses on statistical analysis of A-type and B-type phylogenetic trees. Using poplar as the outgroup, we constructed the gene trees using the most recent homologous gene sequence of rubber and cassava. The A-type evolutionary tree divides the two sub-gene components produced by duplication into two branches. The evolutionary tree shows that the genetic relationship between two sub-genomes of the rubber or the cassava is greater than the genetic relationship between the respective sub-genomes of rubber and cassava. The B-type evolutionary tree separates the two sub-genomes of rubber and cassava. The evolutionary tree shows that the genetic relationship between the respective sub-genomes of rubber cassava is greater than the genetic relationship between the two sub-genomes of the rubber and the cassava.

Type A phylogenetic trees accounted for 68.17% of the total, while Type B phylogenetic trees accounted for only 1.13%. And all other phylogenetic trees accounted for less than 10%. From this we infer that rubber and cassava have undergone a duplication event together instead of experiencing a duplication event after the differentiation of rubber and cassava. It is very likely that this duplication event led to the accelerated differentiation of rubber and cassava.

S4.2 Cluster analysis of gene families

In addition to the gene family analysis of 9 species, we further extracted the gene family clustering results of rubber tree (Hbr), jatropha (Jcu), cassava (Mes), castor (Rco) and poplar (Ptr). Then, we draw a Venn diagram using R language to show the common and specific gene families among these 5 species (Figure S21). Compared with the other four species, the results showed that there were 403 rubber-specific gene families and 13,142 gene families shared by the five species. These unique genes of rubber can be used to explore the relationship between unique genes and special biological characteristics of rubber, laying the foundation for the research of rubber functional genomics.

S4.3 Estimation of species divergence time

The 75 single-copy gene families identified from rubber and other 8 species in this study were used to construct a phylogenetic tree. Then, we calculated the divergence time of species in the phylogenetic tree using the mcmctree program of the PAML software (Figure S19D) (Yang, 2007). The correction time nodes used were list as follows, rubber (Hbr) and cassava (Mes) (16.74Mya), poplar (Ptr) and small willow (Sbr) (16.87Mys), rubber (Hbr) and Jatropha (Jcu) (44.72) Mya), rubber (Hbr) and poplar (Ptr) (75.55Mya), rubber (Hbr) and flax (Lus) (89.69Mya), rubber (Hbr) and castor (Rco) (93.73Mya), rubber (Hbr) and Arabidopsis (Ath) (108.85Mya), rubber (Hbr) and grape (Vvi) (120.88Mya). After recalculation and correction, the divergence time between rubber and cassava is about 16.74 million years, which is consistent with the previous estimation on the Timetree website of 12.89 to 20.07 Mya.

S4.4 Genome duplication and gene collinearity in rubber tree genome

Gene collinearity analysis revealed that the rubber tree genome experienced a whole-genome triplication (WGT) event (Figure S16), which was shared by major dicots (Jaillon *et al.*, 2007). Homologous gene dot plots indicated that 6 groups of homologous chromosomal fragments were present in the rubber tree genome. This phenomenon indicated that the rubber genome had experienced a whole-genome duplication (WGD) event (Figure S17).

We also compared gene collinearity between the rubber tree and cassava genomes. The homologous gene dot plots between the genomes (Figure S18) revealed that each of the rubber tree chromosomes shared two overlapping homologous segments in the cassava genomes. This suggests the occurrence of the genome duplication event prior to the event of species differentiation, as further supported by the phylogenetic analysis shown below..

Gene collinearity within and between the grape, rubber, and cassava genomes was analyzed to show paralogous or orthologous regions (Table S18). Actually, a multi-species genome homology comparison was conducted using the grape genome as a reference to characterize likely gene deletion (Figure S19A). Surprisingly, a large number of homologous genes were likely deleted from the corresponding homologous regions. This can be potentially attributed to the loss of repeated genes during the process of species differentiation. In addition, the absence of these homologous genes was observed in the corresponding cassava collinear regions, as indicated by the collinearity map. These results demonstrated a segregated karyotype change in rubber trees and cassava following the process of WGD.

The genome structure of poplar preserves a genome structure similar to those of rubber trees and cassava. We therefore constructed a further multi-species genome homology comparison using the poplar genome as the reference (Figure 19B). The homologous collinearity between the genomes showed that the rubber tree had gone through numerous inter-chromosomal recombination events following the differentiation from cassava, and poplar. The number of homologous gene pairs retained by rubber tree and poplar was higher than the number of homologous gene pairs retained by grapes. These results indicated that the genome structures of the rubber tree and cassava were much more similar than with grape. The results also indicated that the changes in gene content and inter-chromosomal recombination may have contributed to species differentiation.

A phylogenetic analysis of homologous genes helps perform a relative dating of the whole-genome duplication. A total of 1,051 quartets of homologous genes, two duplicated copies from each genome, were identified in collinear regions between poplar and cassava (or rubber tree). Phylogenetic trees were constructed for homologous gene quartets. We considered three types of gene trees, with Type A to show a common whole-genome duplication shared by the two plants, and Type B to show independent occurrence of whole-genome duplications. The results showed that the Type A phylogenetic trees accounted for 68.17% of the total homologous gene pairs, the Type B phylogenetic trees accounted for only 1.13%, and all other phylogenetic trees accounted for less than 10% (Figure S20). This provides clear evidence that rubber and cassava have undergone a common whole-genome duplication event.

Gene family clustering and expansion

Nine species were used to perform gene family clustering analysis, and a total of 26,763 gene families were identified (Table S19). Seventy-five single-copy gene families were shared by each species (Figure 19C). In the rubber tree genome, 403 gene families were found to be rubber tree specific, while 13,142 families were shared by other species (Figure 19D).

The processes of gene family expansion and contraction were analyzed (Figure S21). The rubber tree has seen gene expansion in 3,060 gene families and contraction in 6,155 families. Especially, 1,331 gene families changed significantly in the rubber tree (*p* < 0.01).

The divergence time was calculated by constructing a phylogenetic tree with 75 single-copy gene families (Table S20). The divergence time between rubber and cassava was found to be approximately 16.74 million years (Figure 19D).

Note 5. Re-sequencing and phylogenetic analysis

S5.1 Plant material and Illumina sequencing

A total of 147 accessions from Asia, south America and Africa were used for re-sequencing (Table S20). This includes three types of accession: (1) 107 Wickham accessions representing recently domesticated clones from 11 countries; (2) 34 IRRBD1981’ wild accessions which is collected in Brazil (AC, Acre; RO, Rondônia; MT, Mato Grosso); (3) five relative species (*H. camargoana*, *H. nitida*, *H. pauciflora*, *H. spruceana*, *H. microphylla*).

For Illumina resequencing, high genomic DNA was extracted from leaf tissue using CTAB method and 150-bp paired-end (PE) libraries were prepared using NEBNex Ultra DNA Library Prep Kit for Illumina. The population resequencing was conducted at Biomarker, China. The sequencing was performed according to Illumina standard protocol on a NovaSeq 6000 platform. The raw reads were trimmed through the NGS QC Toolkit v2.3.3 (Patel and Jain, 2012), and filtered to get rid of the reads with adapters, Phred score <10, or N >10%. The clean reads were then mapped to the compiled reference genome with BWA software (Li and Durbin, 2009), and the statistics was then counted. The SNP was then called using Genome Analysis tool kit (GATK) with HaplotypeCaller and GenotypeGVCFs (McKenna *et al.*, 2010). For single-sample SNP and genotype calling, several filtering steps were performed using GATK filters (QUAL/DP < 2.0 || FS > 60.0 || MQ < 40.0 || MQRankSum < -12.5 || ReadPosRankSum < -8.0) to remove: (1) indels with a quality scores <30, (2) SNPs with more than two alleles, (3) SNPs at or within 5 bp from any indels, (4) SNPs with a genotyping quality scores (GQ) <10, and (5) SNPs with extremely low (< one-third average depth) or extremely high (>threefold average depth) coverage. The obtained SNPs were finally annotated with SnpEff v4.3t (Cingolani *et al.*, 2012).

S5.2 Phylogenetic analysis

Totally 18.7 billion high quality reads from 147 rubber tree accessions (Table S21) were obtained and mapped on the wild rubber tree genome, leading to the identification of 84.05 million SNPs and InDels (Figure S24, Table S22). About 70% of the variants were detected in the intergenic regions followed by 5’ UTR, 3’ UTR, introns and CDS (Table S23).

For phylogenetic analysis, the VCF files were filtered to remove indels, non-biallelic SNPs and the SNP with missing rate >90%. Population structure was analyzed using the maximum-likelihood approach implemented in ADMIXTURE v1.3.0 (Dh *et al.*, 2009). Individual-based clustering analysis was conducted and cross-validated to explore convergence and determine the optimum number of clusters (K, from 2 to 10, with the optimum K value indicated by lowest cross-validation error). The results indicated that K=4 produces the lowest CV errors (Figure S27, Table S25) followed by K2. We therefore deployed K=2 to 4 to display the population structure (Figure 3C), in which the three types of accession could not be distinctly grouped.

For neighbor-joining phylogenetic tree construction, SNPs was selected using PLINK2 v2.00a3LM, then Tamura-Nei model in Molecular Evolutionary Genetics Analysis Compute Core (MEGA-CC) 10.1.8 software (Kumar *et al.*, 2012) was used to construct the tree with 1,000 replicates of bootstrap. The generated newick tree was imported into the iTOL (Interactive tree of life) online tool (https://itol.embl.de/) to display the neighbor-joining tree. The results were shown in Figure 3A, most of the domesticated Wickham clones were phylogenetically clustered, while the RO and AC accession are closer than MT accessions.

For PCA (Principal Component Analysis) analysis, the VCF file was first converted to PED and MAP format files with plink. Then these files were converted into the input files (.snp, .ind, and .eigenstratgeno) for the smartpca program with the convertf program in EIGNESOFT v6.1.4 software (Price *et al.*, 2006). Finally, the smartpca program was used for PC calculation with the parameters: altnormstyle: NO; numoutevec: 20; numoutlieriter: 5; outliersigmathresh: 6.0. The results were displayed with tidyverse 1.3.1 package in R4.0.3 (Figure 3B, Figure S26).

For linkage disequilibrium analysis, PopLDdecay v3.41(Zhang *et al.*, 2019) was used. Due to the number of cultivar clones is much more than IRRDB1981 accessions, we first randomly selected 34 accessions from cultivar clones with the bash *shuf* command. Then the population stat was calculated for the selected cultivar clones and wild accessions respectively. The results were finally plotted with Plot_MultiPop.pl scripts in the PopLDdecay package. We did not calculate the LD decay in six relative species due to the limited number.

S5.3 Selective signal analysis

Population fixation statistics (F_ST_) and nucleotide diversity (θ_π_) were calculated using 100 Kb sliding windows in 10 Kb steps for each subgroup dataset by VCFtools v0.1.16 (Danecek *et al.*, 2011). F_ST_ was used to evaluate the tolerance of genomic differentiation on candidates between two subgroups by calculating the genomic differentiation. θ_π_ was used to estimate the genomic diversity of these varieties in each subgroup. The θ_π_ ratios were then calculated between two subgroups for each sliding window. Both the F_ST_ and θ_π_ ratios were used to detect putative selection targets by designating the top 5% of log-odds ratios for both θ_π_ ratios and F_ST_ (Figure S28 - S29). We compared the Wickham clones and wild germplasms and thus obtained the loci that undergo selective sweep during domestication. Totally 363 genomic loci were under positive selection (FST > 0.104; π wild/π Wickham >1.143) (Table S26). The annotated genes in the genomic regions under selection were scanned and 255 genes were identified (Table S27). These genes were used as input list for GO enrichment analysis for the BinGO 3.03 plugin tool in Cytoscape 3.51 (Maere *et al.*, 2005) with GO_full categories. The whole GO term annotation of the genome was used as reference. Overrepresented GO categories were enriched using a hypergeometric test with a significance threshold of 0.05 after a *Benjamini* and *Hocheberg* FDR correction (Benjamini and Hochberg, 1995). The GO enrichment results were further visualized with Cytoscape (Figure 4B).

Supplemental Figures


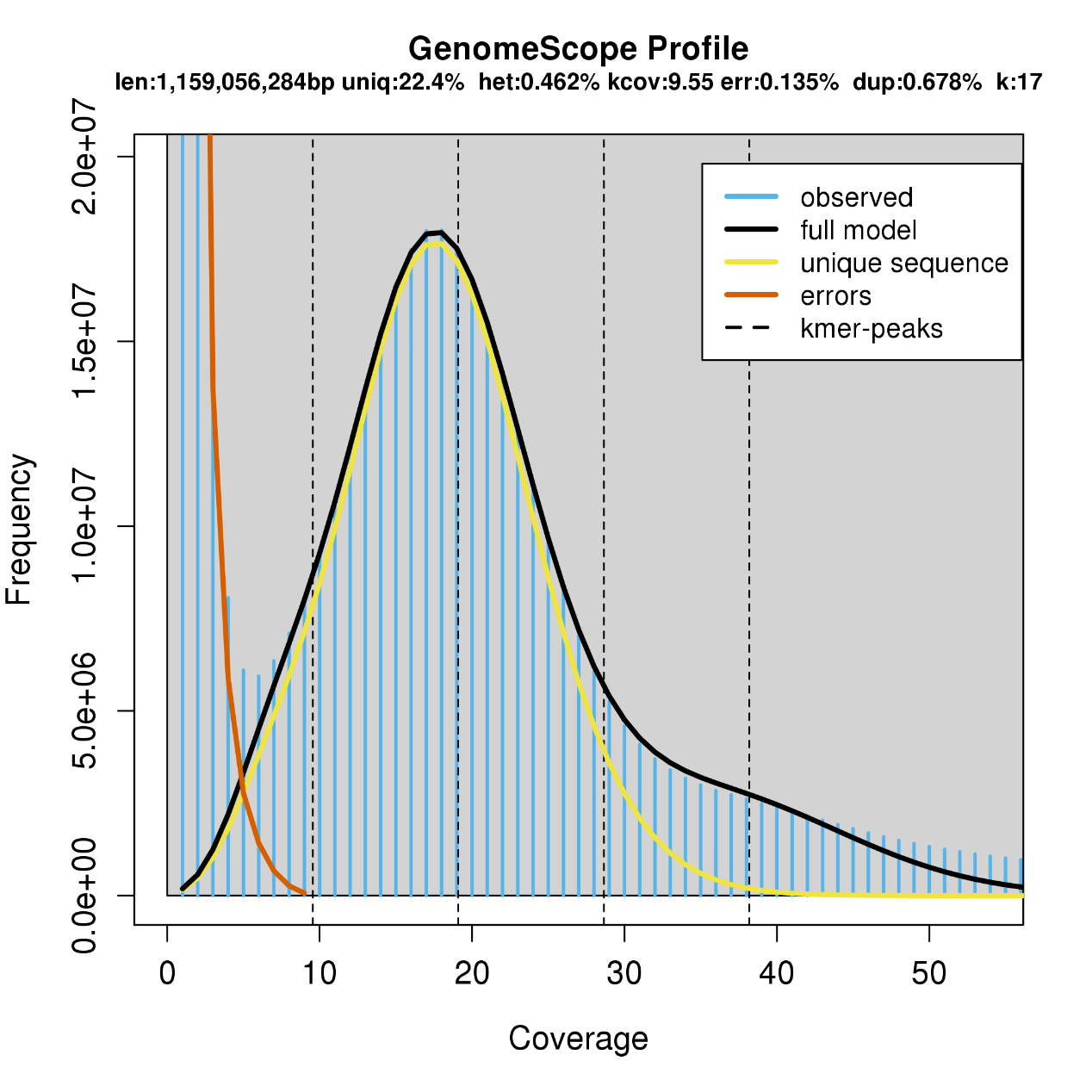


Figure S1. Heterozygosity estimation survey basing on kmer-17 analysis. The kmer k-mer occurrence plot of wild germplasm MT/VB/25A 57/8 indicated a very low heterozygosity rate about 0.462%.


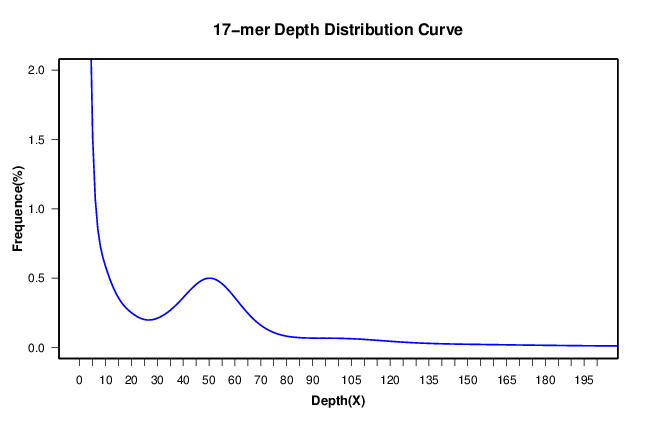


Figure S2. Kmer-17 distribution curve of 50× Sequel WGS data. The total kmer-17 number is 91,198,445,359, and the genome size is estimated to be 1,823,968,907.


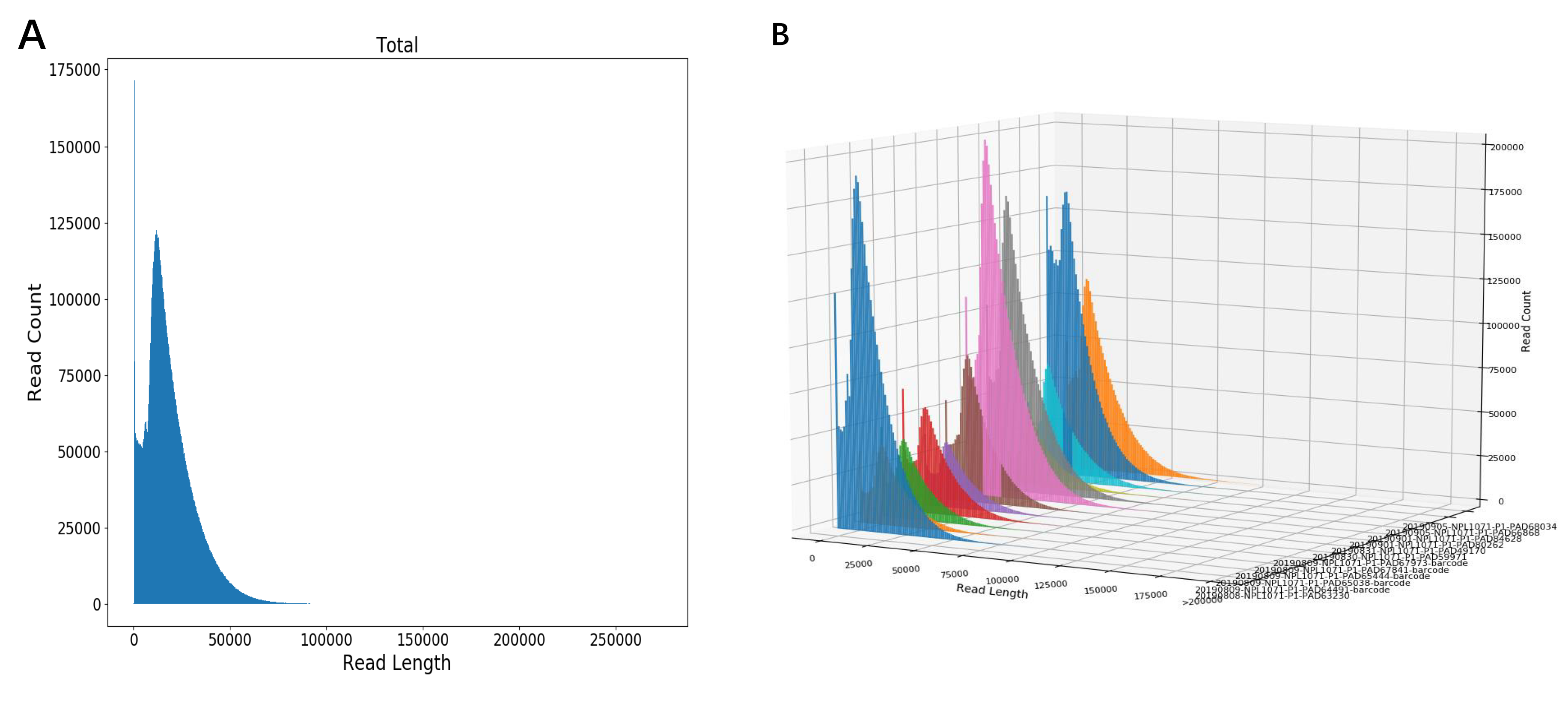


Figure S3. The ONT reads length distribution, A, Total reads; B, Reads from each library. Totally 26,702,320 readsd (499.45Gb) were obtained with an average length about 18.70 Kb and N50 about 24.63 Kb. The longest read is about 273.40 Kb.


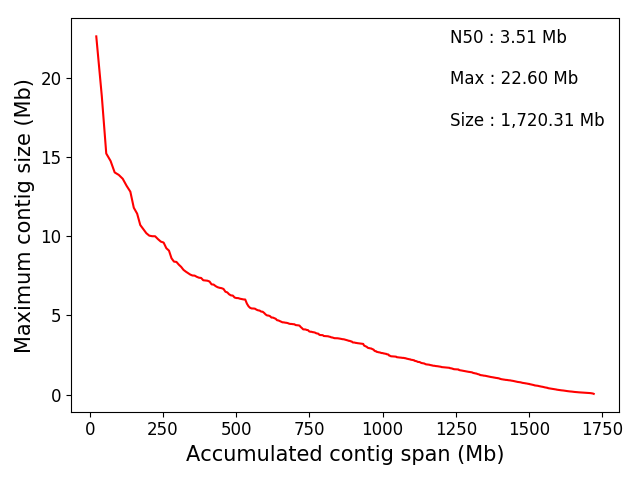


Figure S4. The accumulative length distribution of assembled contigs.


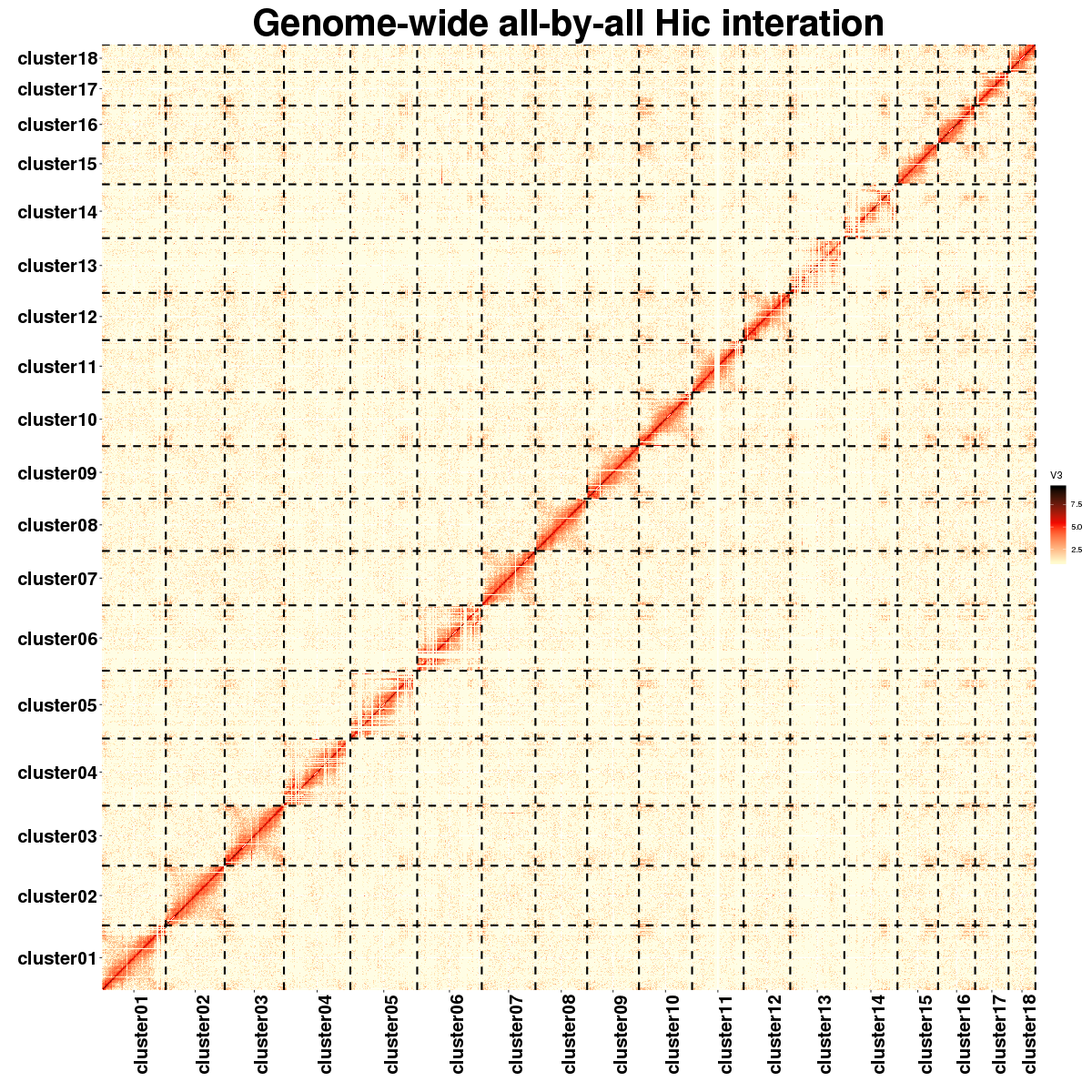


Figure S5. Hi-C interaction heatmap of 18 clusters in *Hevea brasiliensis* genome.


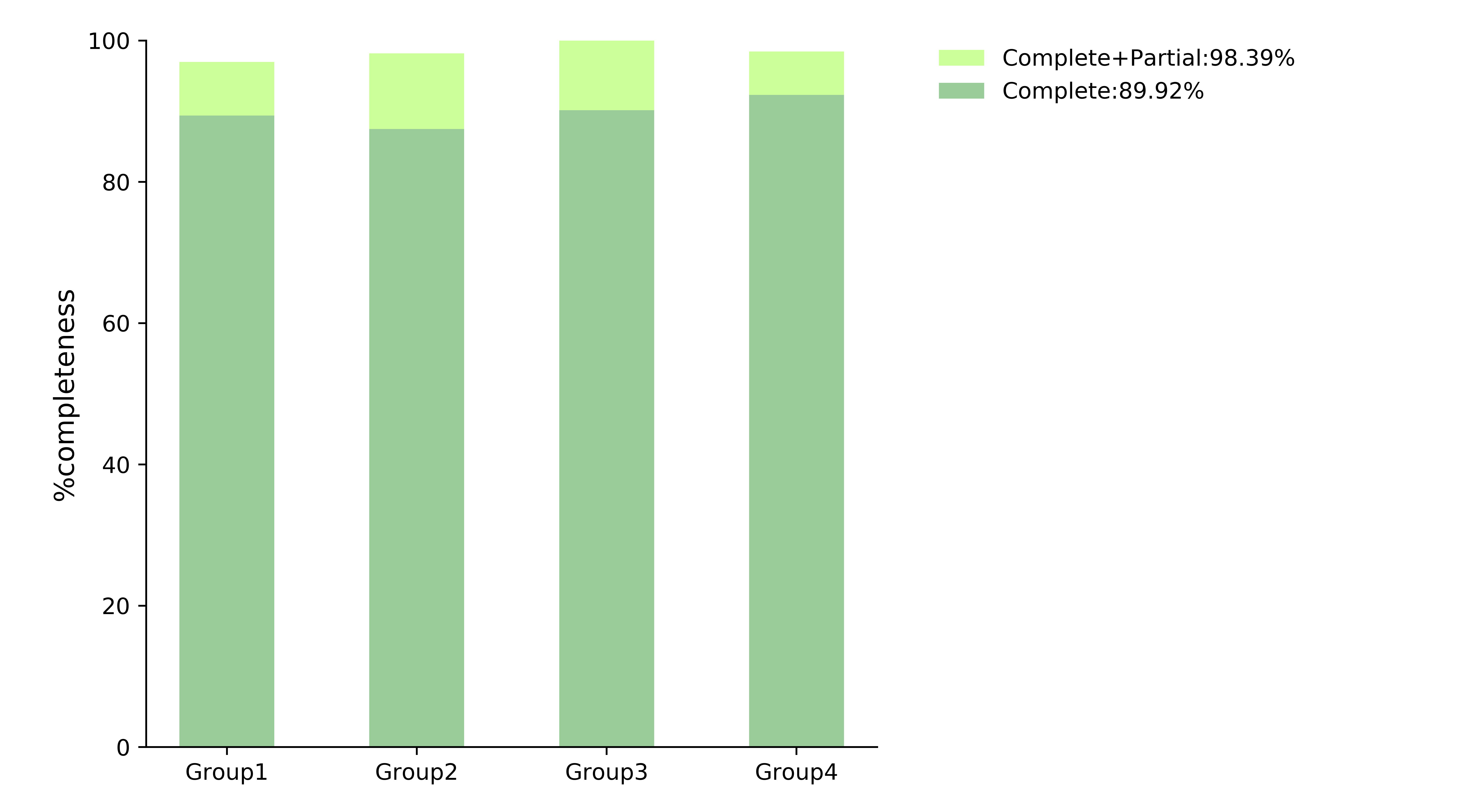


Figure S6. CEGMA assessment results


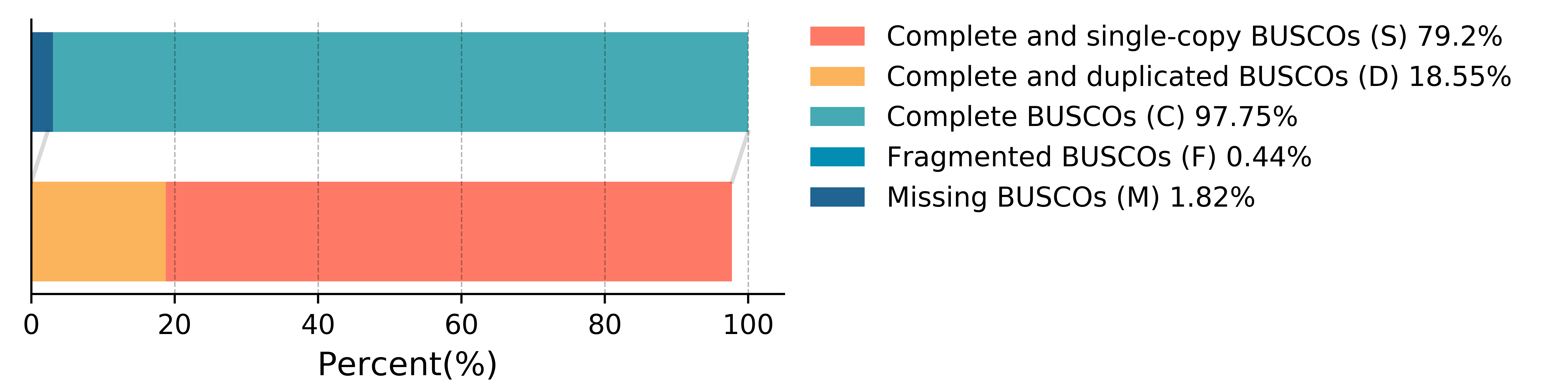


Figure S7. BUSCO assessment results


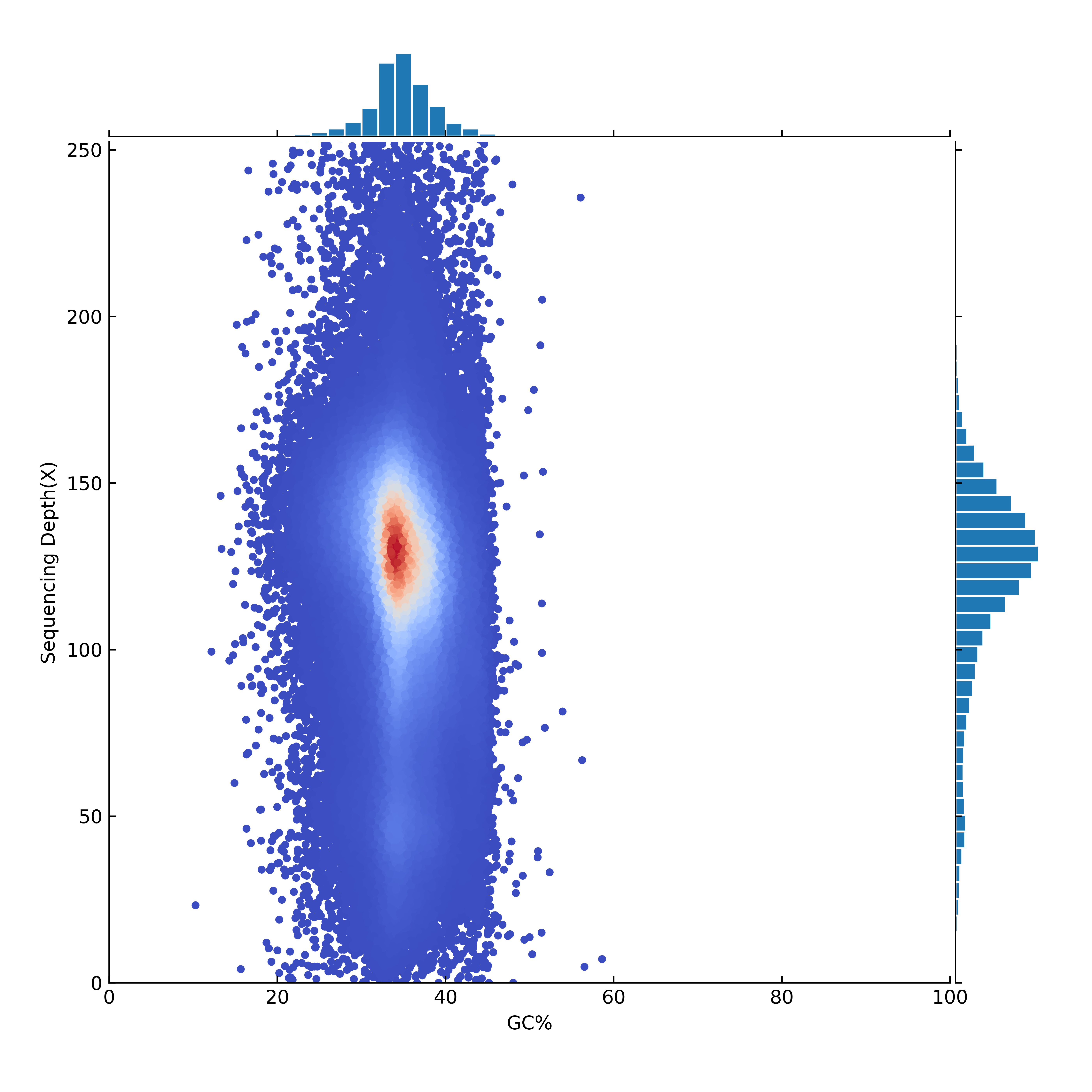


Figure S8. Distribution between GC content and sequencing depth. The GC content and sequence depth were calculated with a 10 kb slide window. Only one concentrated distribution was found in the scatterplot, indicating the sequences were from 1 species without contaminations.


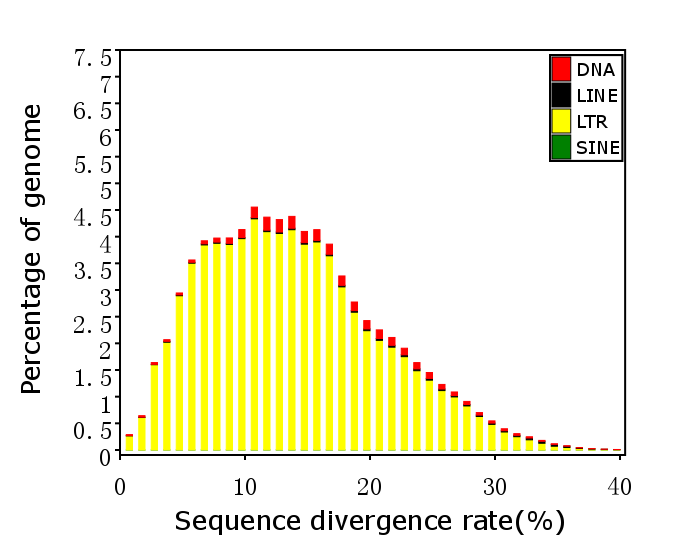


Figure S9. Distribution of divergence rate of each type of transposable element (TE) in the rubber tree genome assembly based on homology-based prediction using RepeatModeler. The divergence rate was calculated between the identified TEs in the genome using a homology-based method and the consensus sequence in the Repbase database. The x-axis represents the sequence divergence rate of repeats. The y-axis represents the percentage of repeat sequences in the genome.


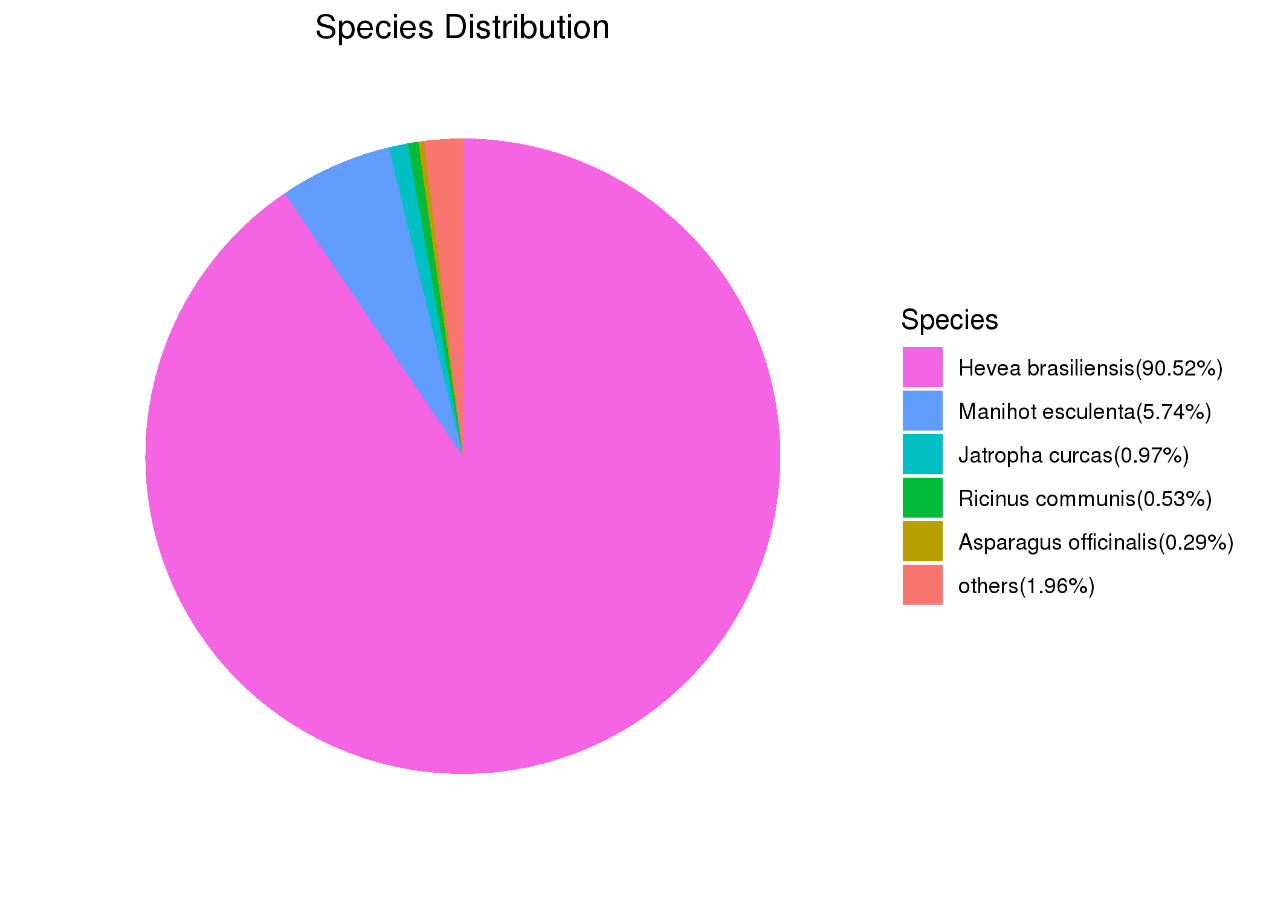


Figure S10. Species distribution of NR annotation from the genes of *Hevea brasiliensis*. A Pie chart showed the homology of unigenes from *Hevea brasiliensis* with that from other species.


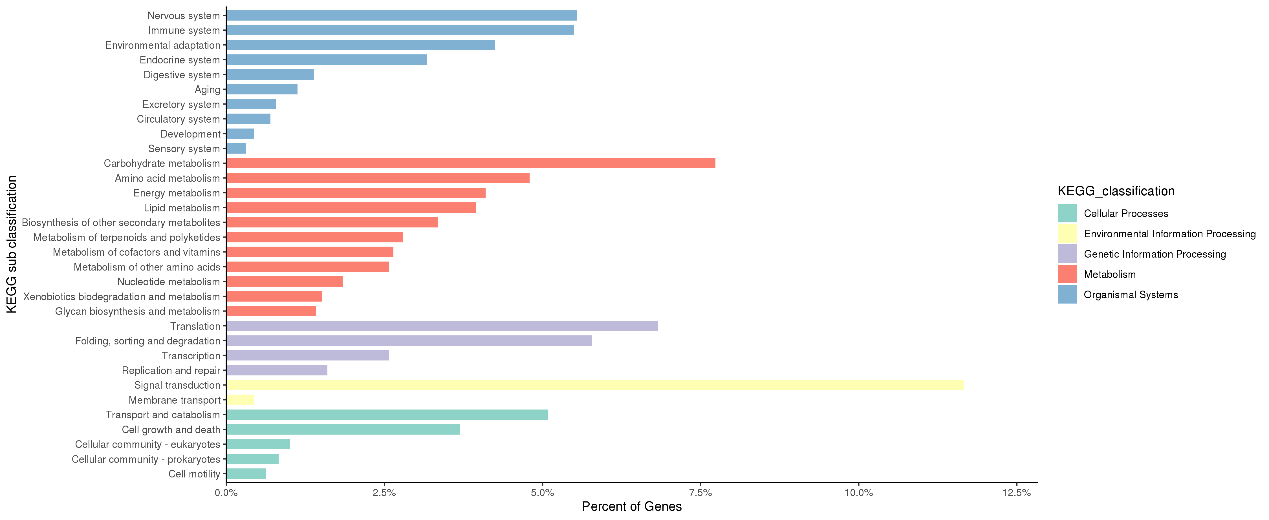


Figure S11. KEGG pathway classification of genes in *Hevea brasiliensis* genome.


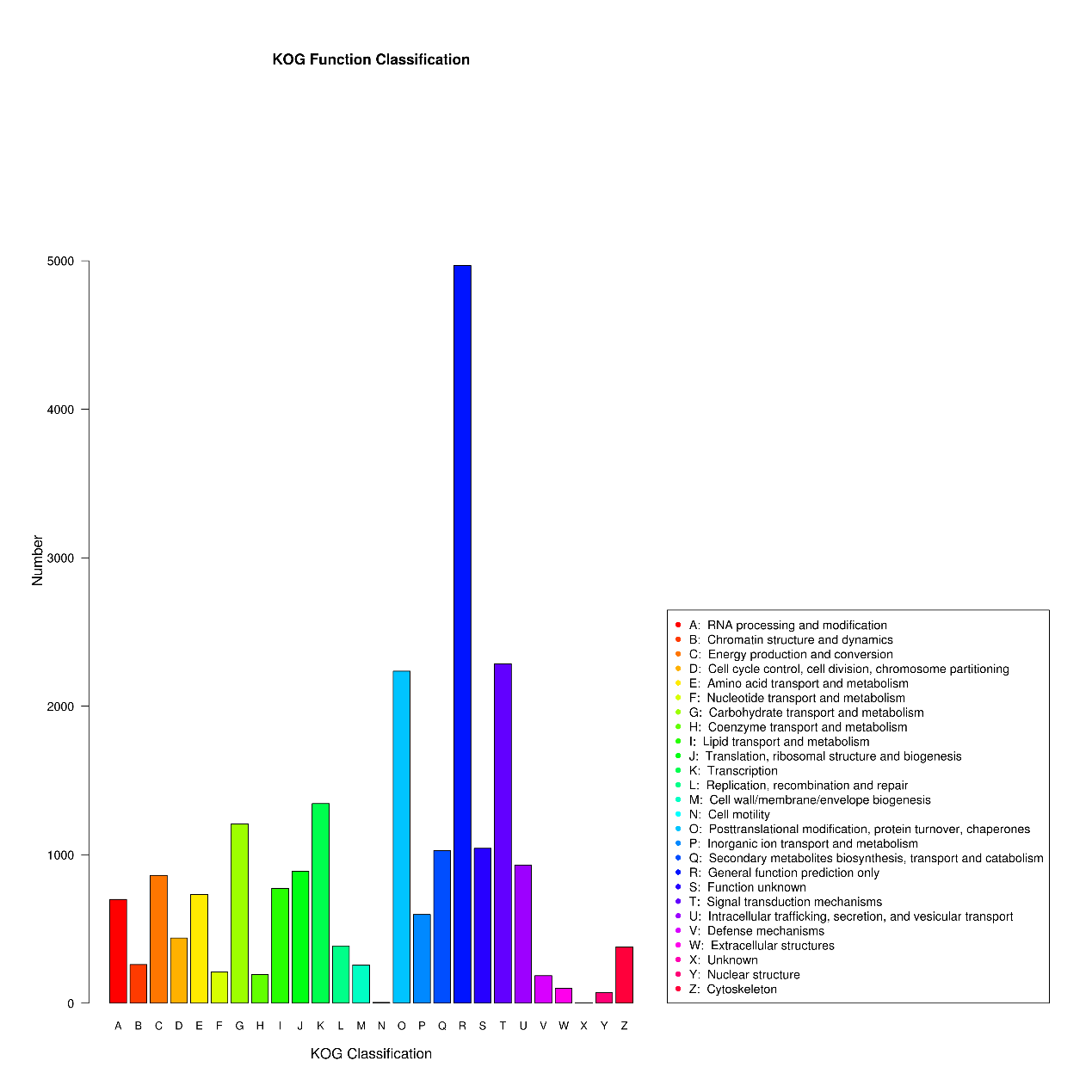


Figure S12. KOG functional classification of genes in *Hevea brasiliensis* genome.


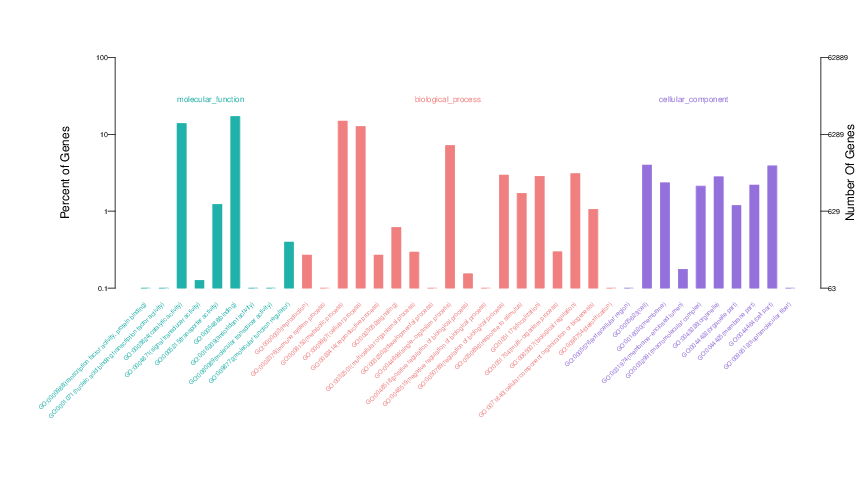


Figure S13. Gene ontology classification of genes in *Hevea brasiliensis* genome.


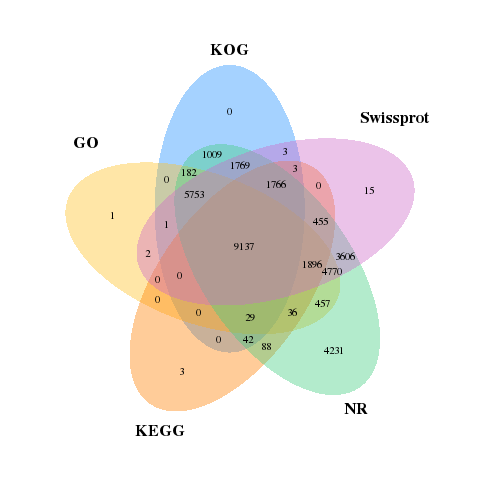


Figure S14. Venn diagram of rubber tree genes annotation between NR, GO, KOG, KEGG, Swissprot databases.


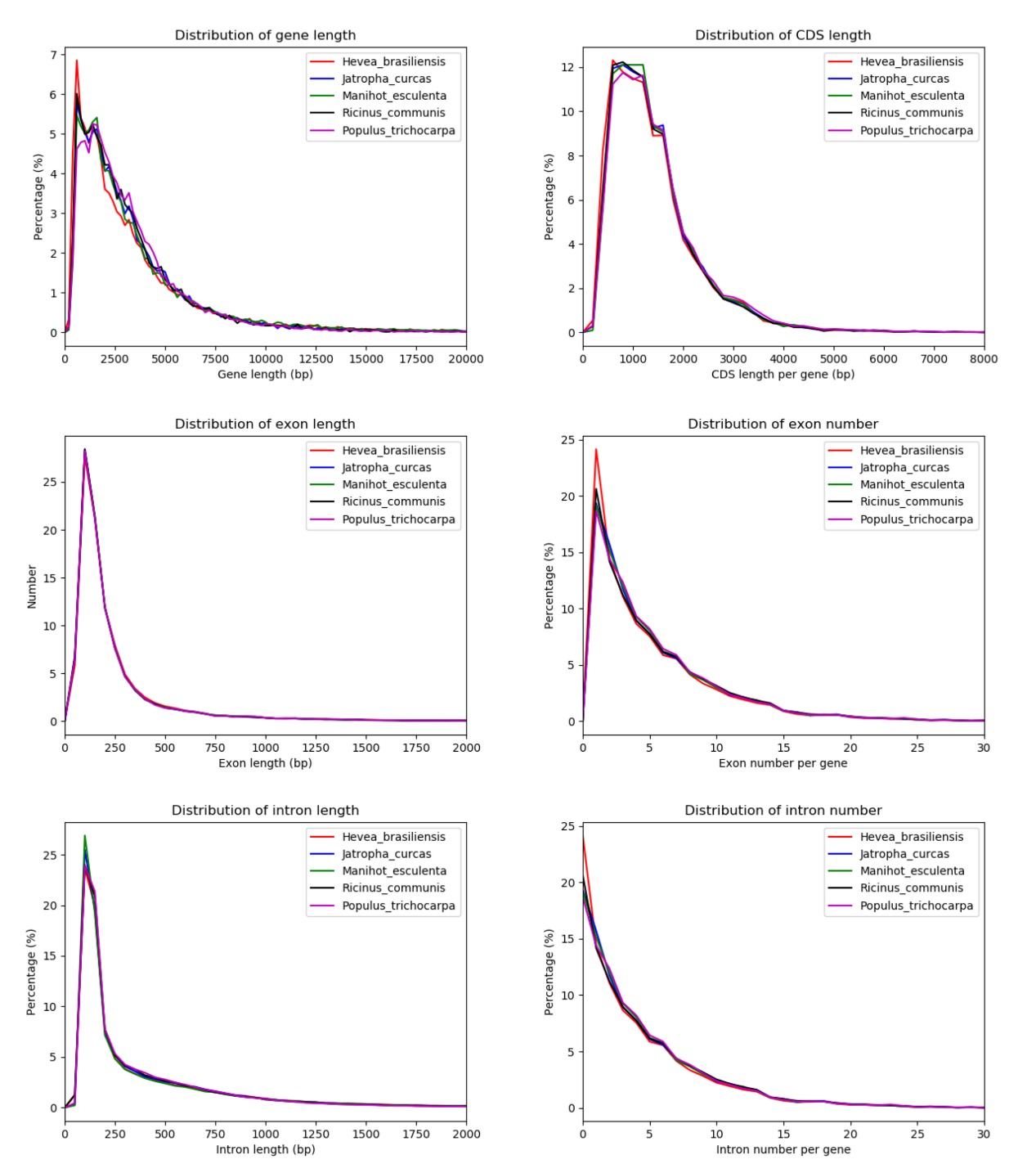


Figure S15. Cross-species comparisons in the length distribution of genes, CDSs, exons, introns, and the numbers of exons and introns. Five species were compared: *Hevea brasiliensis, Jatropha curcas, Manihot esculenta, Ricinus communis, Populus trichocarpa.*

*
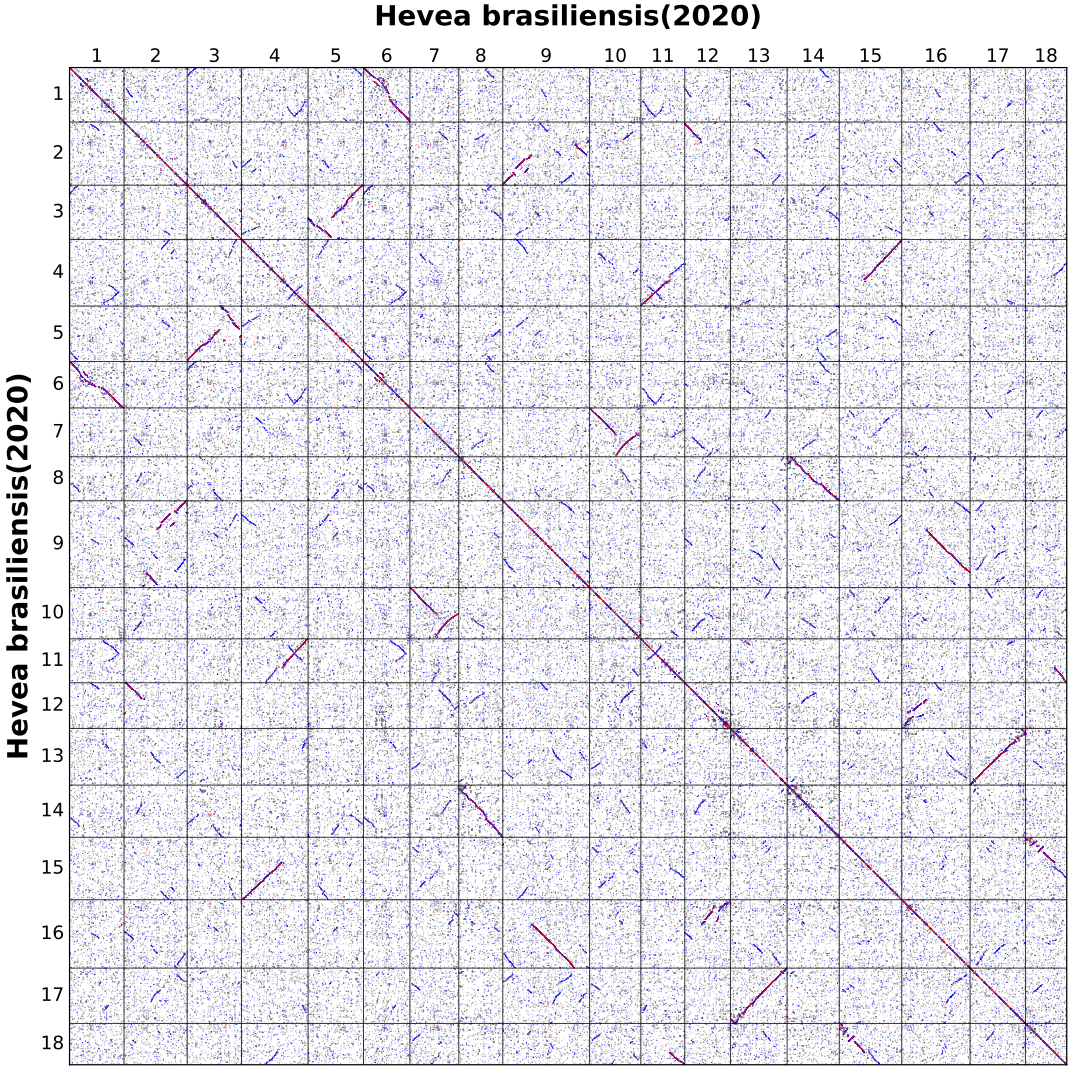
*

Figure S16. Synteny plot of the rubber tree genome.


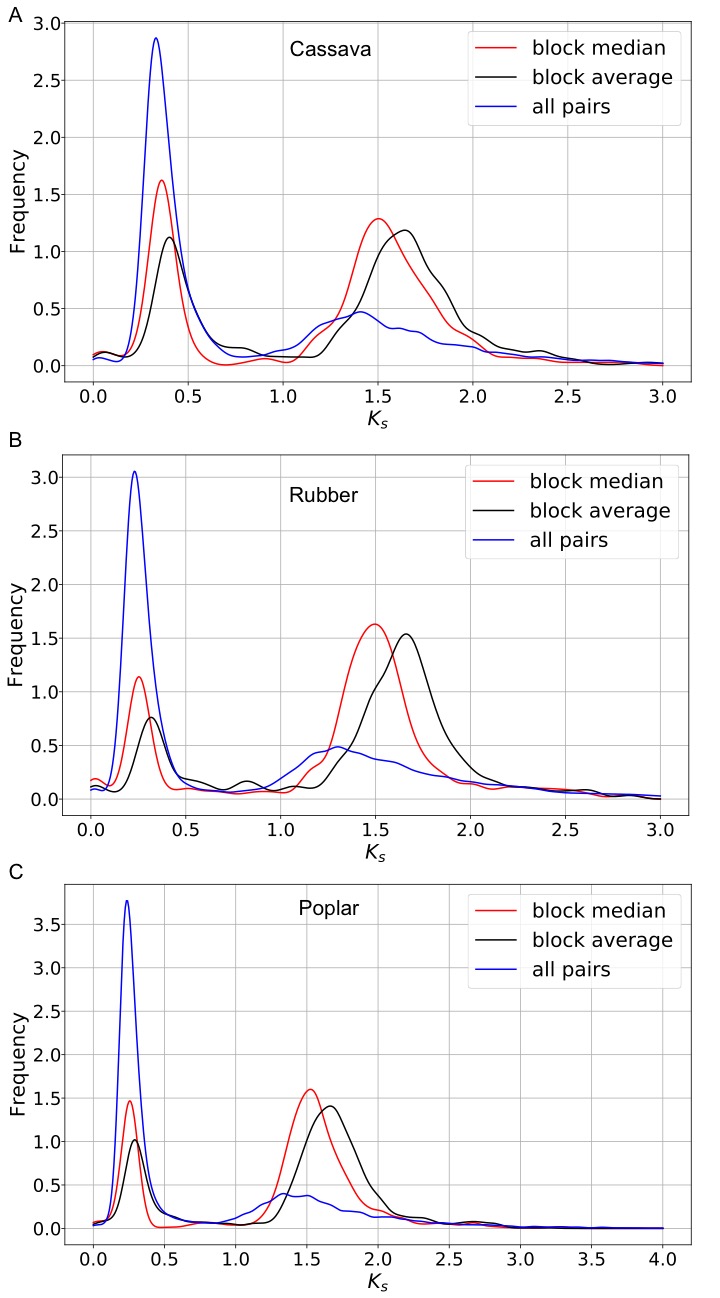


Figure S17. Ks curve of Cassava (A), Rubber tree (B) and Poplar (C).


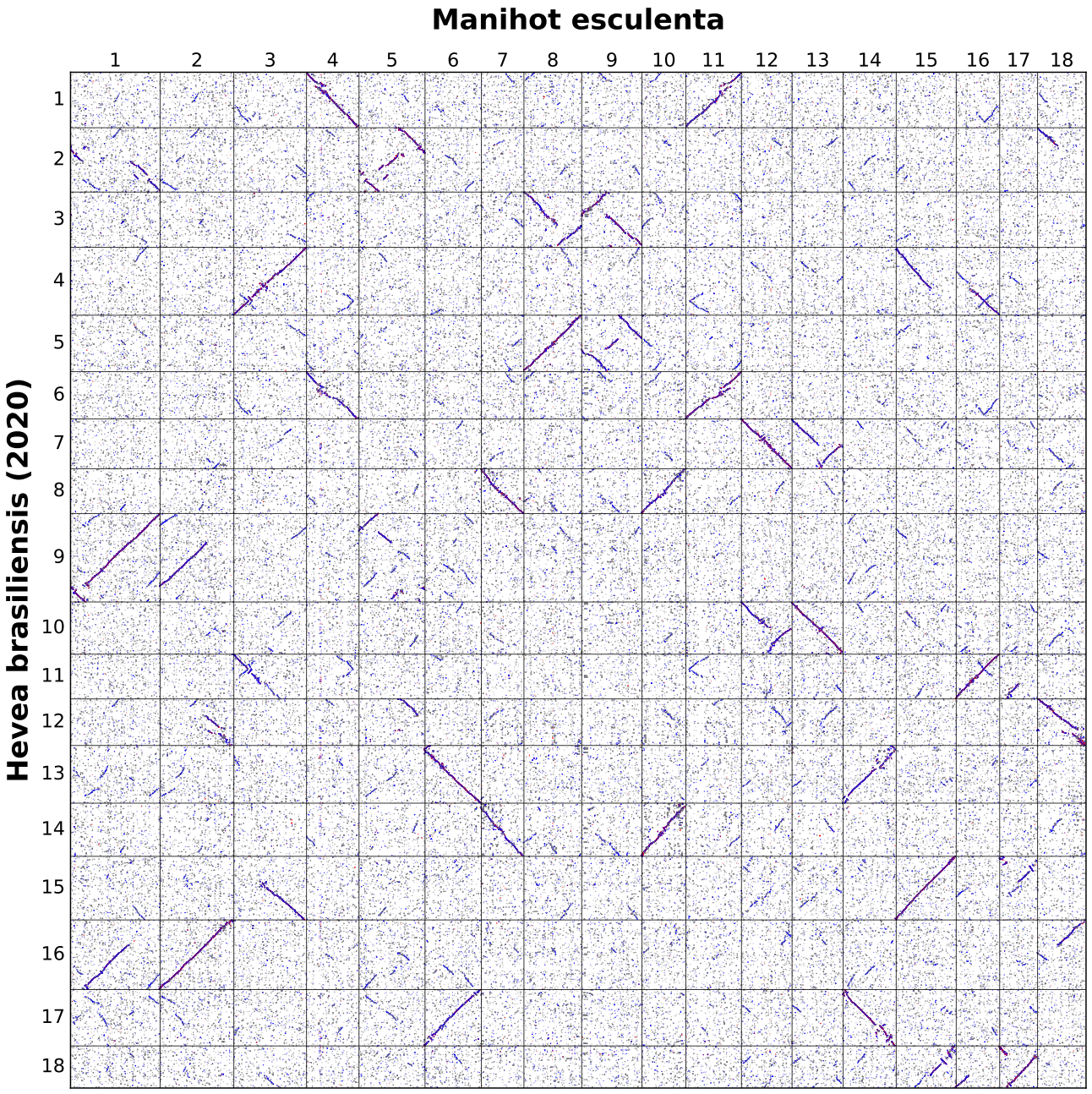


Figure S18. Synteny plot between the rubber tree and *M. esculenta* genome.


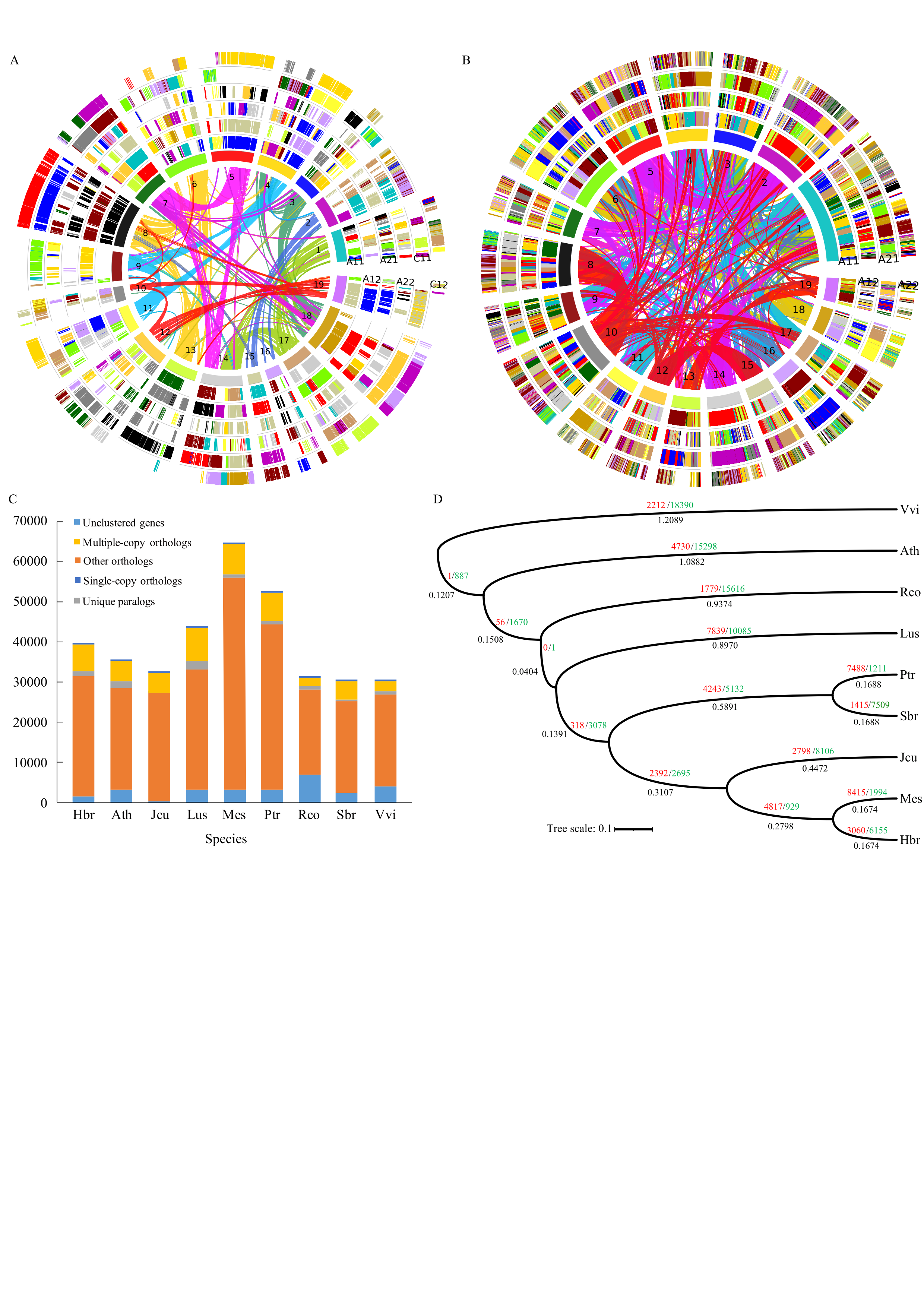


Figure S19. Genome collinearity and gene family analysis in rubber tree genome.

**(A) A map of grape–cassava–rubber multi-genomes.** The innermost circle of 19 colors represents the chromosomes of the grape. Each curve represents a pair of colinear paralog genes in grape. The outer layer of the circle: A11 and A12 represent two sets of orthologous genes in cassava. A21 and A22 represent two sets of orthologous genes in rubber (2019). C11 and C12 indicate two sets of orthologous genes in rubber (2020). Therefore, the three genomes produce 6 circles, and each circle is colored based on the positional relationship between the orthologous genes and grape chromosomes. **(B) Poplar–cassava–rubber multi-genome map.** The innermost circle with 19 colors represents the 19 chromosomes of poplar. Each curve represents a pair of paralogous genes in poplar. A11 and A12 are two sets of orthologous genes in cassava (corresponding to poplar chromosomes). A21 and A22 represent two sets of orthologous genes in rubber (corresponding to poplar chromosomes). **(C) Distribution of gene families. (D) Evolutionary tree of gene family expansion, contraction, and divergence time analysis**. The red numbers indicate the number of gene families that have been amplified during the evolution of the species, and the green numbers indicate the number of gene families that have shrunk. Hbr, *Hevea brasiliensis*; Ath, *Arabidopsis thaliana*; Jcu, *Jatropha curcas*; Lus, *Linum usitatissimum*; Mes, *Manihot esculenta*; Ptr, *Populus trichocarpa*; Rco, *Ricinus communis*; Sbr, *Salix brachista*; Vvi, *Vitis vinifera*.

Figure S20. Different types of homologous gene evolutionary trees of rubber tree, cassava, and poplar.

Figure S21. Evolutionary tree of gene family expansion and contraction. The red numbers indicate the number of gene families that have been amplified during the evolution of the species, and the green numbers indicate the number of gene families that have shrunk.


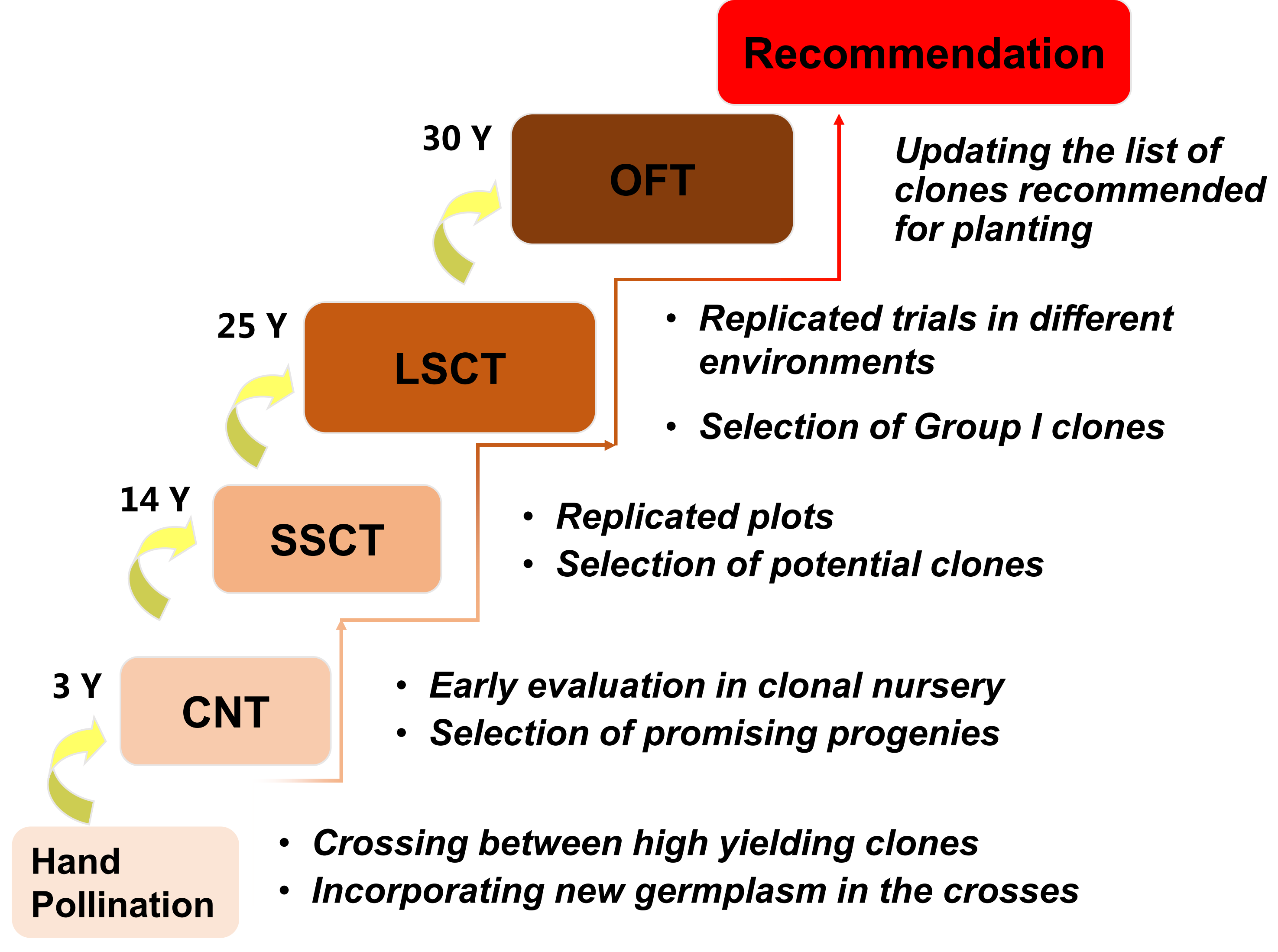


Figure S22. Diagram showing the program for the rubber tree conventional breeding. The total process takes more than 30 years. This diagram was modified from Ramli et al 2018.


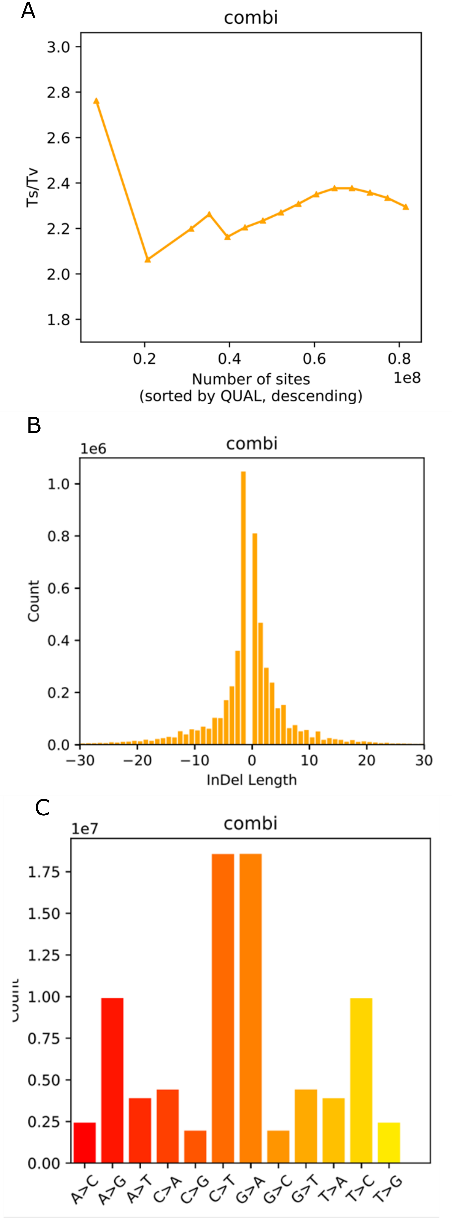


Figure S23. Statistics of polymorphisms loci identified from the population. A, Ts/Tv ratio stratified by QUAL; B, InDel length distribution; C, Nucleotide substitution frequencies.


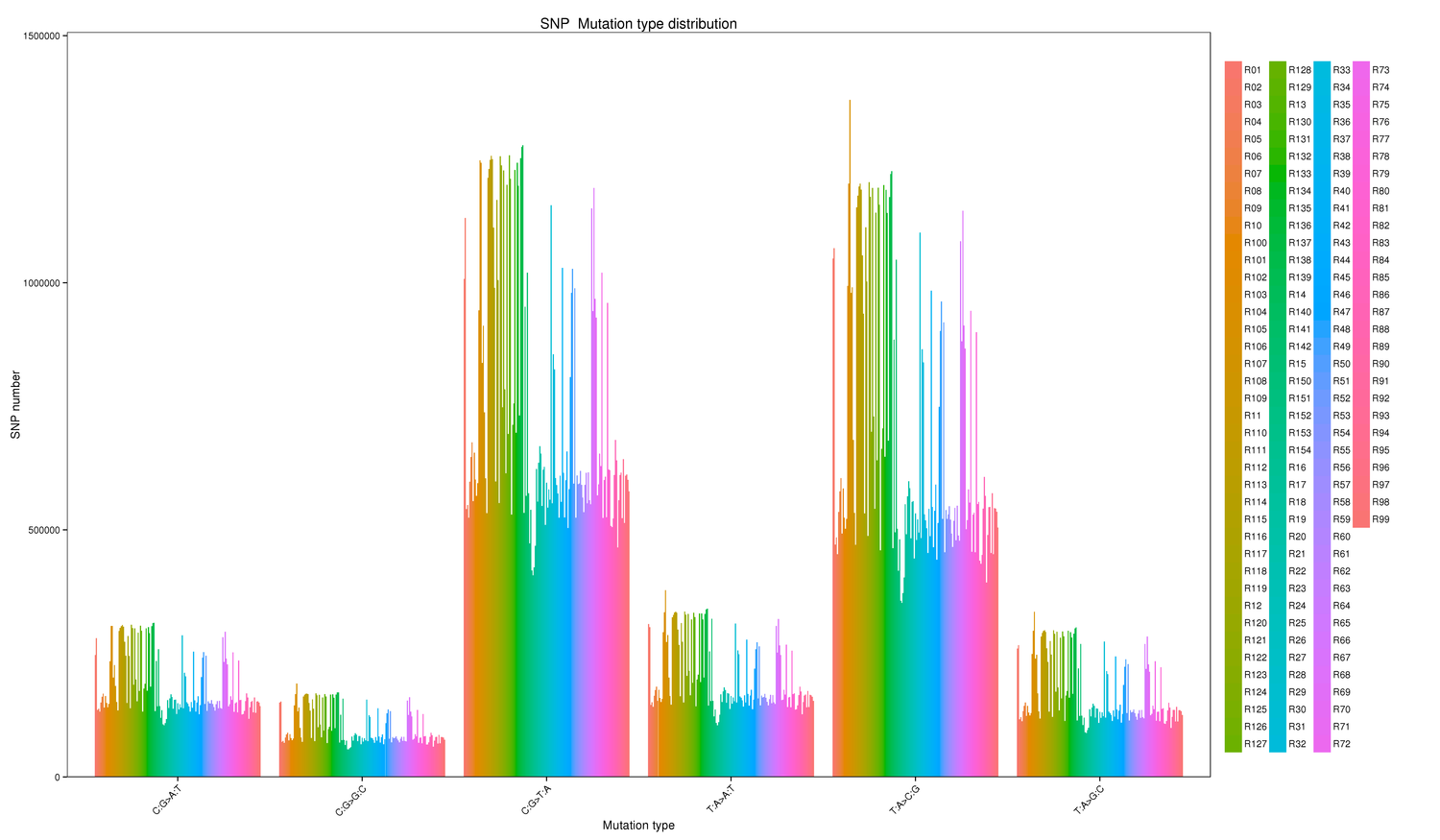


Figure S24. The distribution of SNP mutation types in the rubber tree accessions.


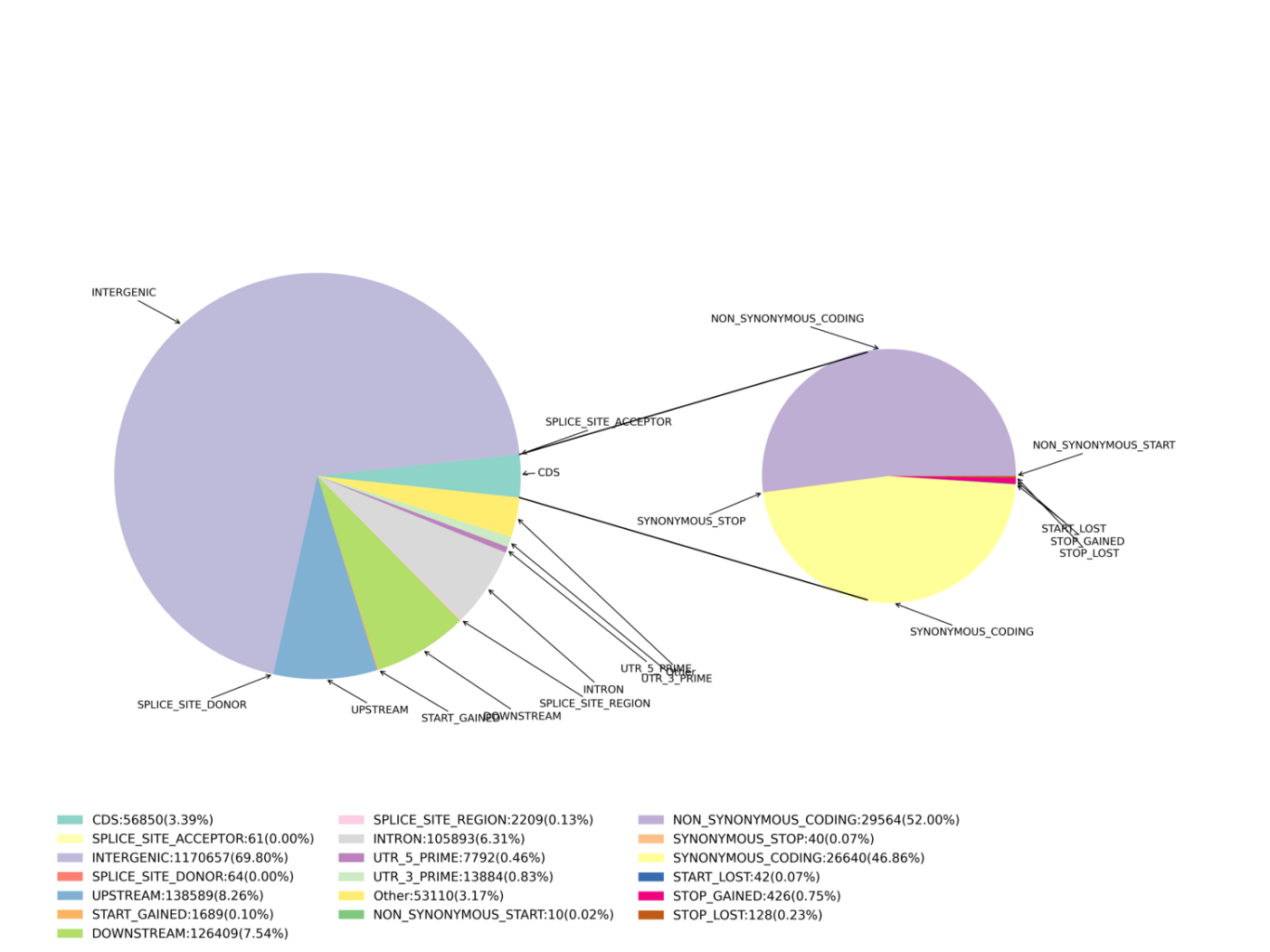


Figure S25. The annotation of SNP mutations.

Figure S26. The Percentage variance explained values of each component from PCA results.


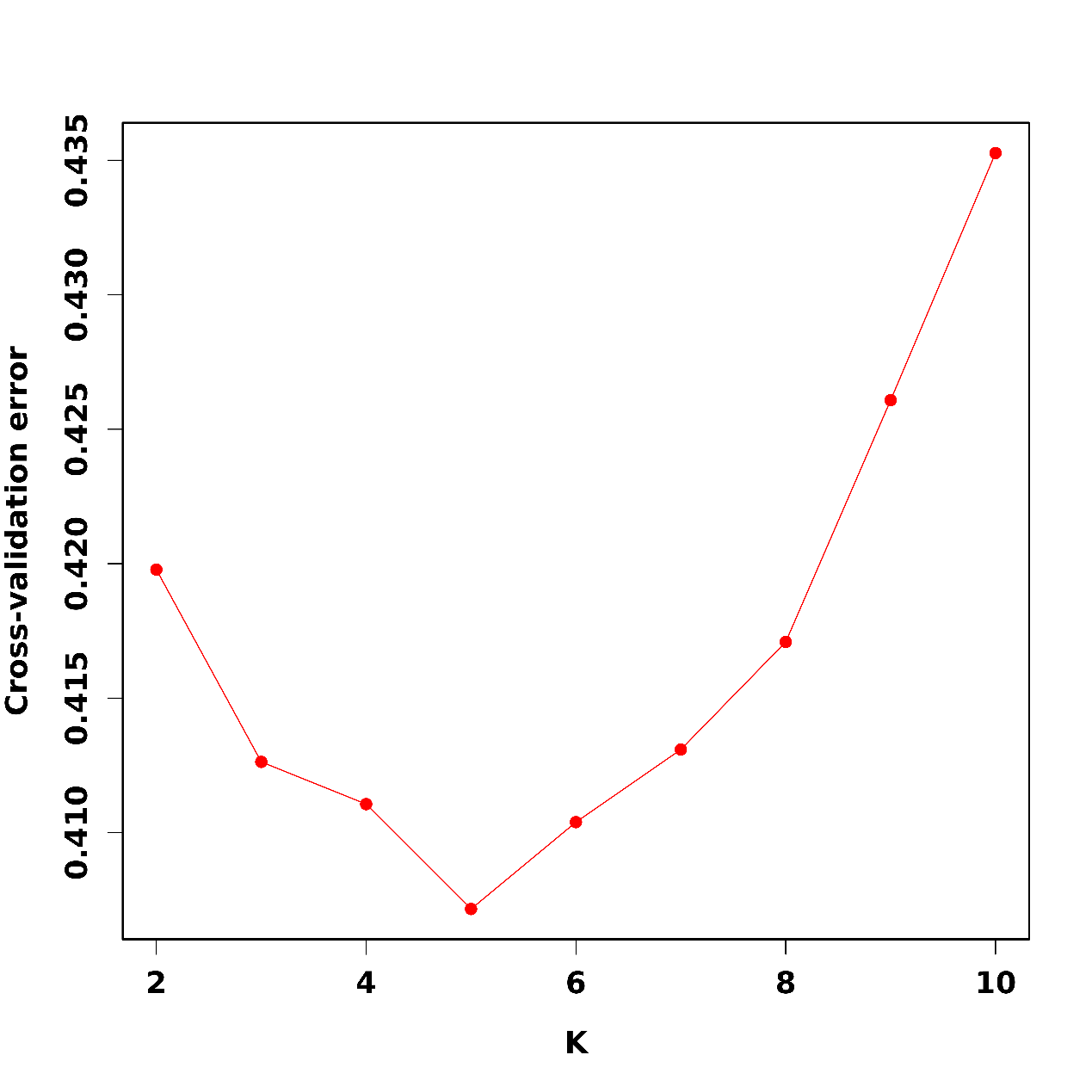


Figure S27. (CV) plot for K = 2 to K = 10 in rubber tree accessions using Admixture.


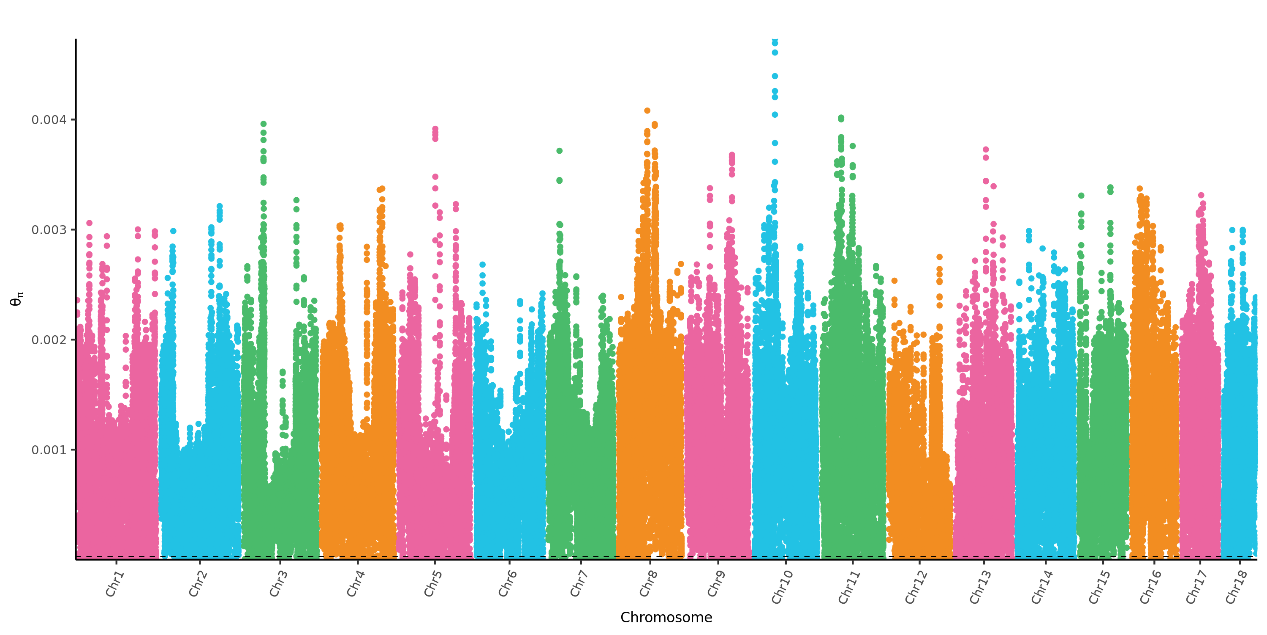


Figure S28. Manhattan plot of genome-wide nucleotide diversity (π) of Wickham accessions on each of the 18 chromosomes.


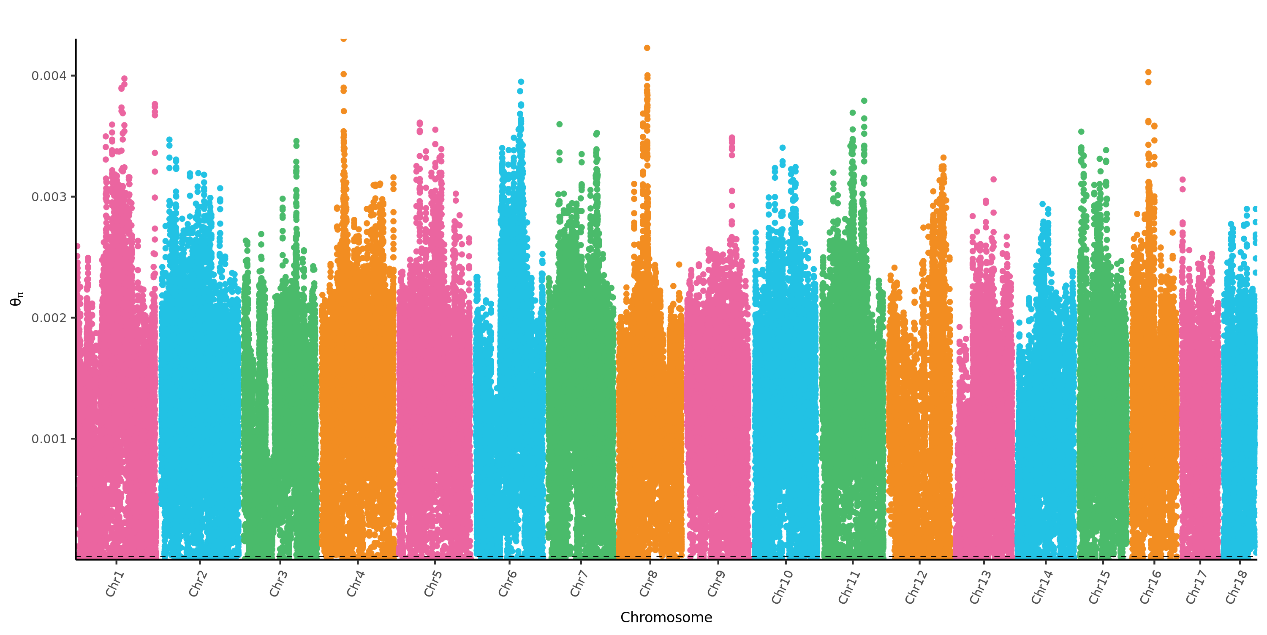


Figure S29. Manhattan plot of genome-wide nucleotide diversity (π) of Wild accessions on each of the 18 chromosomes.


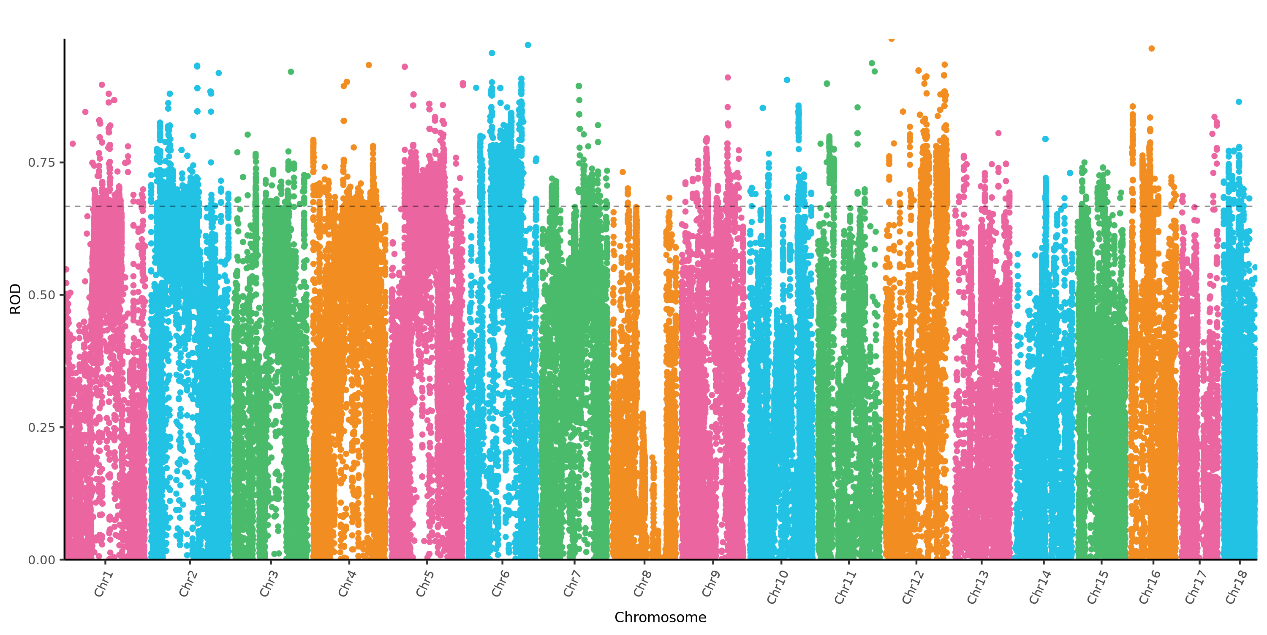


Figure S30. Manhattan plot of genome-wide reduction of diversity (ROD) between wild and Wickham accessions on each of the 18 chromosomes. Dotted line, 1% threshold


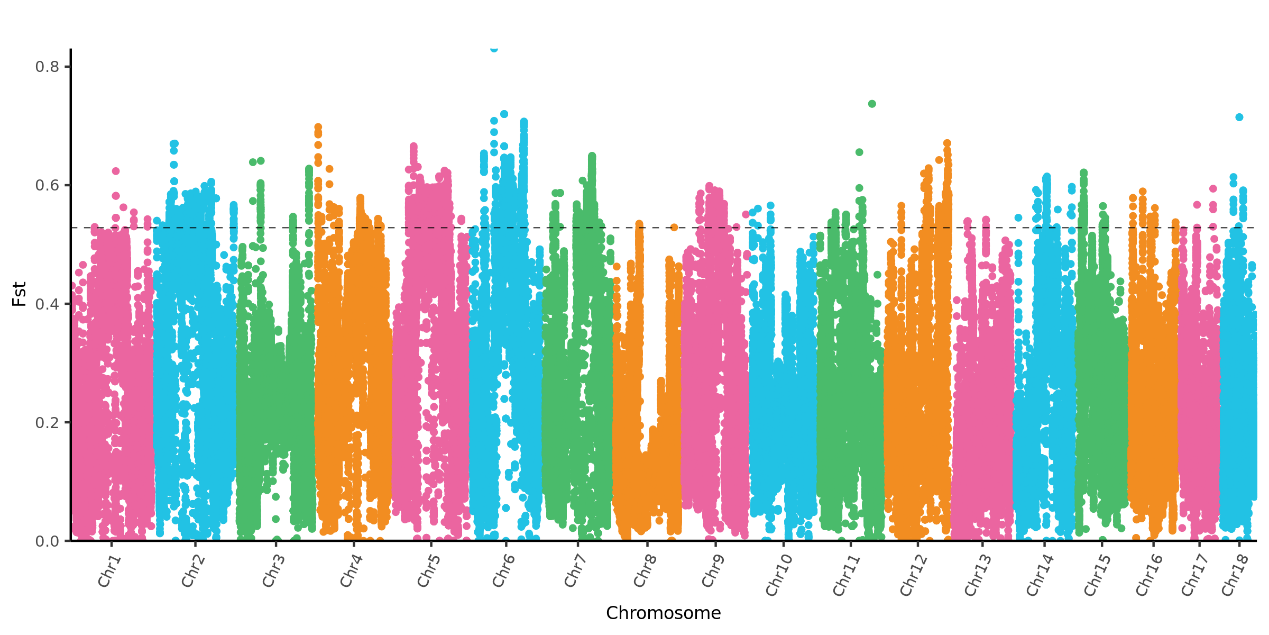


Figure S31. Manhattan plot of genome-wide Fst between wild and Wickham accessions on each of the 18 chromosomes. Dotted line, 1% threshold.


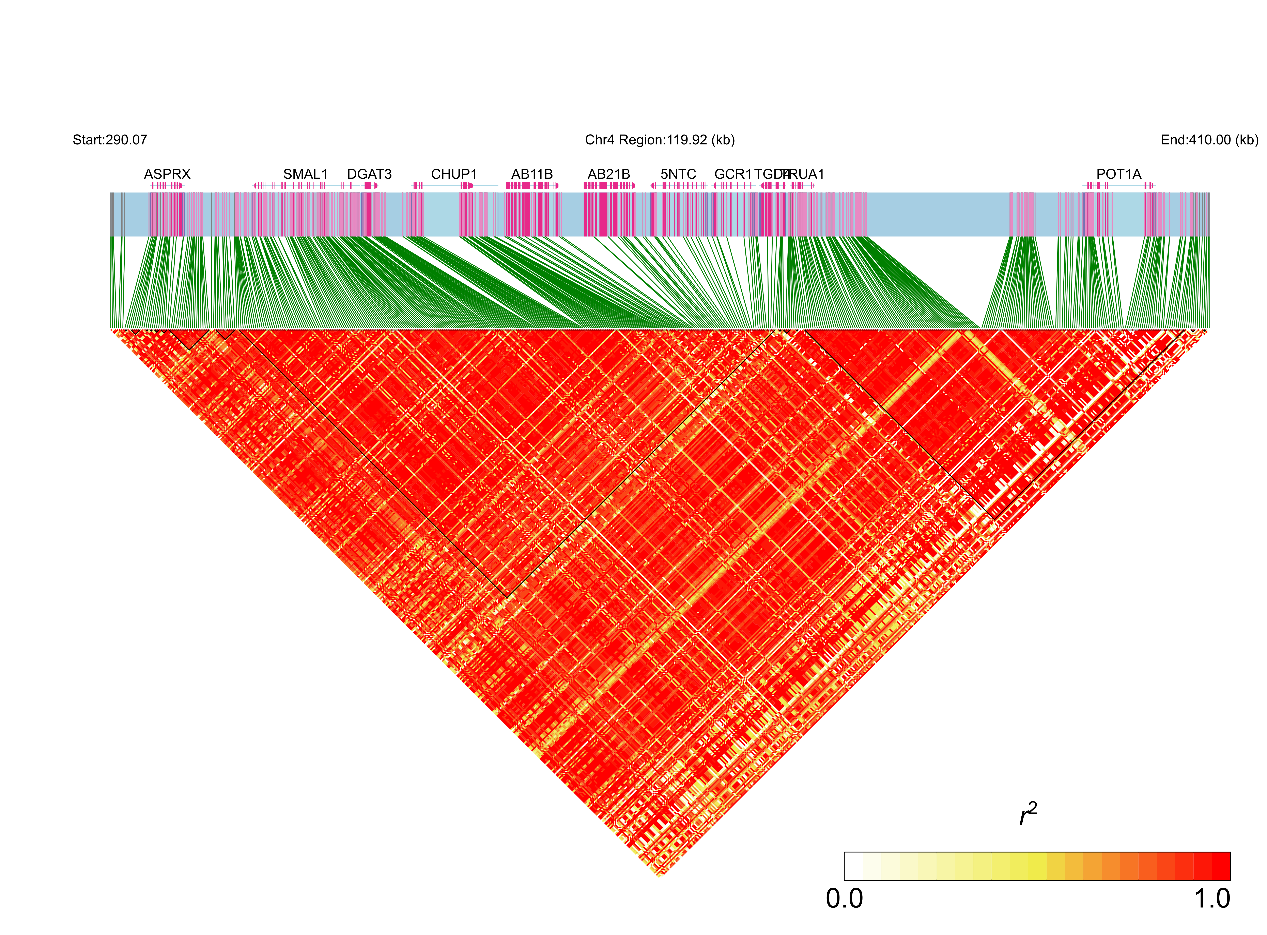


Figure S32. Linkage disequilibrium of all SNPs in the selective region on Chr4 (290001-410000) corresponding the red dots on Figure 5B.


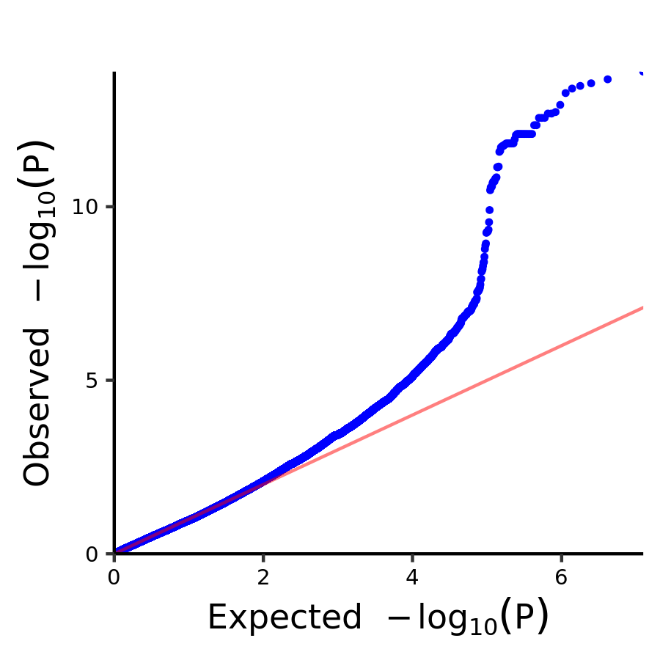


Figure S33. Q-Q plot displaying the GWAS results for latex yield using a SUPER model.


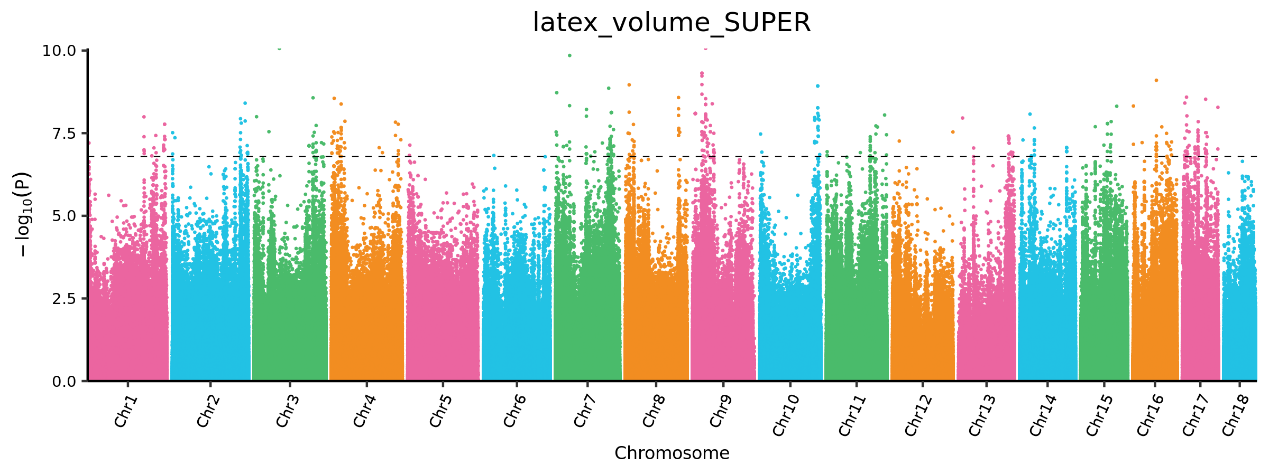


Figure S34. Manhattan plot displaying the GWAS results for latex yield using a SUPER model. The threshold for significance is *P*-value < 1.60e-07 (-log_10_*P*> 6.7)


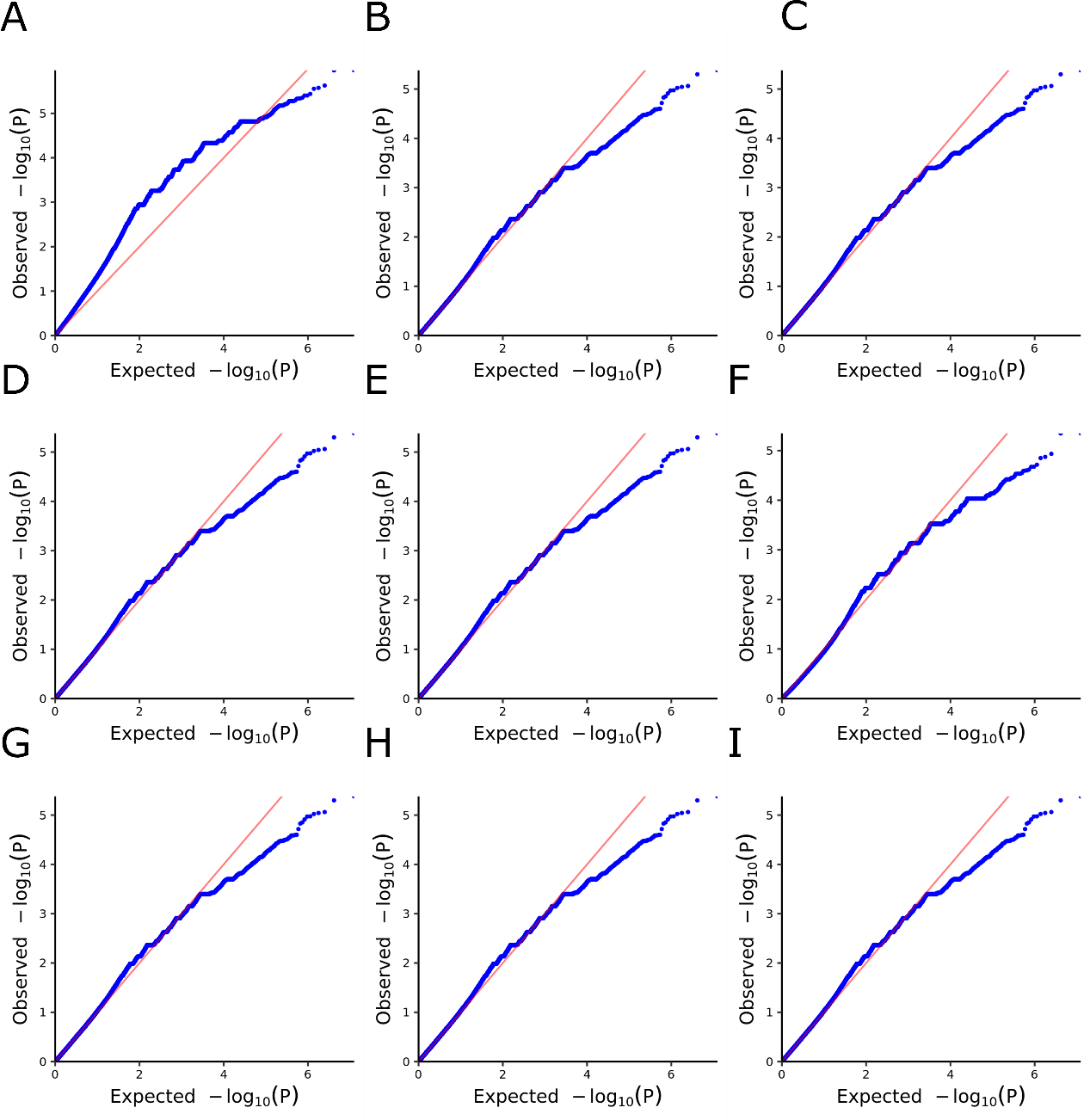


Figure S35. Q-Q plot displaying the GWAS results for latex yield using: A, GLM; B, MLM; C, MLMLM; D, CMLM; E, ECMLM; F, Blink; G, FaST; H, EMMA; and I, EMMAx models respectively.


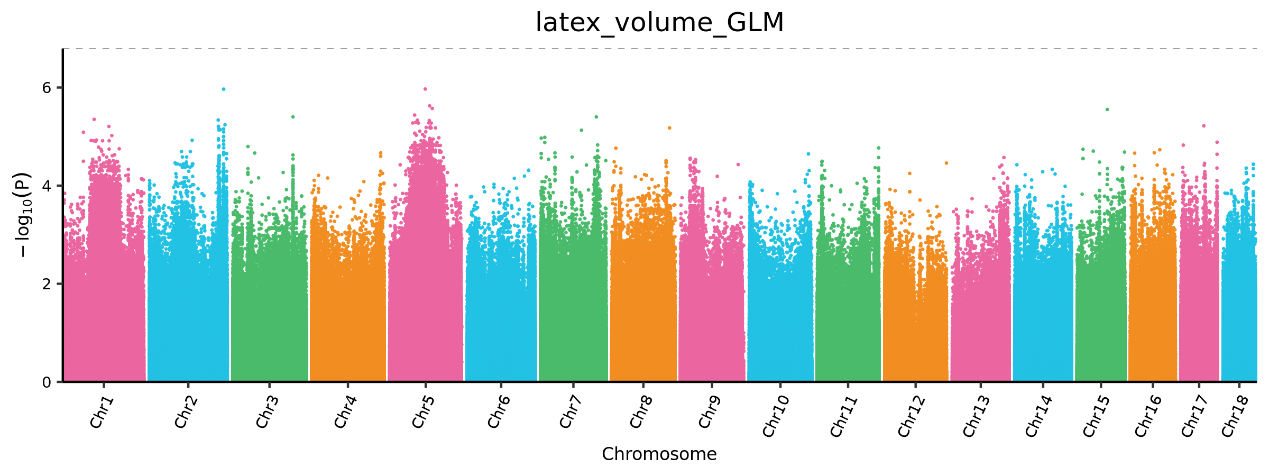

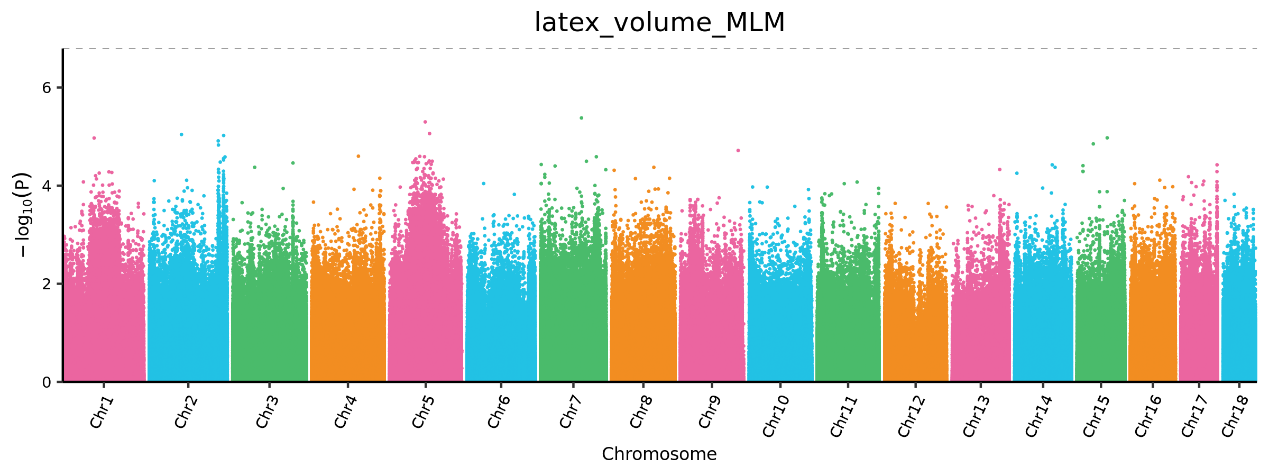

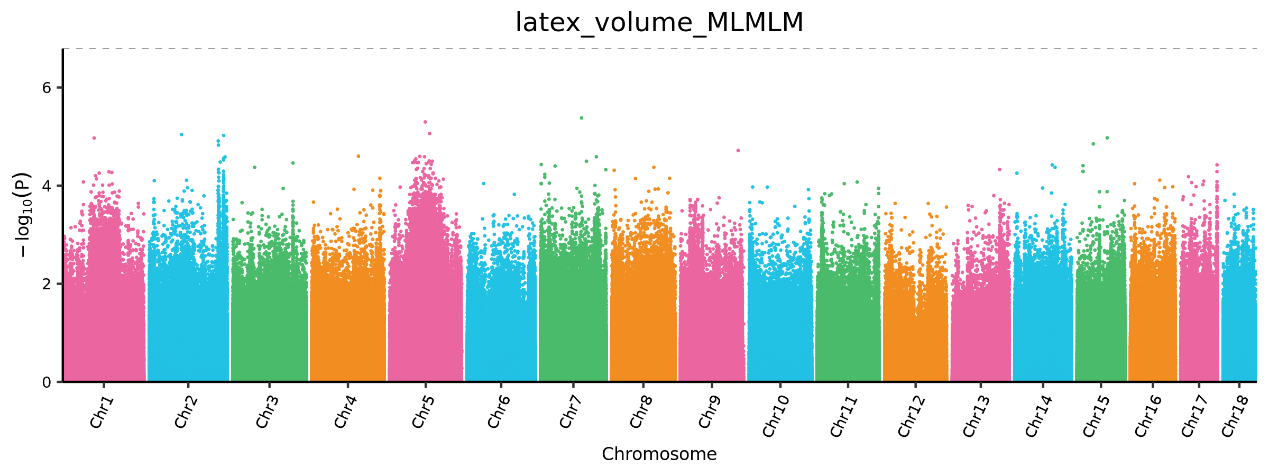

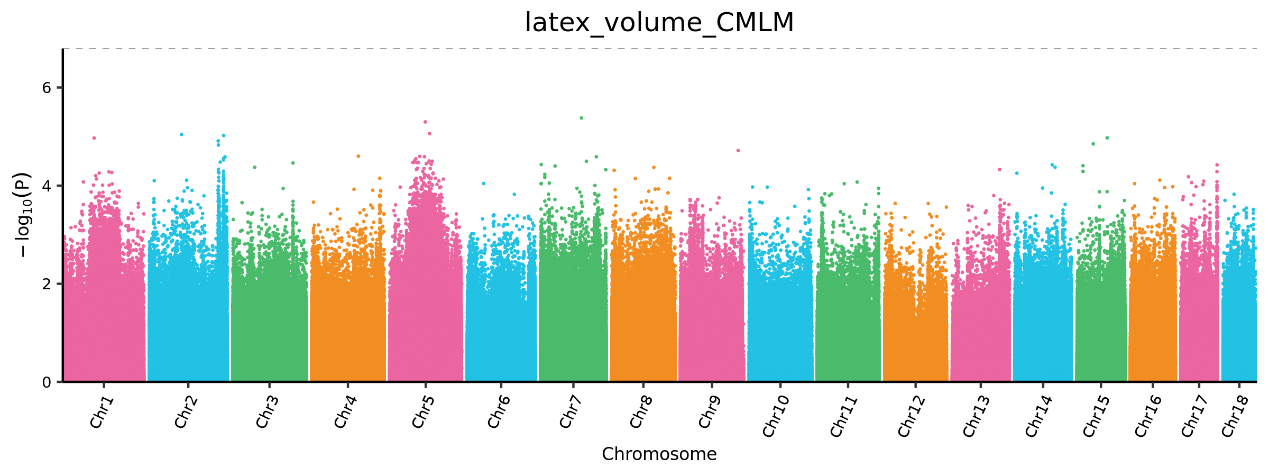

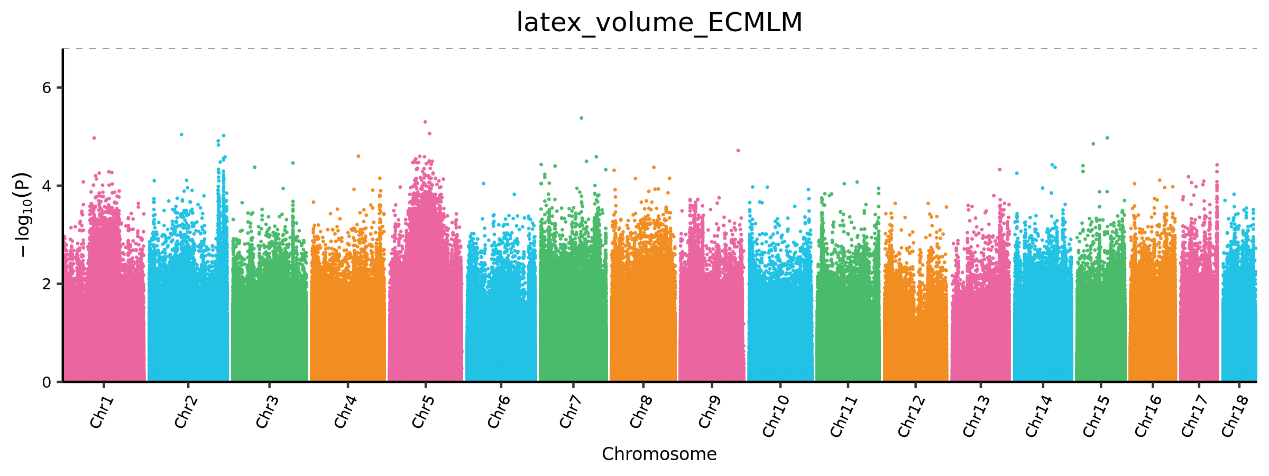

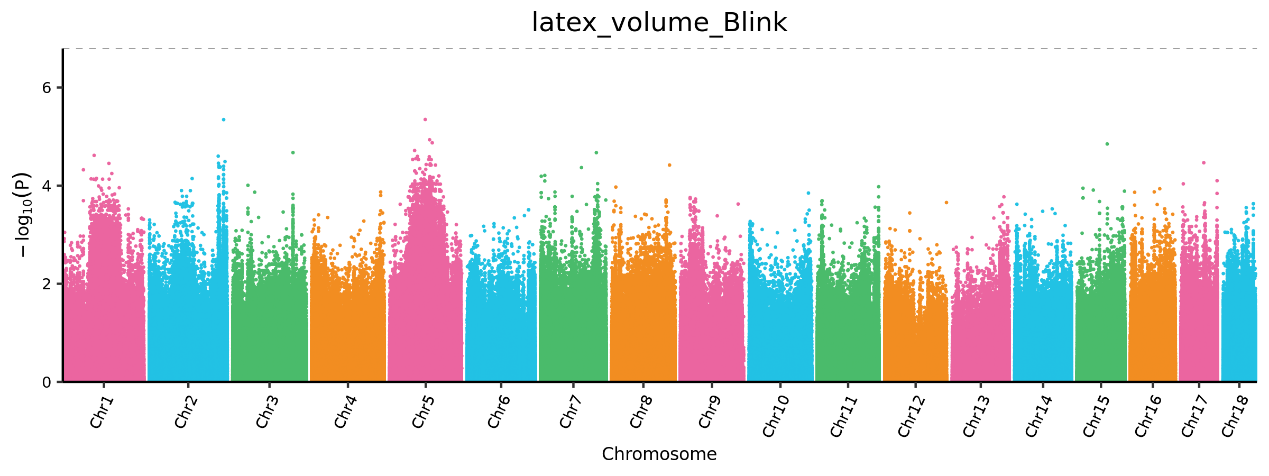

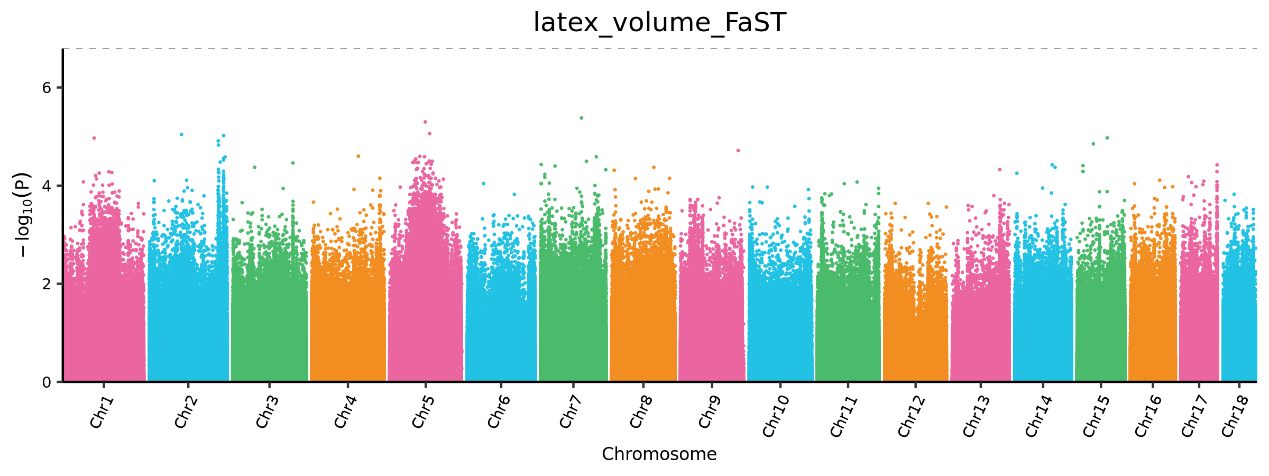

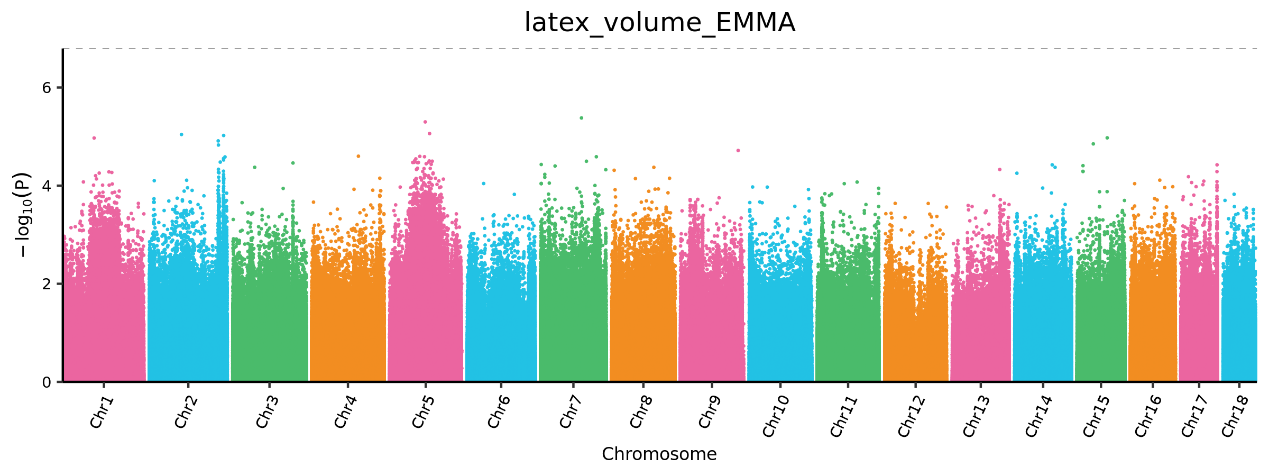


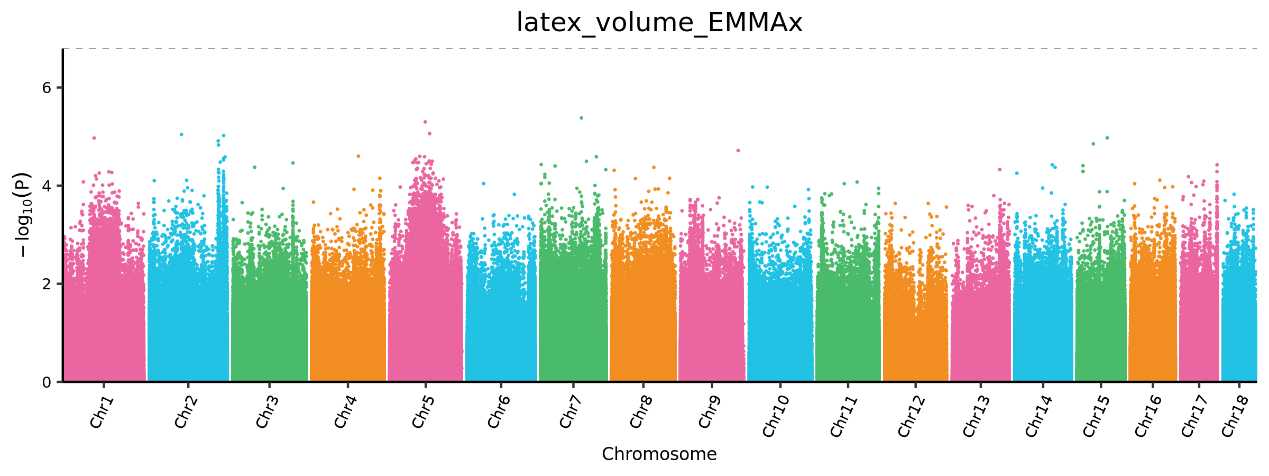


Figure S36. Manhattan plot displaying the GWAS results for latex yield using GLM, MLM, MLMLM, CMLM, ECMLM, Blink, FaST, EMMA, and EMMAx models respectively. The threshold for significance is *P*-value < 1.60e-07 (-log_10_*P*> 6.7)

Reference

Abrusán, G., Grundmann, N., DeMester, L., and Makalowski, W. (2009) *TEclass--a tool for automated classification of unknown eukaryotic transposable elements*. *Bioinformatics*, **25**, 1329–1330.

Ashburner, M., Ball, C.A., Blake, J.A., Botstein, D., Butler, H., Cherry, J.M., et al. (2000) *Gene ontology: tool for the unification of biology. The Gene Ontology Consortium*. *Nat Genet*, **25**, 25–29.

Belton, J.-M., McCord, R.P., Gibcus, J.H., Naumova, N., Zhan, Y., and Dekker, J. (2012) *Hi–C: A comprehensive technique to capture the conformation of genomes*. *Methods*, **58**, 268–276.

Benjamini, Y. and Hochberg, Y. (1995) *Controlling the False Discovery Rate: A Practical and Powerful Approach to Multiple Testing*. *Journal of the Royal Statistical Society. Series B (Methodological)*, **57**, 289–300.

Benson, G. (1999) *Tandem repeats finder: a program to analyze DNA sequences*. *Nucleic Acids Research*, **27**, 573–580.

Bolger, A.M., Lohse, M., and Usadel, B. (2014) *Trimmomatic: a flexible trimmer for Illumina sequence data*. *Bioinformatics*, **30**, 2114–2120.

Burton, J.N., Adey, A., Patwardhan, R.P., Qiu, R., Kitzman, J.O., and Shendure, J. (2013) *Chromosome-scale scaffolding of de novo genome assemblies based on chromatin interactions*. *Nat Biotechnol*, **31**, 1119–1125.

Chan, P.P. and Lowe, T.M. (2019) *tRNAscan-SE: Searching for tRNA Genes in Genomic Sequences*. *Methods Mol Biol*, **1962**, 1–14.

Chen, S., Zhou, Y., Chen, Y., and Gu, J. (2018) *fastp: an ultra-fast all-in-one FASTQ preprocessor*. *Bioinformatics*, **34**, i884–i890.

Cingolani, P., Platts, A., Wang, L.L., Coon, M., Nguyen, T., Wang, L., et al. (2012) *A program for annotating and predicting the effects of single nucleotide polymorphisms, SnpEff: SNPs in the genome of Drosophila melanogaster strain w ^1118^ ; iso-2; iso-3*. *Fly*, **6**, 80–92.

Danecek, P., Auton, A., Abecasis, G., Albers, C.A., Banks, E., DePristo, M.A., et al. (2011) *The variant call format and VCFtools*. *Bioinformatics*, **27**, 2156–2158.

Danecek, P., Bonfield, J.K., Liddle, J., Marshall, J., Ohan, V., Pollard, M.O., et al. (2021) *Twelve years of SAMtools and BCFtools*. *Gigascience*, **10**.

Dh, A., J, N., and K, L. (2009) *Fast model-based estimation of ancestry in unrelated individuals.* *Genome Res*, **19**, 1655–1664.

Dobin, A., Davis, C.A., Schlesinger, F., Drenkow, J., Zaleski, C., Jha, S., et al. (2013) *STAR: ultrafast universal RNA-seq aligner*. *Bioinformatics*, **29**, 15–21.

Flynn, J.M., Hubley, R., Goubert, C., Rosen, J., Clark, A.G., Feschotte, C., and Smit, A.F. (2020) *RepeatModeler2 for automated genomic discovery of transposable element families*. *Proc Natl Acad Sci U S A*, **117**, 9451–9457.

Galperin, M.Y., Makarova, K.S., Wolf, Y.I., and Koonin, E.V. (2015) *Expanded microbial genome coverage and improved protein family annotation in the COG database*. *Nucleic Acids Research*, **43**, D261–D269.

Griffiths-Jones, S., Moxon, S., Marshall, M., Khanna, A., Eddy, S.R., and Bateman, A. (2005) *Rfam: annotating non-coding RNAs in complete genomes*. *Nucleic Acids Research*, **33**, D121–D124.

Haas, B.J., Delcher, A.L., Mount, S.M., Wortman, J.R., Smith, R.K., Hannick, L.I., et al. (2003) *Improving the Arabidopsis genome annotation using maximal transcript alignment assemblies*. *Nucleic Acids Res*, **31**, 5654–5666.

Haas, B.J., Salzberg, S.L., Zhu, W., Pertea, M., Allen, J.E., Orvis, J., et al. (2008) *Automated eukaryotic gene structure annotation using EVidenceModeler and the Program to Assemble Spliced Alignments*. *Genome Biol*, **9**, R7.

Han, Y. and Wessler, S.R. (2010) *MITE-Hunter: a program for discovering miniature inverted-repeat transposable elements from genomic sequences*. *Nucleic Acids Res*, **38**, e199.

Jaillon, O., Aury, J.-M., Noel, B., Policriti, A., Clepet, C., Casagrande, A., et al. (2007) *The grapevine genome sequence suggests ancestral hexaploidization in major angiosperm phyla*. *Nature*, **449**, 463–467.

Jurka, J., Kapitonov, V.V., Pavlicek, A., Klonowski, P., Kohany, O., and Walichiewicz, J. (2005) *Repbase Update, a database of eukaryotic repetitive elements*. *Cytogenet Genome Res*, **110**, 462–467.

Keilwagen, J., Hartung, F., and Grau, J. (2019) *GeMoMa: Homology-Based Gene Prediction Utilizing Intron Position Conservation and RNA-seq Data*. In: *Gene Prediction* Methods in Molecular Biology (Kollmar,M., ed) , pp. 161–177. New York, NY: Springer New York.

Kumar, S., Stecher, G., Peterson, D., and Tamura, K. (2012) *MEGA-CC: computing core of molecular evolutionary genetics analysis program for automated and iterative data analysis*. *Bioinformatics*, **28**, 2685–2686.

Kumar, S., Stecher, G., Suleski, M., and Hedges, S.B. (2017) *TimeTree: A Resource for Timelines, Timetrees, and Divergence Times*. *Molecular Biology and Evolution*, **34**, 1812–1819.

Lagesen, K., Hallin, P., Rødland, E.A., Stærfeldt, H.-H., Rognes, T., and Ussery, D.W. (2007) *RNAmmer: consistent and rapid annotation of ribosomal RNA genes*. *Nucleic Acids Research*, **35**, 3100–3108.

Langmead, B. and Salzberg, S.L. (2012) *Fast gapped-read alignment with Bowtie 2*. *Nat Methods*, **9**, 357–359.

Li, H. and Durbin, R. (2009) *Fast and accurate short read alignment with Burrows-Wheeler transform*. *Bioinformatics*, **25**, 1754–1760.

Li, H., Handsaker, B., Wysoker, A., Fennell, T., Ruan, J., Homer, N., et al. (2009) *The Sequence Alignment/Map format and SAMtools*. *Bioinformatics*, **25**, 2078–2079.

Lomsadze, A., Burns, P.D., and Borodovsky, M. (2014) *Integration of mapped RNA-Seq reads into automatic training of eukaryotic gene finding algorithm*. *Nucleic Acids Research*, **42**, e119–e119.

Maere, S., Heymans, K., and Kuiper, M. (2005) *BiNGO: a Cytoscape plugin to assess overrepresentation of Gene Ontology categories in Biological Networks*. *Bioinformatics*, **21**, 3448–3449.

McKenna, A., Hanna, M., Banks, E., Sivachenko, A., Cibulskis, K., Kernytsky, A., et al. (2010) *The Genome Analysis Toolkit: a MapReduce framework for analyzing next-generation DNA sequencing data*. *Genome Res*, **20**, 1297–1303.

Moriya, Y., Itoh, M., Okuda, S., Yoshizawa, A.C., and Kanehisa, M. (2007) *KAAS: an automatic genome annotation and pathway reconstruction server*. *Nucleic Acids Res*, **35**, W182–W185.

Nawrocki, E.P. and Eddy, S.R. (2013) *Infernal 1.1: 100-fold faster RNA homology searches*. *Bioinformatics*, **29**, 2933–2935.

Othman, R., Benong, M., Aris, M.N.M., and Ghani, Z.Ab. (2004) *IRRDB 1981 EXPEDITION: HARNESSING GENETIC POTENTIAL OF HEVEA GERMPLASM*. In: , pp. 11–25.

Ou, S. and Jiang, N. (2018) *LTR_retriever: A Highly Accurate and Sensitive Program for Identification of Long Terminal Repeat Retrotransposons*. *Plant Physiol*, **176**, 1410–1422.

Parra, G., Bradnam, K., and Korf, I. (2007) *CEGMA: a pipeline to accurately annotate core genes in eukaryotic genomes*. *Bioinformatics*, **23**, 1061–1067.

Patel, R.K. and Jain, M. (2012) *NGS QC Toolkit: a toolkit for quality control of next generation sequencing data*. *PLoS One*, **7**, e30619.

Pertea, M., Kim, D., Pertea, G.M., Leek, J.T., and Salzberg, S.L. (2016) *Transcript-level expression analysis of RNA-seq experiments with HISAT, StringTie and Ballgown*. *Nature Protocols*, **11**, 1650–1667.

Price, A.L., Patterson, N.J., Plenge, R.M., Weinblatt, M.E., Shadick, N.A., and Reich, D. (2006) *Principal components analysis corrects for stratification in genome-wide association studies*. *Nature Genetics*, **38**, 904–909.

Simão, F.A., Waterhouse, R.M., Ioannidis, P., Kriventseva, E.V., and Zdobnov, E.M. (2015) *BUSCO: assessing genome assembly and annotation completeness with single-copy orthologs*. *Bioinformatics*, **31**, 3210–3212.

Stanke, M., Steinkamp, R., Waack, S., and Morgenstern, B. (2004) *AUGUSTUS: a web server for gene finding in eukaryotes*. *Nucleic Acids Res*, **32**, W309–W312.

Stanke, M. and Waack, S. (2003) *Gene prediction with a hidden Markov model and a new intron submodel*. *Bioinformatics*, **19**, ii215–ii225.

Tamura, K., Stecher, G., Peterson, D., Filipski, A., and Kumar, S. (2013) *MEGA6: Molecular Evolutionary Genetics Analysis Version 6.0*. *Mol Biol Evol*, **30**, 2725–2729.

Tang, C., Yang, M., Fang, Y., Luo, Y., Gao, S., Xiao, X., et al. (2016) *The rubber tree genome reveals new insights into rubber production and species adaptation*. *Nature Plants*, **2**, 16073.

Tang, S., Lomsadze, A., and Borodovsky, M. (2015) *Identification of protein coding regions in RNA transcripts*. *Nucleic Acids Res*, **43**, e78.

Tarailo-Graovac, M. and Chen, N. (2009) *Using RepeatMasker to identify repetitive elements in genomic sequences*. *Curr Protoc Bioinformatics*, **Chapter 4**, Unit 4.10.

Walker, B.J., Abeel, T., Shea, T., Priest, M., Abouelliel, A., Sakthikumar, S., et al. (2014) *Pilon: An Integrated Tool for Comprehensive Microbial Variant Detection and Genome Assembly Improvement*. *PLOS ONE*, **9**, e112963.

Wang, X. and Wang, L. (2016) *GMATA: An Integrated Software Package for Genome-Scale SSR Mining, Marker Development and Viewing*. *Front. Plant Sci.*, **7**.

Wu, T.D. and Watanabe, C.K. (2005) *GMAP: a genomic mapping and alignment program for mRNA and EST sequences*. *Bioinformatics*, **21**, 1859–1875.

Yang, Z. (2007) *PAML 4: phylogenetic analysis by maximum likelihood*. *Mol. Biol. Evol.*, **24**, 1586–1591.

Zhang, C., Dong, S.-S., Xu, J.-Y., He, W.-M., and Yang, T.-L. (2019) *PopLDdecay: a fast and effective tool for linkage disequilibrium decay analysis based on variant call format files*. *Bioinformatics*, **35**, 1786–1788.
